# Supplementary material for: Parts–per–million of ruthenium catalyze the selective chain–walking reaction of terminal alkenes
Source: Nat Commun. 2022 May 20;13:2831. doi: 10.1038/s41467-022-30320-9 (PMC9123009; doi:10.1038/s41467-022-30320-9)
Supplement: Supplementary file 1 — Supplementary Information [file 41467_2022_30320_MOESM1_ESM.pdf]

Supplementary Information (SI) for the manuscript:

Parts-per-million of ruthenium catalyze the selective chain-walking reaction of terminal alkenes

Sergio Sanz-Navarro†, Marta Mon†, Antonio Doménech-Carbó, Rossella Greco, Jorge Sánchez-Quesada, Estela Espinós–Ferri, Antonio Leyva–Pérez.*

†These authors contributed equally to the work.

*Corresponding author email: anleyva@itq.upv.es.

Table of Contents

Supplementary Methods S3

Additional procedures of one-pot reactions from methyl isoeugenol **2** S3

Supplementary Discussion S7

Supplementary Figures 1-72 S7

Supplementary Tables 1-3 S51

Compound characterization S58

Supplementary References S70

Supplementary Methods

- Additional procedures of one-pot reactions from methyl isoeugenol 2.

Diacetoxylation. Following the general procedure described above for the isomerization reaction, a mixture of neat **2** (178 mg, 1 mmol) and Ru(methylallyl)$_2$(COD) (1.5 µg, 0.0005 mol%) was heated in a 8 ml vial equipped with a magnetic stir bar at 200 °C. After cooling, Pd(OAc)$_2$ (5 mg, 2 mol%), KI (33 mg, 0.2 mmol) and HOAc (3 mL) were added and the vial closed with a cap connected to a manometer and a gas exit, under N_2 atmosphere. Then, the mixture was placed in a steel block under magnetic stirring, the vial was purged three times with O_2 gas to leave a final O_2 pressure of 5 bar. The reaction mixture was stirred at 100 ºC for 24 h. After cooling, O_2 was released and the mixture was extracted with ethyl acetate and washed with aqueous $NaHCO_3$. The combined organic phases were dried over $MgSO_4$, filtered and concentrated under vacuum. Flash chromatography (10% AcOEt in hexane) gives 192 mg (65%) of **27** as a yellow oil[41].

Friedel-Crafts. Following the general procedure described above for the isomerization reaction, a mixture of neat **2** (178 mg, 1 mmol) and Ru(methylallyl)$_2$(COD) (1.5 µg, 0.0005 mol%) was heated in a 8 ml vial equipped with a magnetic stir bar at 200 °C. After cooling, AcOEt (4 ml, 0.25 M) and $Fe(ClO_4)_3·6H_2O$ (35 mg, 10 mol%) were added in one portion. The mixture was stirred at 40 ºC for 24 h under air. Then, the solvent was evaporated under reduced pressure to give the crude product. Flash chromatography (6% AcOEt in hexane) gives 320 mg (90%) of **28** as a white solid[42,43].

Heck coupling. Following the general procedure described above for the isomerization reaction, a mixture of neat **2** (178 mg, 1 mmol) and Ru(methylallyl)$_2$(COD) (1.5 µg, 0.0005 mol%) was heated in a 8 ml vial equipped with a magnetic stir bar at 200 °C. After cooling, iodobenzene (310 mg, 1.5 mmol), Pd(OAc)$_2$ in DMAc (0.002 mol% in 1.0 ml), Na_2CO_3 (160 mg 1.5 mmol). and TBAB (60 mg, 0.2 mmol) were added. The resulting

mixture was heated in a steel block at 125 ºC for the required time. After cooling, water was added and extracted with ethyl acetate and washed with brine. The combined organic phases were dried over $MgSO_4$, filtered and concentrated under vacuum. Flash chromatography (6% AcOEt in hexane) gives 254 mg (65%) of **29a** as a white solid[22].

Metathesis. Following the general procedure described above for the isomerization reaction, a mixture of neat **2** (356 mg, 2 mmol) and Ru(methylallyl)$_2$(COD) (3.0 µg, 0.0005 mol%) was heated in a 2 ml vial equipped with a magnetic stir bar at 200 °C. After cooling, Grubbs II pre-catalyst (1.7 mg, 0.1 mol%) was added and the mixture was heated at 90 °C. The reaction mixture became solid after 15 min. The solid was diluted in DCM, and the crude product was purified by chromatography on silica gel (6% AcOEt in hexane) to give 510 mg (85%) of **30** as a white solid[44].

Annulation. Following the general procedure described above for the isomerization reaction, a mixture of neat **2** (89 mg, 0.5 mmol) and Ru(methylallyl)$_2$(COD) (0.8 µg, 0.0005 mol%) was heated in a 8 ml vial equipped with a magnetic stir bar at 200 °C. After cooling, aniline (23 mg, 0.25 mmol), Pd(OAc)$_2$ (11 mg, 10 mol %), TsOH (17mg 0.1 mmol), water (0.25 mL) and DMSO (1 mL, 0.50 M) were added. The mixture was stirred under 1 atm of oxygen at 110 °C for 16 h. After cooling, water was added and extracted with ethyl acetate and washed with brine. The combined organic phases were dried over $MgSO_4$, filtered and concentrated under vacuum. Flash chromatography (30% AcOEt in hexane) gives 86 mg (65%) of **31** as a yellow oil[45].

Dihydroxylation. In a 50 mL round-bottomed flask equipped with magnetic stirring bar and an overpressure valve, $NaIO_4$ (642 mg, 3 mmol) was stirred in 1.5 mL H_2O. 1 M H_2SO_4 (400 µL, 0.4 mmol) was added. After all solids were dissolved the solution was cooled to 0 ºC. A 0.1 M aqueous solution of $RuCl_3$ (100 µL, 0.01 mmol) was added and the mixture was stirred until the color turned bright yellow. Ethyl acetate (6 mL) was

added and stirring was continued for 5 min. Acetonitrile (6 mL) was added and stirring was continued for further 5 min. Following the general procedure described above for the isomerization reaction, a mixture of neat **2** (356 mg, 2 mmol) and Ru(methylallyl)$_2$(COD) (3.0 μg, 0.0005 mol%) was heated in a 2 ml vial equipped with a magnetic stir bar at 200 °C. After cooling the mixture was added to round-bottomed flask with the reaction mixture and the resulting slurry was stirred for 1 h. The mixture was poured onto 15 mL sat. $NaHCO_3$ and 20 mL sat. $Na_2S_2O_3$ solution. Phases were separated and the aqueous layer was extracted with ethyl acetate. The combined organic phases were dried over $MgSO_4$, filtered and concentrated under vacuum. The crude product was purified by chromatography on a silica gel (30% AcOEt in hexane) to give 297 mg (70%) of **32** as a yellow oil[46].

Oxidation. Following the general procedure described above for the isomerization reaction, a mixture of neat **2** (89 mg, 0.5 mmol) and Ru(methylallyl)$_2$(COD) (0.8 μg, 0.0005 mol%) was heated in a 8 ml vial equipped with a magnetic stir bar at 200 °C. After cooling, MeCN (1 ml, 0.50 M), DCM (1 ml, 0.50 M), H_2O (3 ml, 0.15 M), RuO_2 (0.66 mg, 1 mol%) and $NaIO_4$ (321 mg, 1.5 mmol) were added, and the mixture was stirred at 25 ºC for 4 h. After that time, water was added and extracted with ethyl acetate and washed with brine. The combined organic phases were dried over $MgSO_4$, filtered and concentrated under vacuum. Flash chromatography (1% AcOEt in hexane) gives 75 mg (91%) of **33** as a yellow oil.

Epoxidation. Following the general procedure described above for the isomerization reaction, a mixture of neat **2** (89 mg, 0.5 mmol) and Ru(methylallyl)$_2$(COD) (0.8 μg, 0.0005 mol%) was heated in a 8 ml vial equipped with a magnetic stir bar at 200 °C. After cooling, DCM (5 ml, 0.1 M) and *m*-CPBA (104 mg, 0.6 mmol) were added, and the mixture was stirred at 25 ºC for 5 h. Then, the solvent was evaporated under reduced

pressure to give the crude product. Flash chromatography (10% AcOEt in hexane) gives 87 mg (90%) of **34** as a yellow oil.

Supplementary Discussion

- Supplementary Figures.

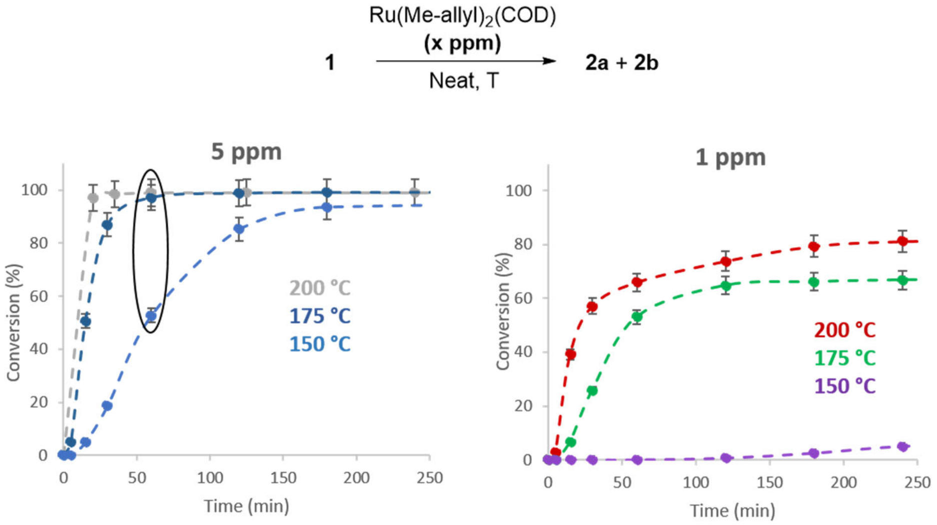

Supplementary Figure 1. Kinetics for the isomerization of methyl eugenol **1** to methyl isoeugenol **2** catalyzed by 5 ppm (left) and 1 ppm (right) of Ru(methyallyl)2(COD) at different reaction temperatures.

Entry	Catalyst	Price (€/g)
1	$Ru(Me\text{-}allyl)_2(COD)$	129
2	$RuCl_2(PPh_3)_3$	41
3	$Ru(CO)H_2(PPh_3)_3$	169
4	Milstein	684
5	Ru-PNN (Gusev)	724
6	$RuCl_2(C_{14}H_{32}NP)_2$	241
7	Grubbs 1st generation	92
8	Grubbs 2nd generation	217
9	Hoveyda-Grubbs 2nd generation	265
10	$Ru_3(CO)_{12}$	143

Supplementary Figure 2. Prices for soluble Ru catalysts, taken from the Merck Aldrich Co. catalogue.

Catalyst	Price Merck-Aldrich (€/g)	Molecular Weight (g/mol)	wt% metal	Price per metal (€/g)
$Ru(Me\text{-}allyl)_2(COD)$	129	319.45	31	416
$RuCl_2(PPh_3)_3$	41	958.83	10	410
$Ru_3(CO)_{12}$	143	639.33	47	304
$RhCl_3$	230	209.26	48	479

Supplementary Figure 3. Prices for Ru and Rh catalysts, taken from the Merck Aldrich Co. catalogue.

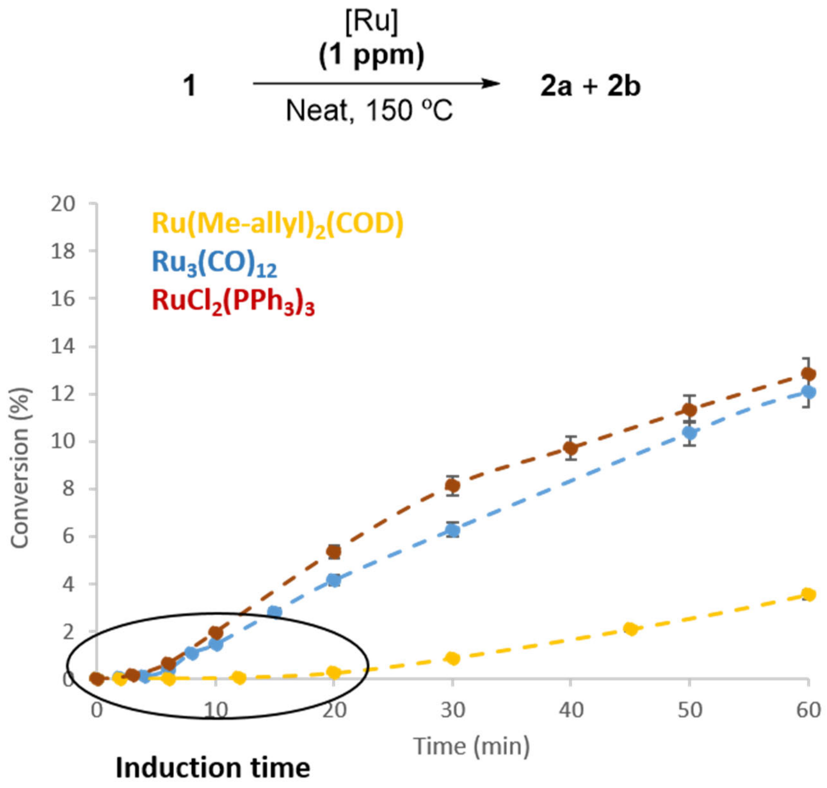

Supplementary Figure 4. Kinetics for the isomerization of methyl eugenol **1** to methyl isoeugenol **2** catalyzed by 1 ppm of different Ru complexes at 150 ºC.

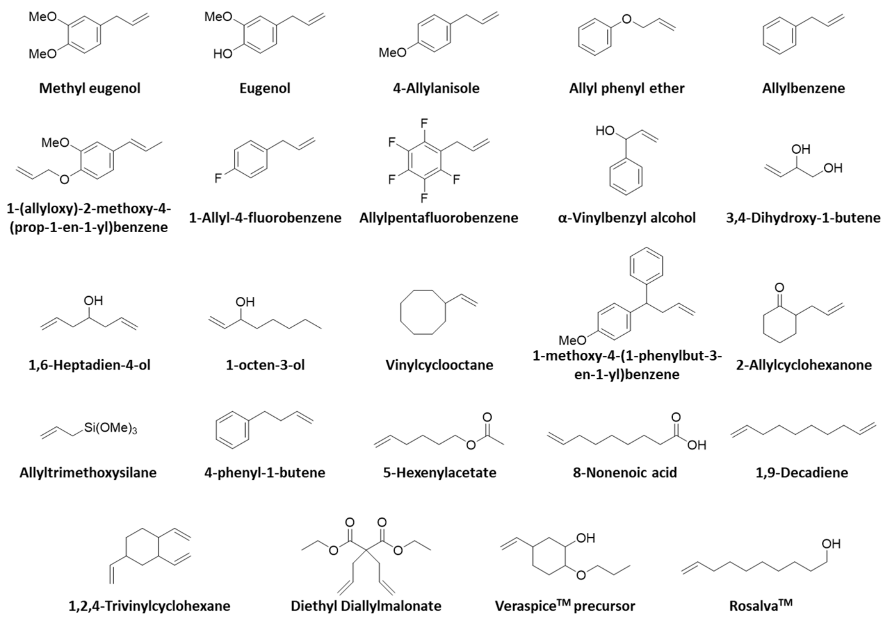

Supplementary Figure 5. Structures of the different starting materials employed in this study.

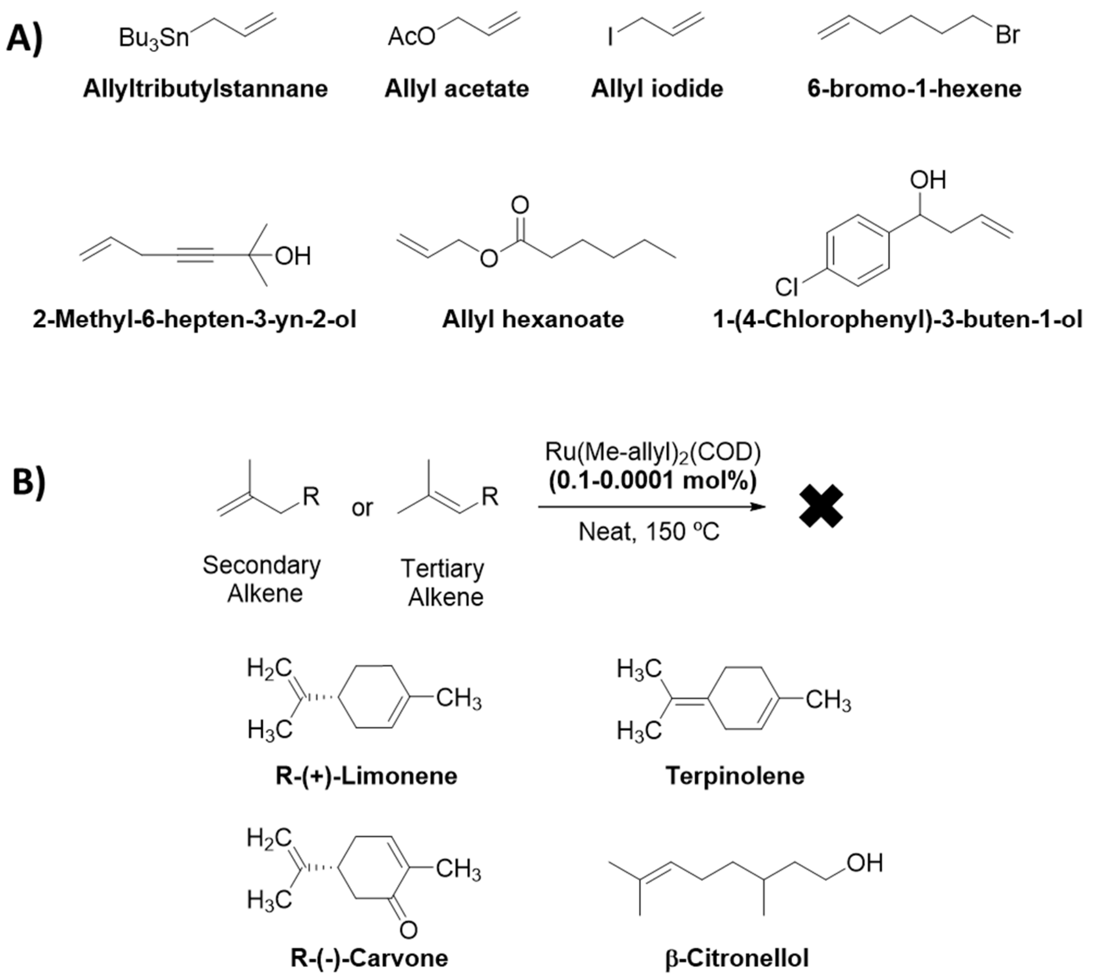

Supplementary Figure 6. A) Unreactive terminal alkenes using Ru(methyallyl)$_2$(COD) (0.2-0.005 mol%) at 150 ºC. B) Unreactive substituted alkenes under the Ru-catalyzed reaction conditions reported here.

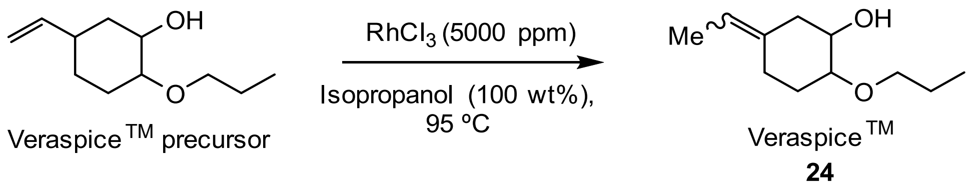

Supplementary Figure 7. Reaction conditions for the industrial synthesis of Veraspice^{TM}.

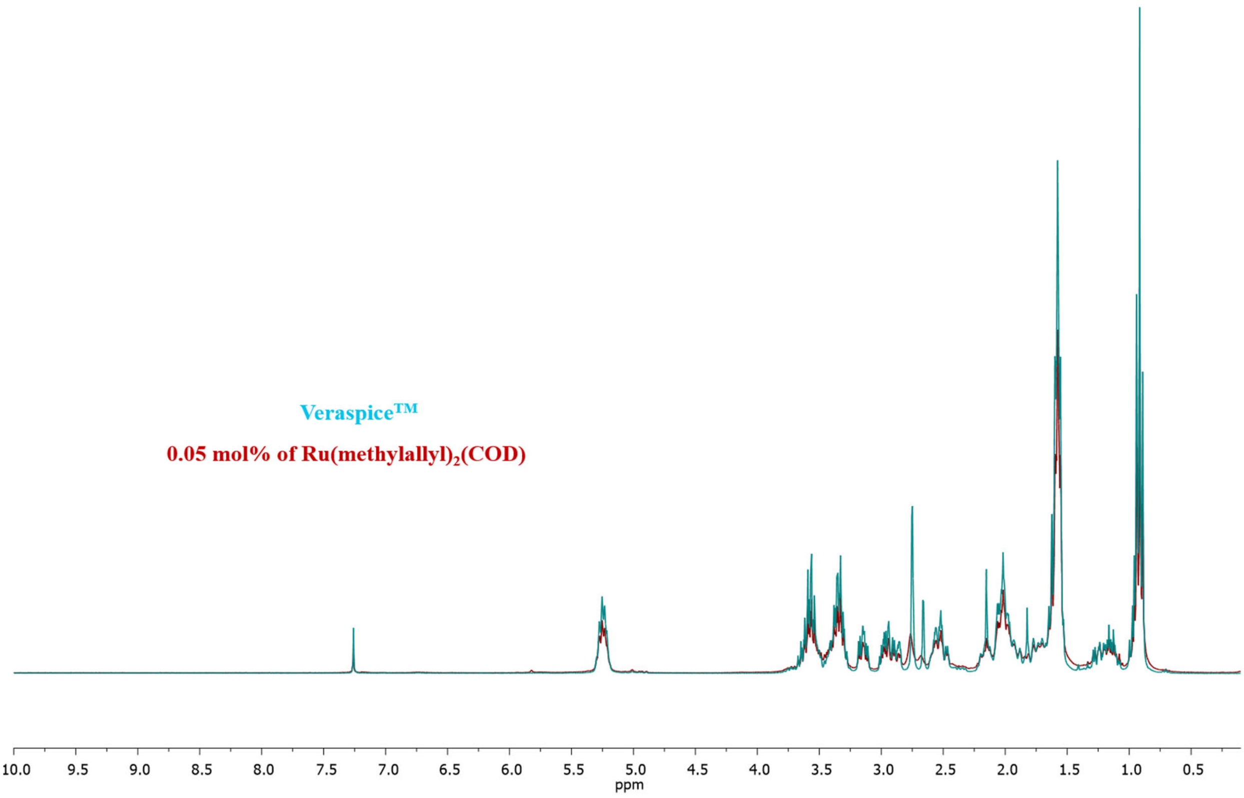

Supplementary Figure 8. ^{1}H-NMR spectrum of Veraspice^{TM} (light blue line) compared to the isomerization product **24** produced with 500 ppm of the Ru catalyst (red line) after 3 h reaction time.

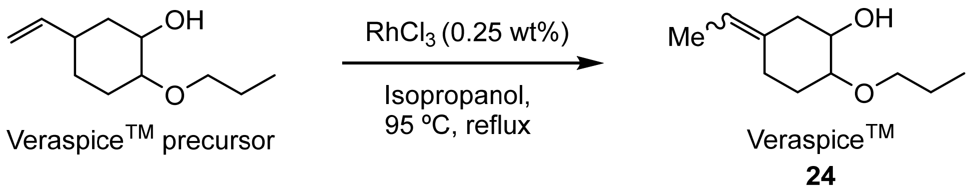

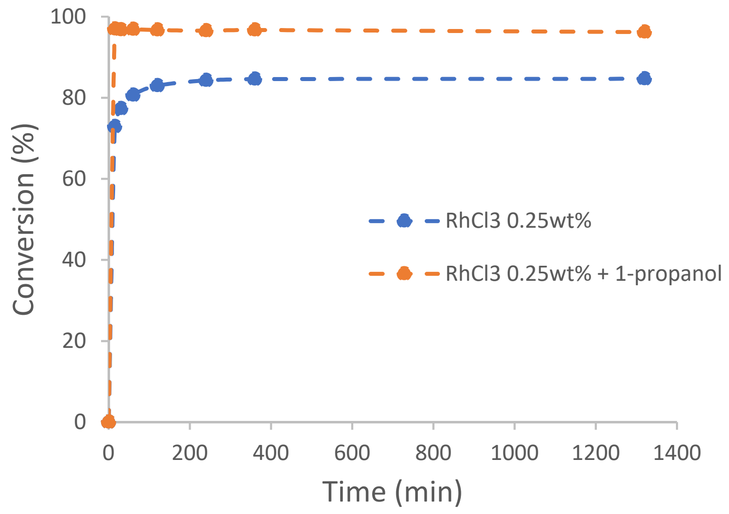

Supplementary Figure 9. Kinetic plots for the RhCl3-catalyzed isomerization of the Veraspice™ precursor with or without propanol in the reaction mixture.

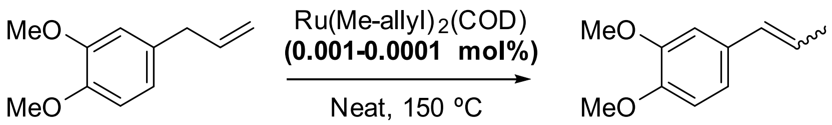

Methyl eugenol **1**

Methyl isoeugenol **2**

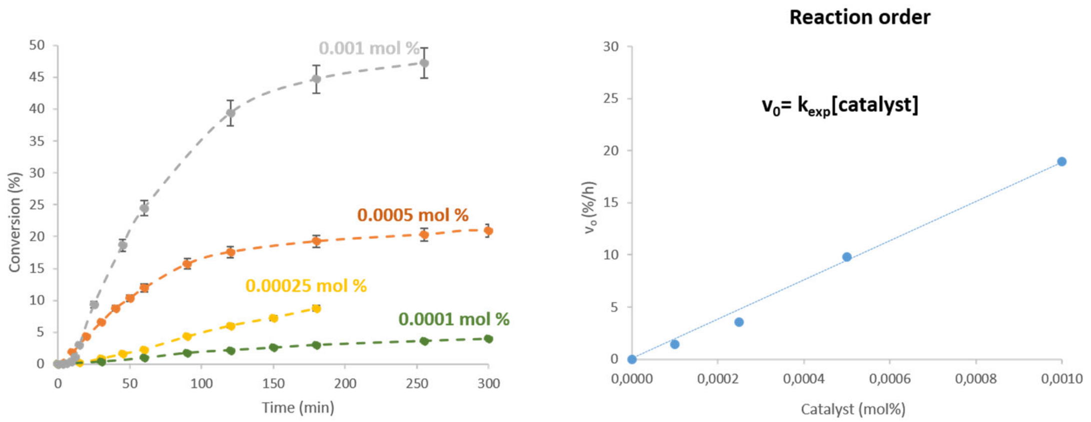

Supplementary Figure 10. Reaction orders for the isomerization of methyl eugenol **1** to methyl isoeugenol **2** catalyzed by 1-10 ppm of Ru(methyallyl)$_2$(COD) at 150 ºC. Error bars represent a 5% uncertaintity.

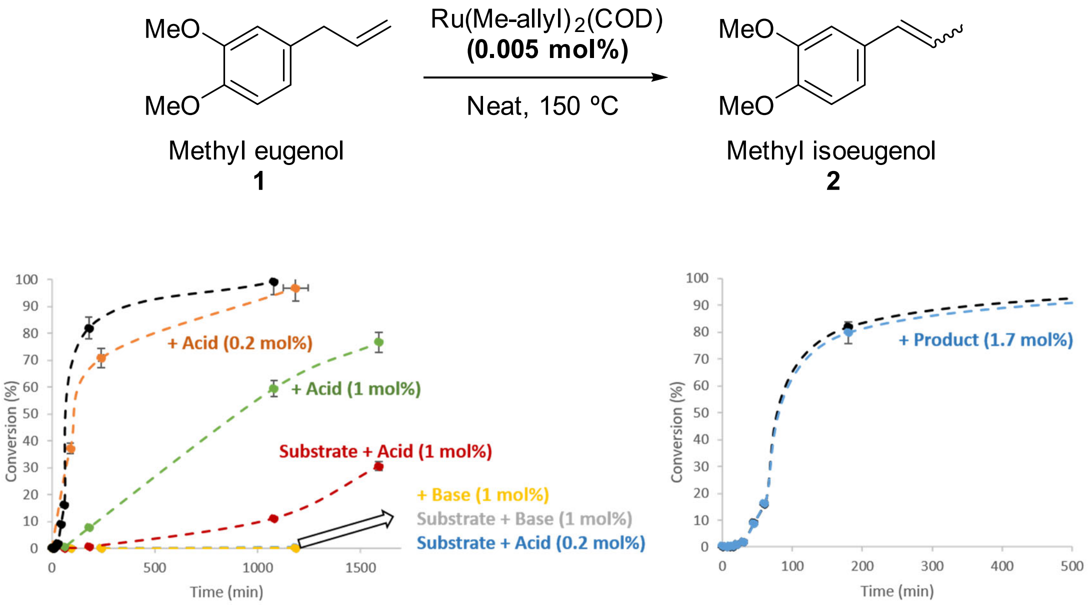

Supplementary Figure 11. Influence in the isomerization reaction of the addition of HCl gas (acid), DBU (base), product **2** and substrate **1** during reaction, for the isomerization of methyl eugenol **1** to methyl isoeugenol **2** catalyzed by 50 ppm of Ru(methyallyl)2(COD) at 150 ºC. Error bars represent a 5% uncertaintity. For orange, green and yellow line, the reaction was carried out with the catalyst and acid or base. For red, gray, blue line, reaction was carried out with acid or base but without catalyst.

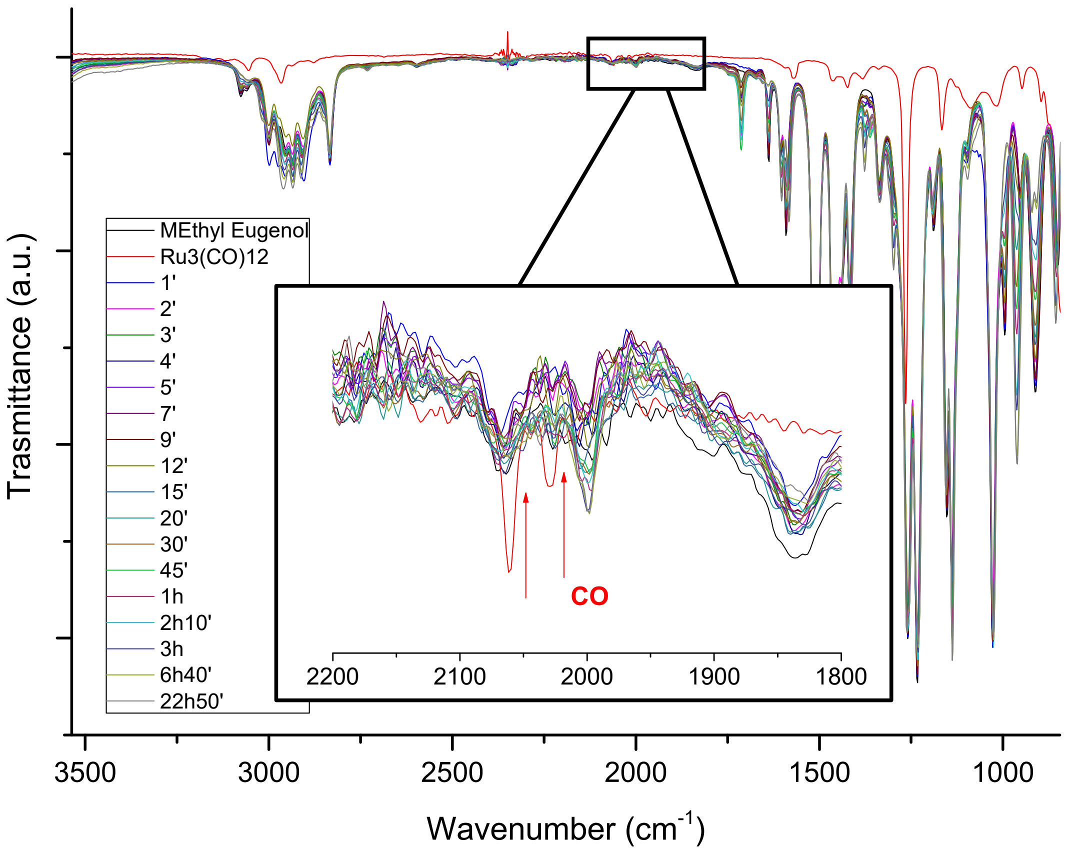

Supplementary Figure 12. FT-IR spectra for the isomerization of methyl eugenol **1** (black line) to methyl isoeugenol **2** catalyzed by 300 ppm of $Ru_3(CO)_{12}$ (red line) at 150 ºC and different reaction times. The inset shows the diagnostic area, where the complete disappearance of the CO peaks from the beginning of the reaction is observed, although somewhat blurred by starting material alkene traces.

Comments: The determination of the active Ru species for the isomerization reaction was difficult due to the tiny amounts of metal employed, which is below the detection limit of many techniques, however, some measurements with high sensibility towards the metal, either soluble or supported, were informative. For instance, the isomerization of **1** to **2** catalyzed by $Ru_3(CO)_{12}$ was followed by Fourier–transformed infrared spectroscopy (FTIR, Fig. S12) and the peak associated to CO seemingly disappears from the beginning of the reaction, despite impurities of alkene **1** partially hide the peak. Besides, it was found that the Ru catalyst (10 ppm) co–distils with alkene product **24** at kg scale, which supports the formation of an alkene–Ru complex after total exchange of the CO ligands.

With $Ru_3(CO)_{12}$:

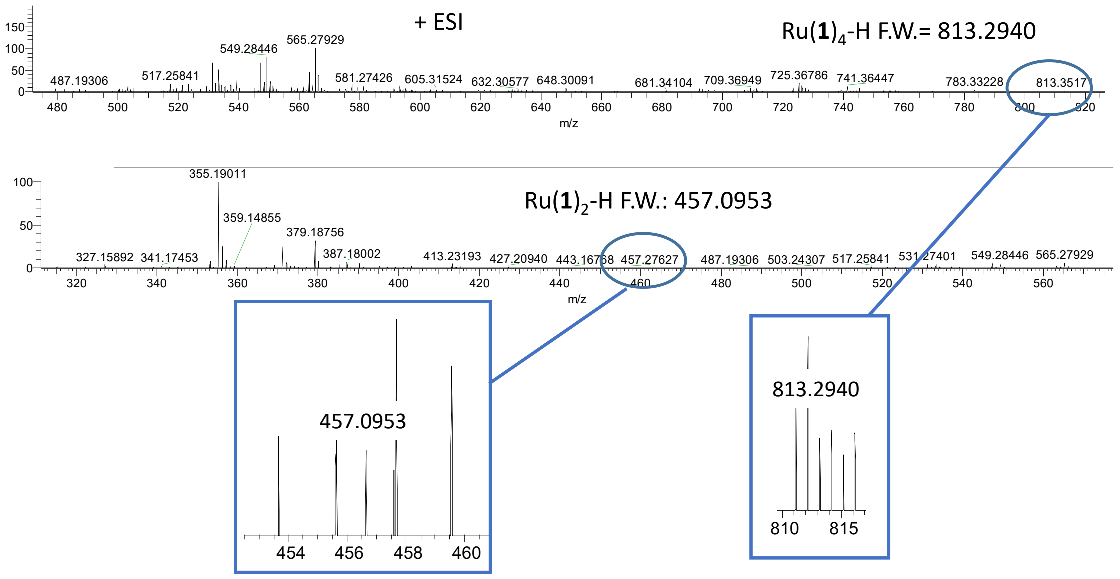

With $Ru(Me\text{-}allyl)_2(COD)$:

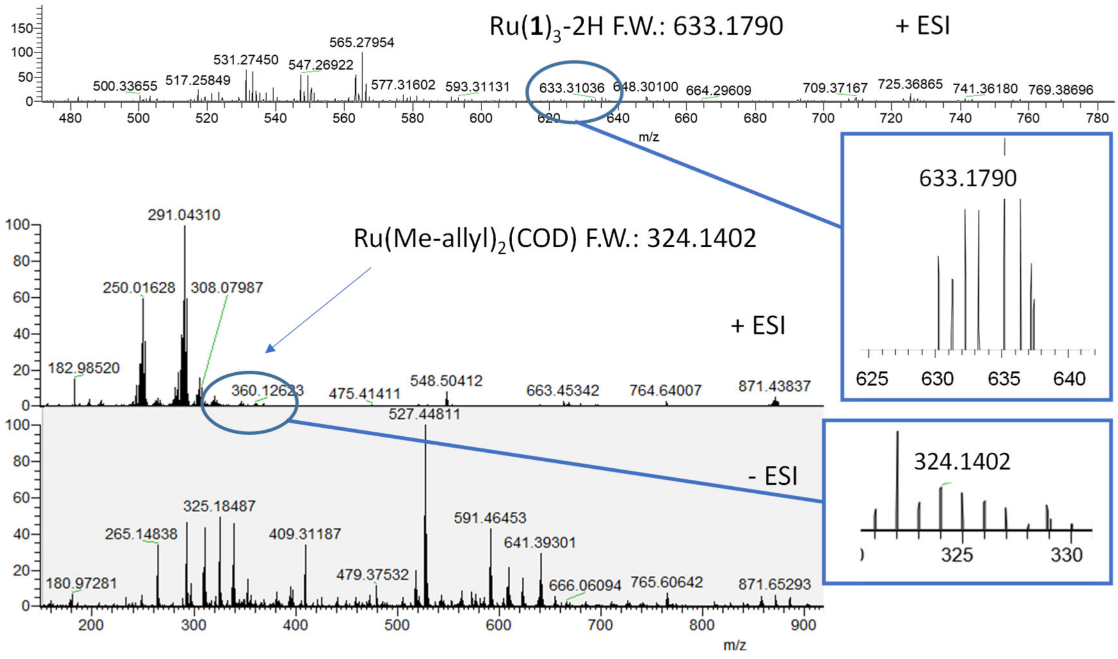

Supplementary Figure 13. Mass spectrometry results for the Ru-catalyzed isomerization of methyl eugenol **1** to methyl isoeugenol **2** catalyzed by 300 ppm of $Ru_3(CO)_{12}$ (top) or $Ru(methyallyl)_2(COD)$ (bottom), at 150 ºC. The spectra below show the results for the neat Ru complex, in both positive and negative modes, and the peaks corresponding to the initial Ru complex are not present in the final mixture (not shown).

Comments: To visualize this Ru–alkene complex, an Orbitrap analyzer with flow injection–mass spectrometry (HPLC–Orbitrap MS) was employed. This instrumentation is capable of determining parts–per–billion of organic analytes, but has rarely employed

for organometallic compounds. The results here with the Ru–catalyzed isomerized mixture (Fig. S13), for two different Ru catalysts, show the formation of a Ru(**1**)$_4$ complex.

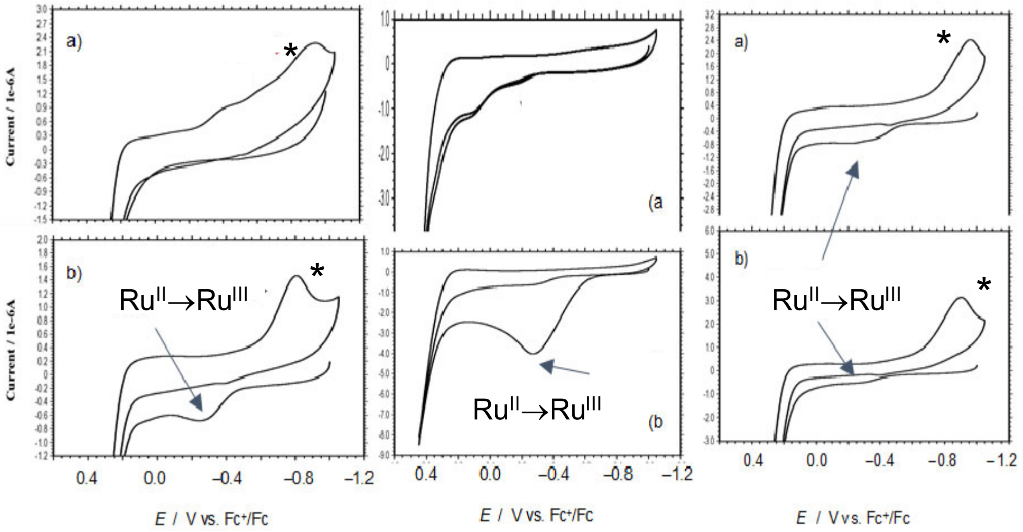

Supplementary Figure 14. Cyclic voltammetry at glassy carbon electrode for the isomerization of **1** to **2** in 1:1 v/:v solutions of 100 ppm of $RuCl_3$, Ru(methylallyl)$_2$(COD) and $Ru_3(CO)_{12}$, in **1** plus 0.10 M Hex_4NPF_6/MeCN. Potential scan initiated at -1.0 V vs. Fc^+/Fc in the positive direction; potential scan rate 50 mV s^{-1}. The Figure compares the voltammograms before (a, upper file) and after reaction (b, lower file). The more informative signals are marked with arrows. Peaks marked with * denote the reduction of Ru^{II}-MeCN species generated during previous electrochemical processes. Notice that the experimental conditions are reasonable comparable with the standard reaction conditions here reported.

Comments: To assess the oxidation state of the Ru catalyst during the isomerization of **1** to **2**, Figure S14 shows cyclic voltammetry measurements with different Ru catalysts, and the results clearly unveil that, regardless the initial oxidation state of the metal, Ru^{II} to Ru^{III} oxidation signals are the prevalent peaks in the final mixture. Upon starting the potential at -1.0 V vs. Fc^+/Fc in the positive direction, $RuCl_3$ shows no oxidation signals but display three overlapping reduction peaks between -0.4 – -1.0 V in the subsequent cathodic scan. These signals can be attributed to the stepwise reduction of the Ru^{III} complex to analogue Ru^{II} and Ru^0 complexes accompanied by the partial complex dissociation and formation of $Ru^{II}(MeCN)_n$ complexes in turn reduced to Ru^0 (see Supplementary information). The formation of such complexes[43] and the participation of MeCN in the coordination sphere of much Ru^{II} complexes has been extensively documented,[44] only being displaced in the presence of strong complexing agents. In the case of the Ru(methylallyl)$_2$(COD) complex, the voltammogram consists of two overlapping anodic waves between 0.0 and 0.4 V with no cathodic counterparts. These

signals can be attributed to the apparently irreversible Ru^{II} to Ru^{III} oxidation, presumably involving some MeCN-coordinated form. The CV of the $Ru_3(CO)_{12}$ complex presents an ill-defined anodic wave at ca. -0.2 V preceding a prominent anodic current ca. 0.5 V. As a result, in the subsequent negative-going potential scan, a cathodic peak appears at -0.6 V. This signal, quite similar to the third cathodic wave recorded for the Ru^{III} complex, can be assigned to the reduction of the $Ru^{II}(MeCN)_n$ species previously generated in the anodic scan. In all cases, however, after reaction with isoeugenol, the voltammograms collapse to a quite similar profile consisting of a unique, well-defined anodic wave at -0.30 V in the initial anodic scan. These signals are a blueprint of Ru^{II} species, as observed for $Ru(methylallyl)_2(COD)$ and their common appearance suggests the formation of a common Ru^{II} *per*–alkene complex[45] regardless the initial Ru source, either by oxidation of Ru^0 or reduction of Ru^{III} under the heating reaction conditions.

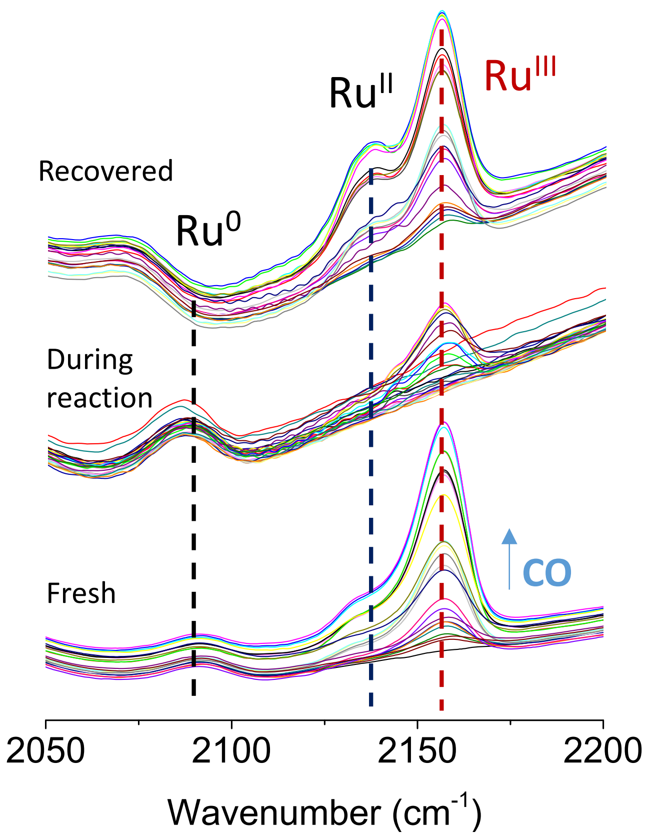

Ru^III
Ru^II
Recovered
Ru^0
During reaction
Fresh
CO
2050
2100
2150
2200
Wavenumber (cm-1)

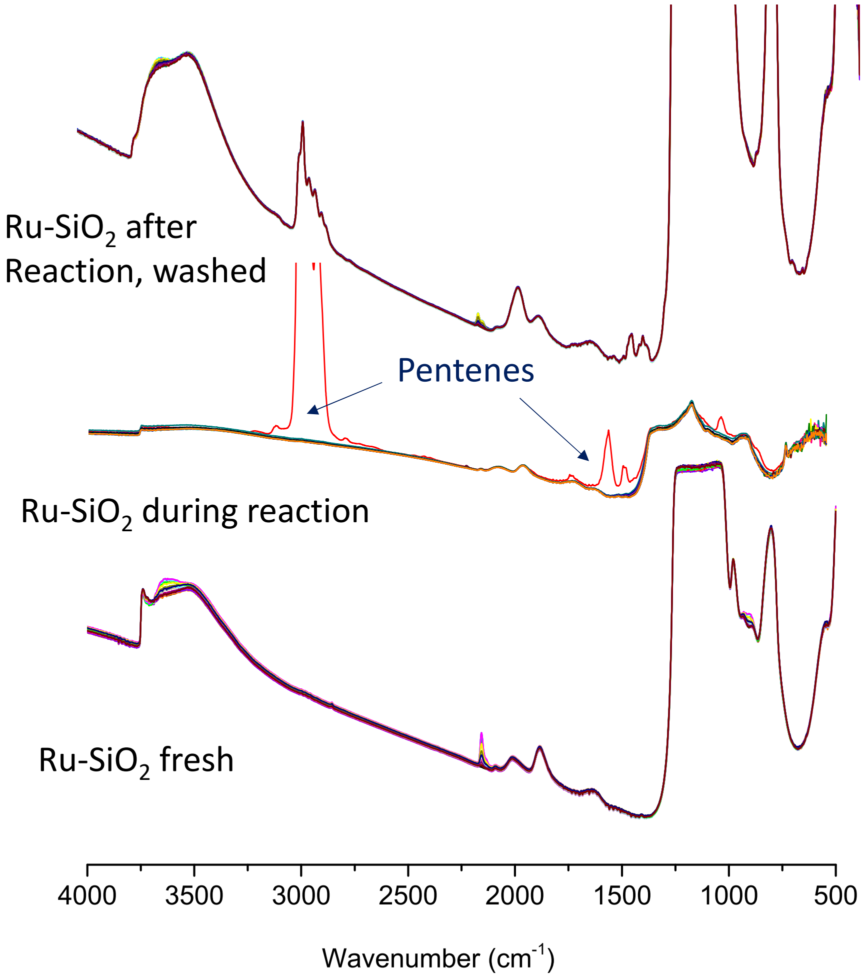

Ru-SiO2 after Reaction, washed
Pentenes
Ru-SiO2 during reaction
Ru-SiO2 fresh
4000
3500
3000
2500
2000
1500
1000
500
Wavenumber (cm-1)

Supplementary Figure 15. Top: CO–probe Fourier–transformed infrared spectroscopy at -196 ºC of a fresh sample of $RuCl_3$ impregnated on silica (FTIR–CO Ru–SiO_2, bottom), then heating at 150 ºC in a stream of 1–pentene, evacuating and cooling back to -196 ºC (middle), and after recovering the solid catalyst of a batch reaction. Bottom: CO-probe FT-IR spectra at -196 ºC of Ru-SiO_2 before, during and after the isomerization of 1-pentene to 2- and 3-pentenes in the IR chamber at 150 ºC for 1 h. Notice that the experimental conditions are reasonable comparable with the standard reaction conditions here reported.

Comments: The similar catalytic results observed above for soluble and supported Ru suggests that the Ru–supported solid catalyst can also be employed to unveil the true Ru catalytic species. Figure S15 shows the HF_1 region (2050–2200 cm^{-1}) of the low–temperature CO–probe FTIR spectrum of $RuCl_3$ impregnated on silica (Ru–SiO_2), carried out after performing the Ru–catalyzed isomerization of 1–pentene in the IR chamber, in order to avoid any contact with the atmosphere. Ru–SiO_2 was selected for this study because it is known that Ru is present on surface in a variety of oxidation states, from Ru^{III} to Ru^0, and the HF_1 region contains diagnostic signals for all these oxidation states[46]. The results show that the Ru^{n+} sites at 2134 cm^{-1} do not admit CO after the isomerization reaction, without exposition to the atmosphere, since the alkenes are strongly attached to this site. In contrast, other Ru^{n+} sites (2090 and 2057 cm^{-1}) re–admit CO just after reaction, which indicates that the alkenes are barely coordinated. If the sample is then exposed to the atmosphere and washed, as in a regular batch procedure, the Ru^{n+} sites at 2134 cm^{-1} re–appear and even increase its relative number to the other metal sites. Despite the exact interpretation of a Ru–CO FT–IR spectrum is not conclusive with different CO–coordinated species co–existing, one can say in a first approximation that the Ru^{n+} sites at 2134 cm^{-1} are labile during the catalysis, and since these sites have been previously assigned to species compatible with Ru^{II}, while the inactive Ru sites at 2090 and 2057 cm^{-1} have been assigned to Ru^0 and $Ru^{I–III}$, respectively[47], Ru^{II} seems the active Ru oxidation state.

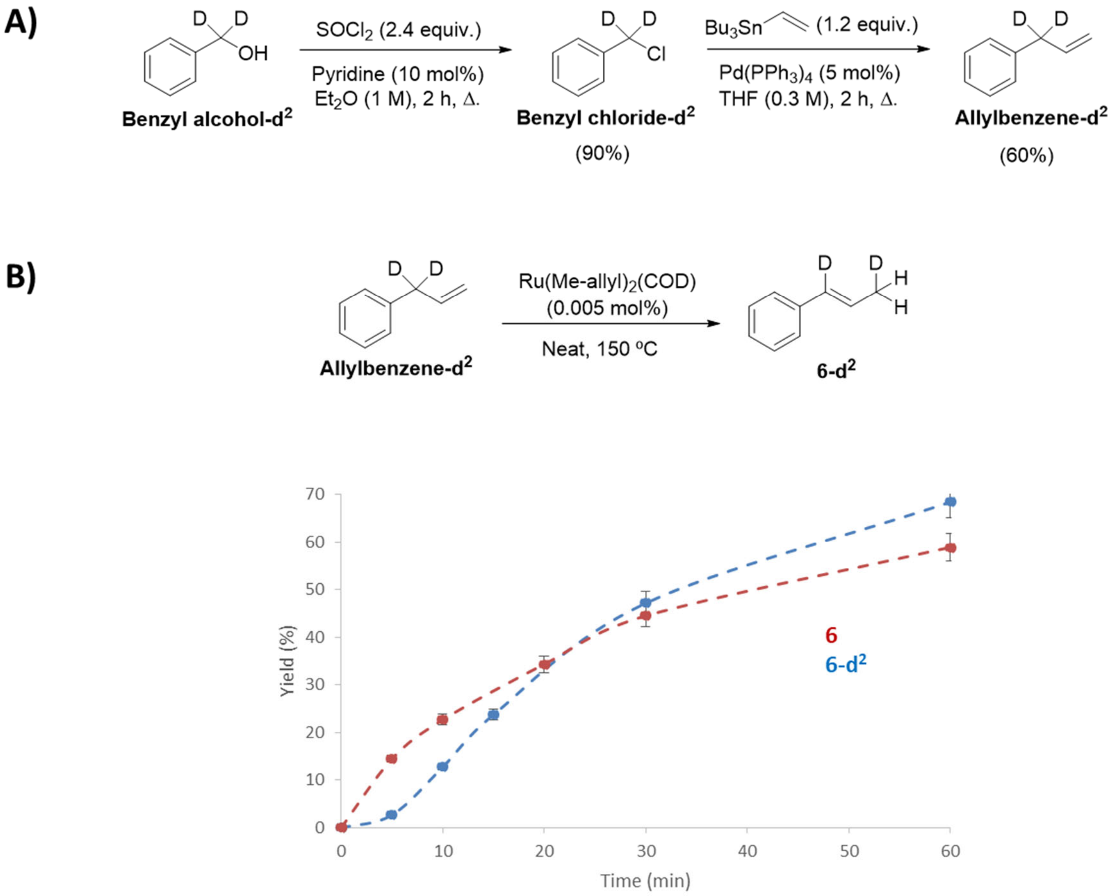

Supplementary Figure 16. A) Synthesis of deutered **allylbenzene-*d*^{2}**. B) Kinetic experiments with **allylbenzene** and deuterated **allylbenzene-*d*^{2}**. Error bars represent a 5% uncertaintity.

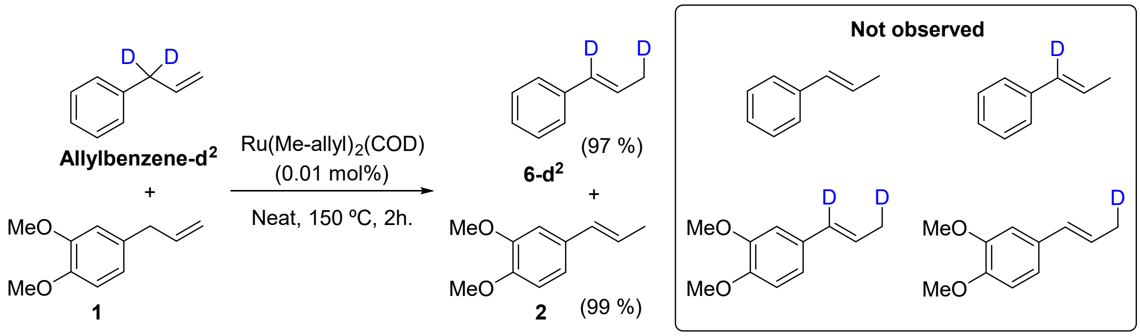

Supplementary Figure 17. Kinetic experiments with deuterated **allylbenzene-d^2** and methyl eugenol **1,** to check the possible scrambling between hydrogens.

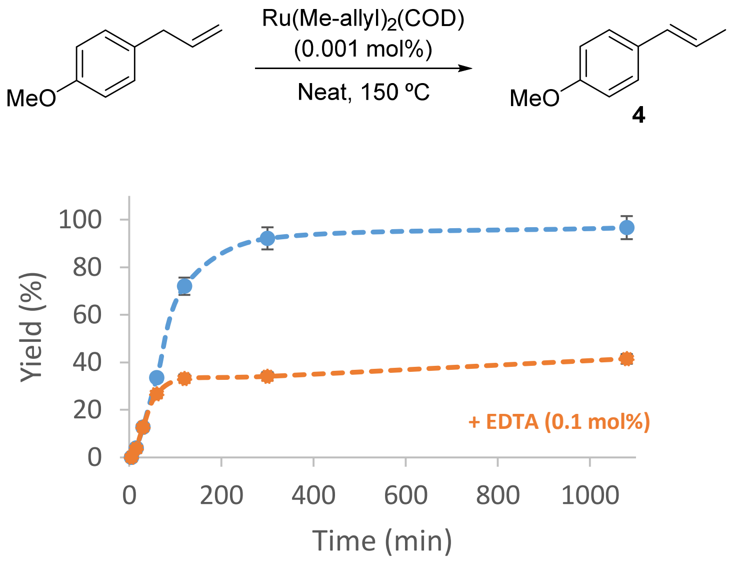

Supplementary Figure 18. Kinetics for the isomerization reaction with (orange line) or without (blue line) EDTA. The use of CCl_4 and TEMPO as additives instead of EDTA did not produce any decrease of the catalytic acitivity.

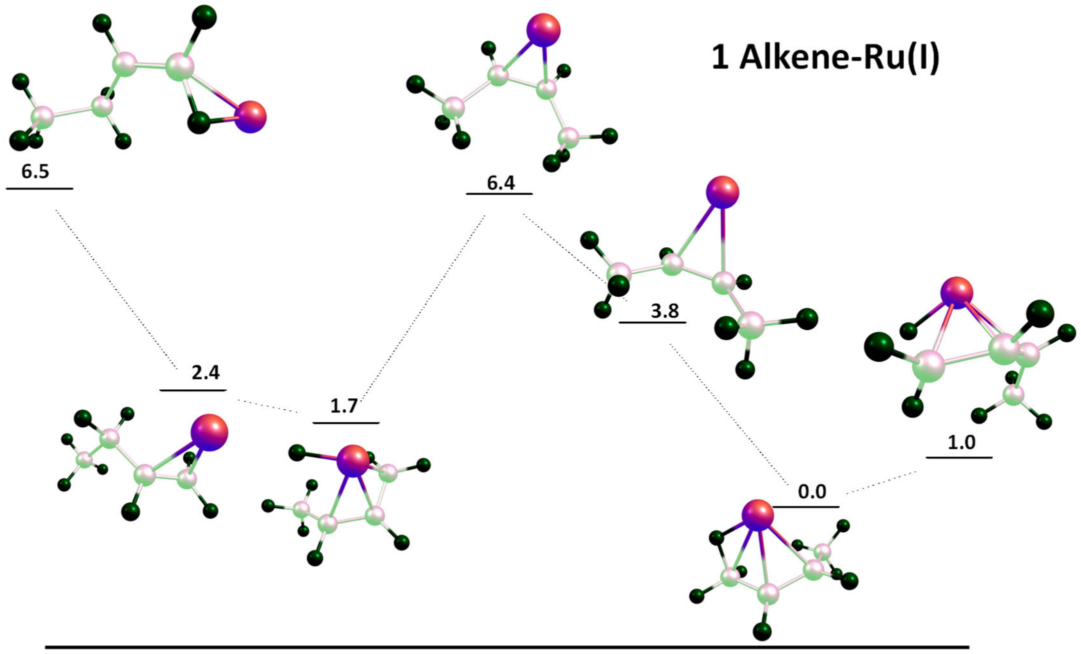

1 Alkene-Ru(II)

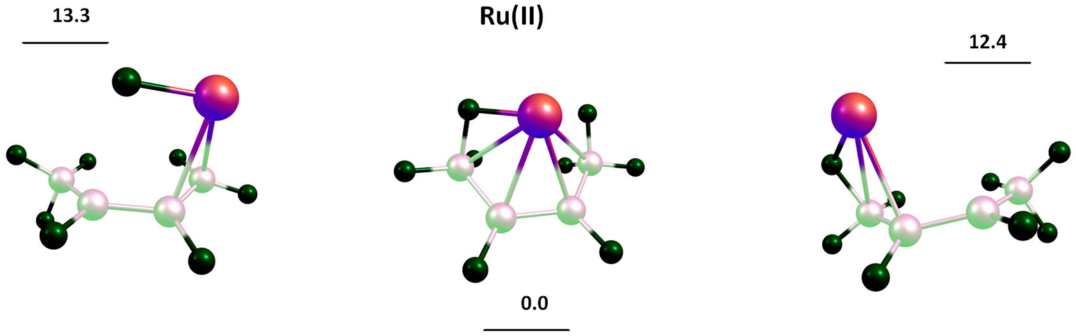

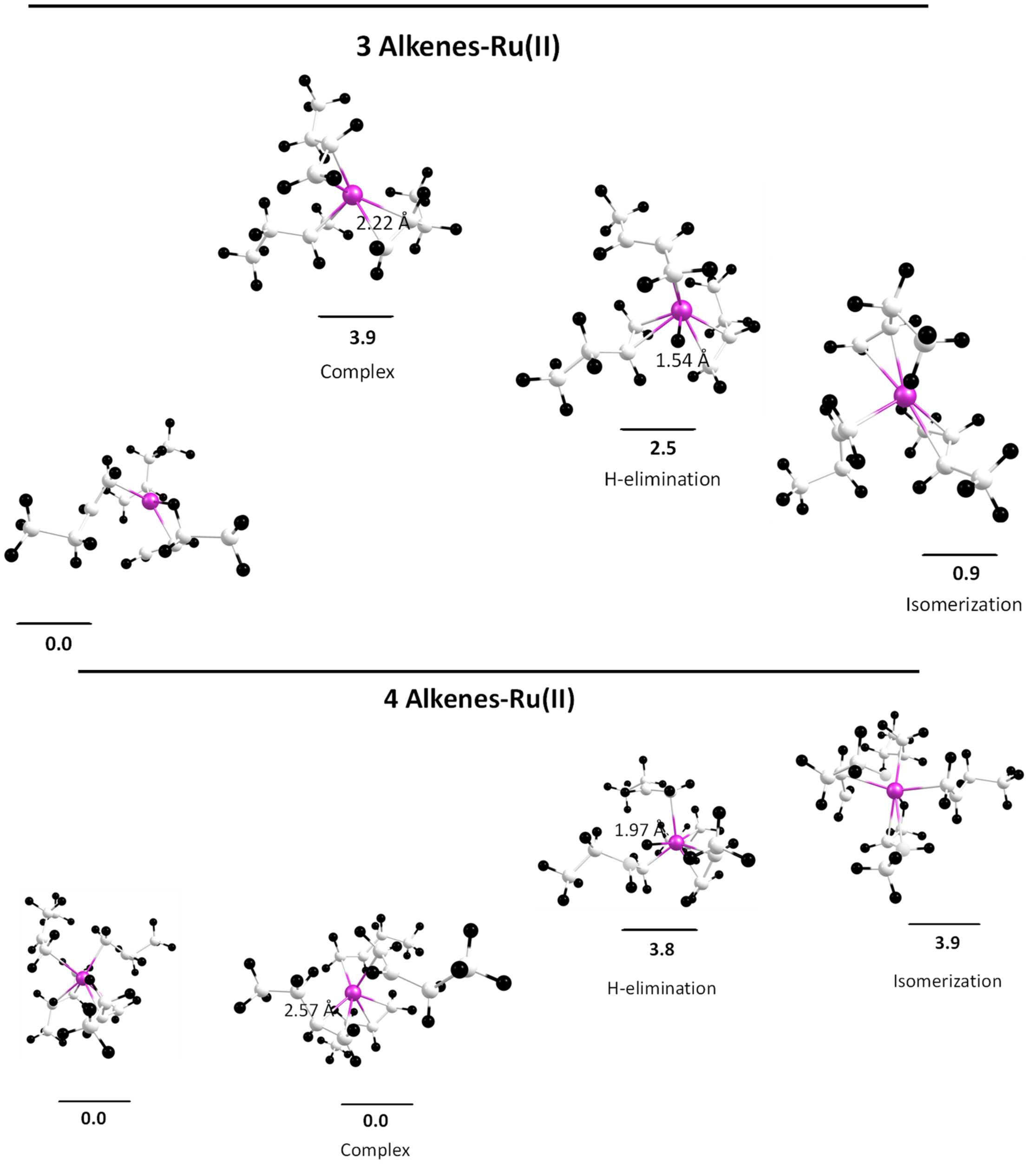

Supplementary Figure 19. Density functional theory (DFT) calculations for the Ru-catalyzed isomerization of 1-butene. From top to bottom: Ru(I) with one alkene molecule, Ru(II) with one alkene molecule, Ru(II) with three alkene molecules and Ru(II) with four alkene molecules. The energetic differences are expressed in kcal·mol^{-1}. Selected Ru-C and Ru-H bond distances are shown. Code color: purple, while and black balls correspond to Ru, C and H atoms, respectively.

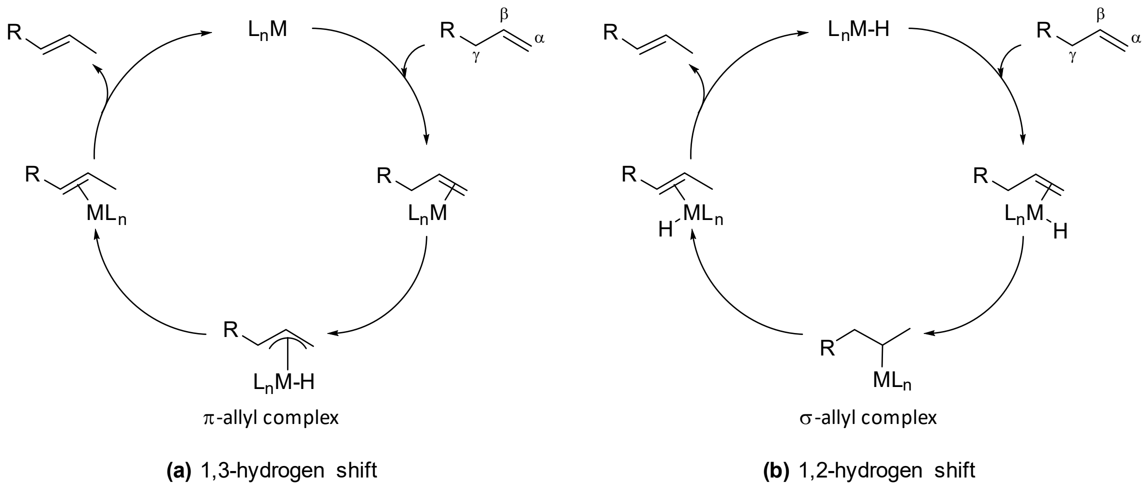

Supplementary Figure 20. Comparison of the two most prevalent alkene isomerization catalytic cycles: (a) π-allyl mechanism (1,3–hydrogen shift), (b) σ-alkyl mechanism (1,2–hydrogen shift). The plausible mechanism here is (a).

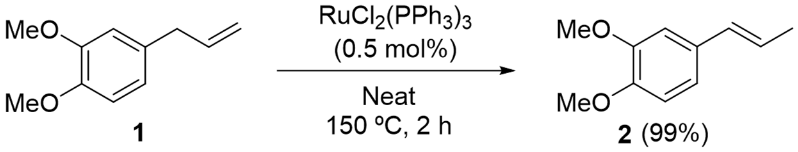

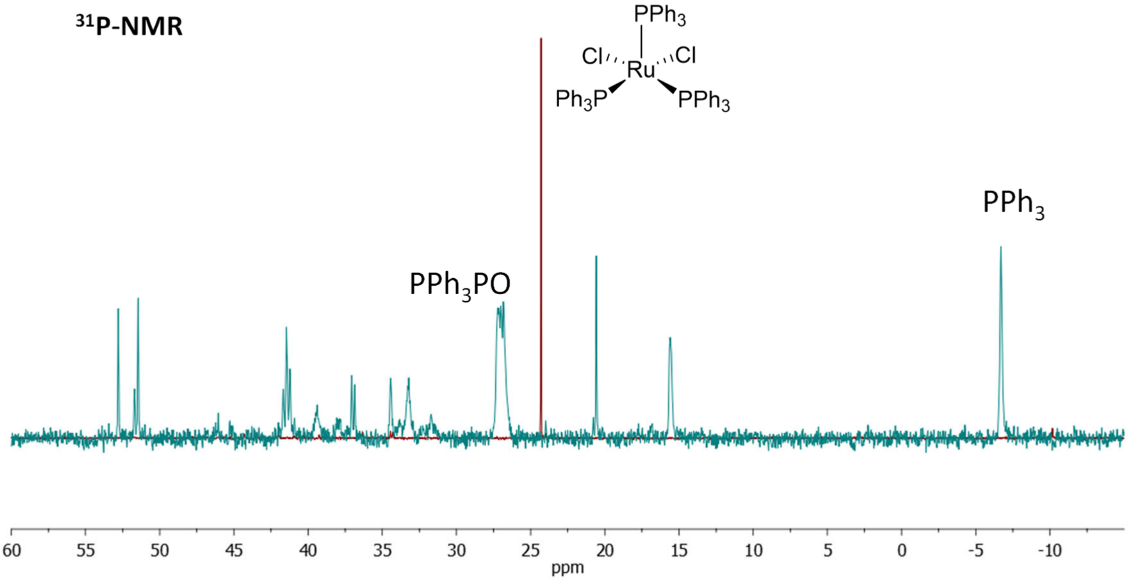

Supplementary Figure 21. ^{31}P-NMR spectra of the starting $RuCl_2(PPh)_3$ complex before (red line) and after 2 h reaction time (blue line) of the isomerization reaction of **1** under the indicated reaction conditions.

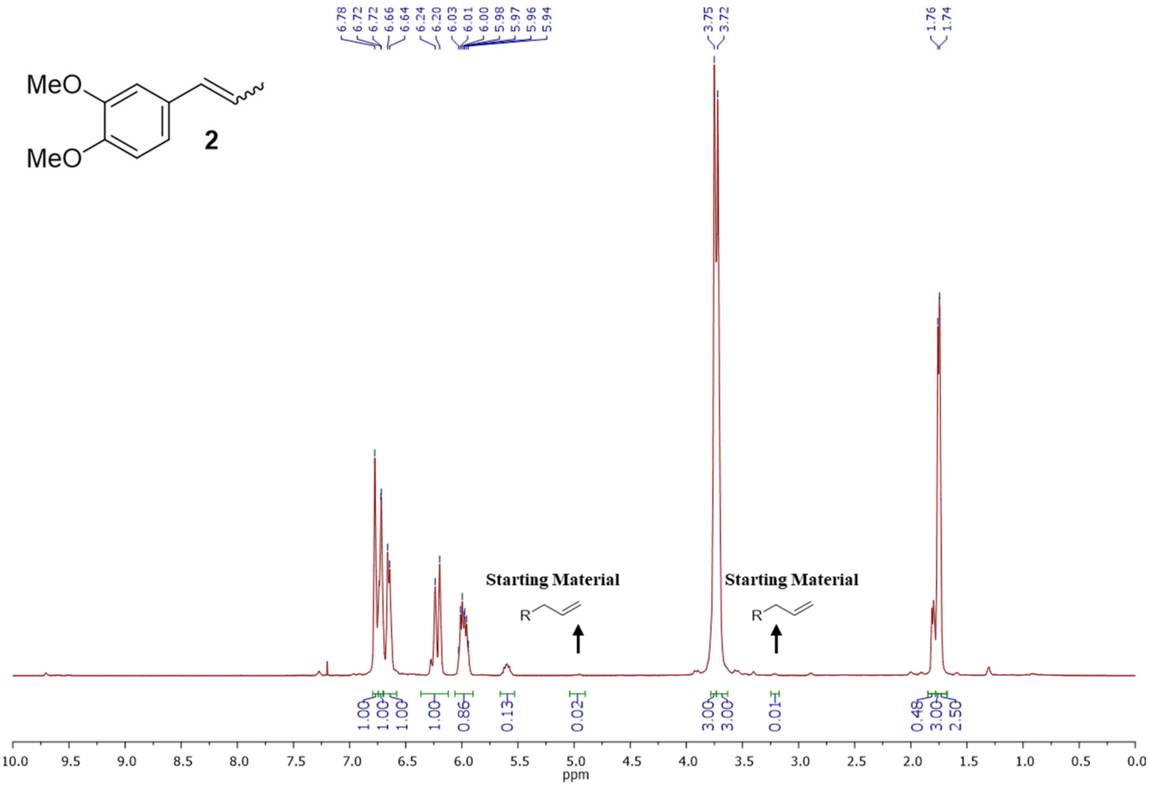

Supplementary Figure 22. ^{1}H-NMR of compound **2**, recorded at 400 MHz and 25 ºC in $CDCl_3$.

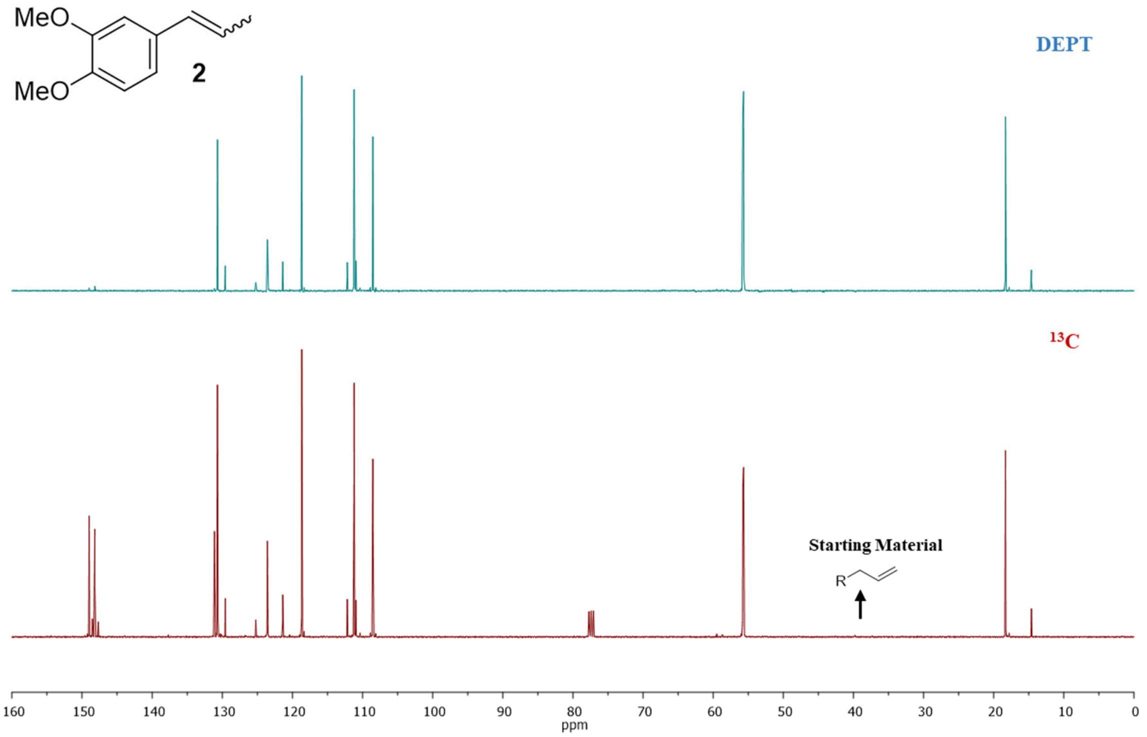

Supplementary Figure 23. ^{13}C-NMR and DEPT of compound **2**, recorded at 100 MHz and 25 ºC in $CDCl_3$.

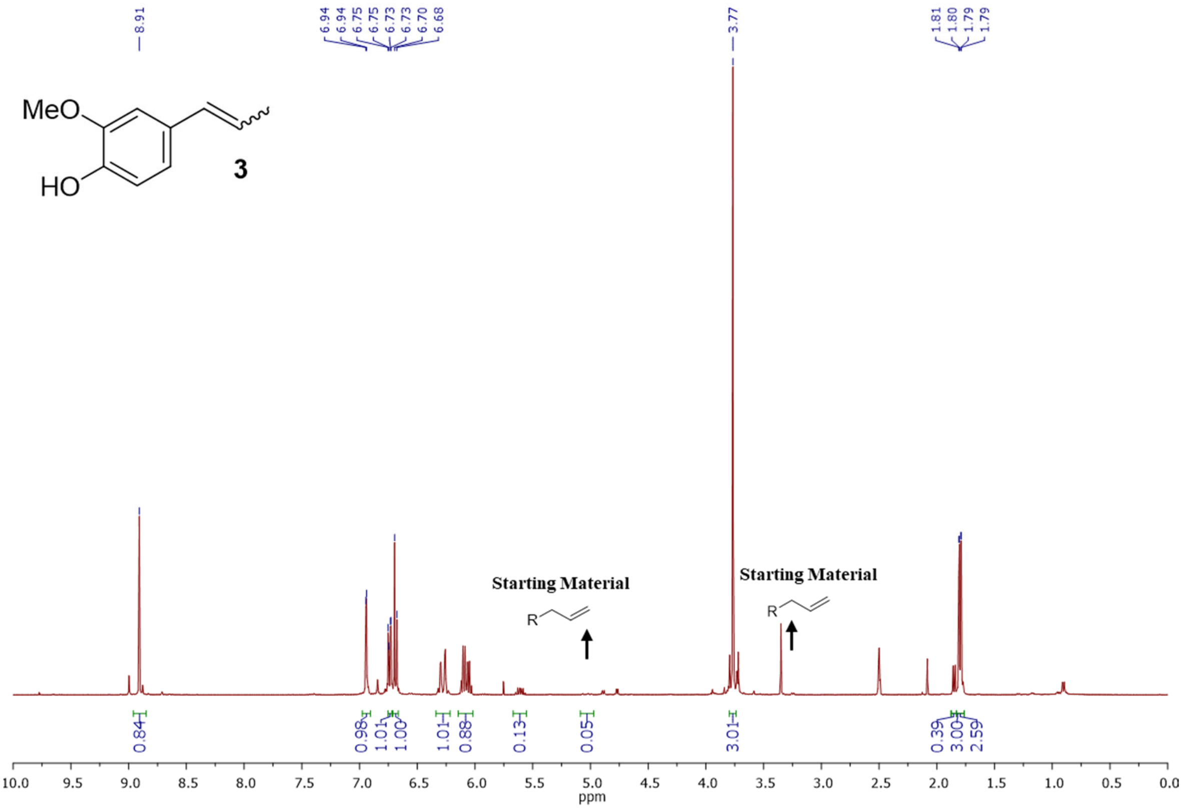

Supplementary Figure 24. ^{1}H-NMR of compound **3**, recorded at 400 MHz and 25 ºC in $CDCl_3$.

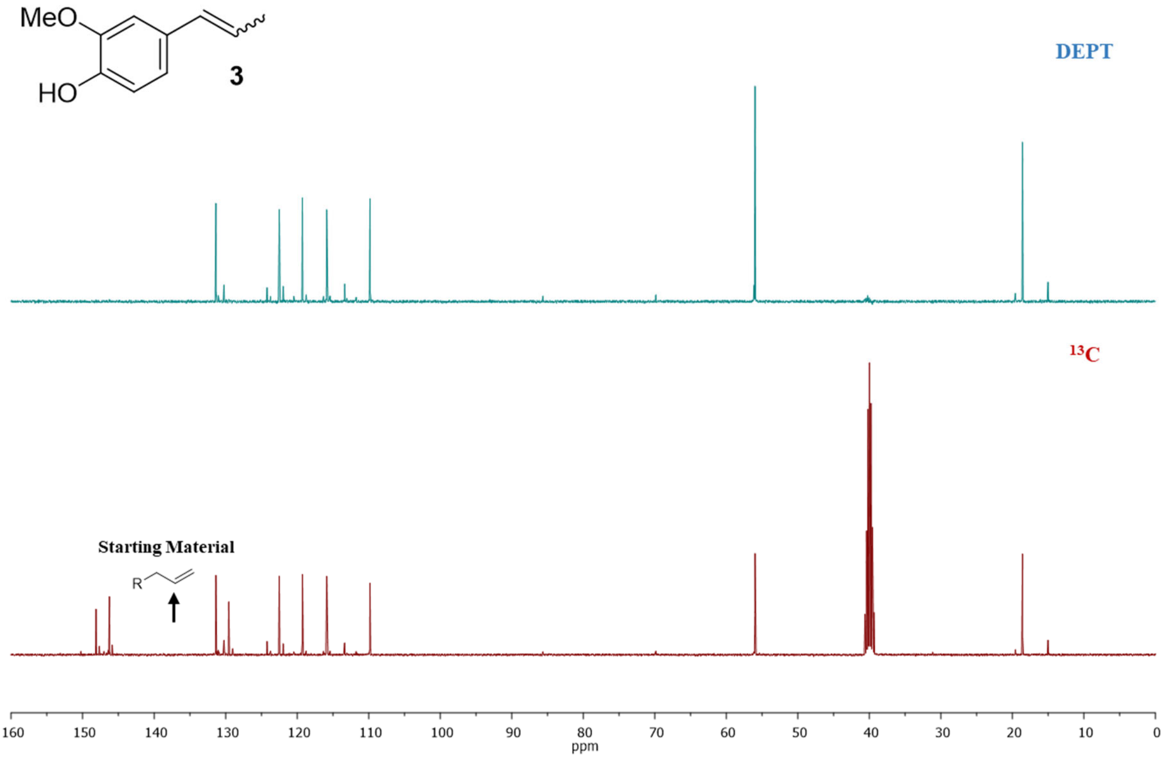

Supplementary Figure 25. ^{13}C-NMR and DEPT of compound **3**, recorded at 100 MHz and 25 ºC in $CDCl_3$.

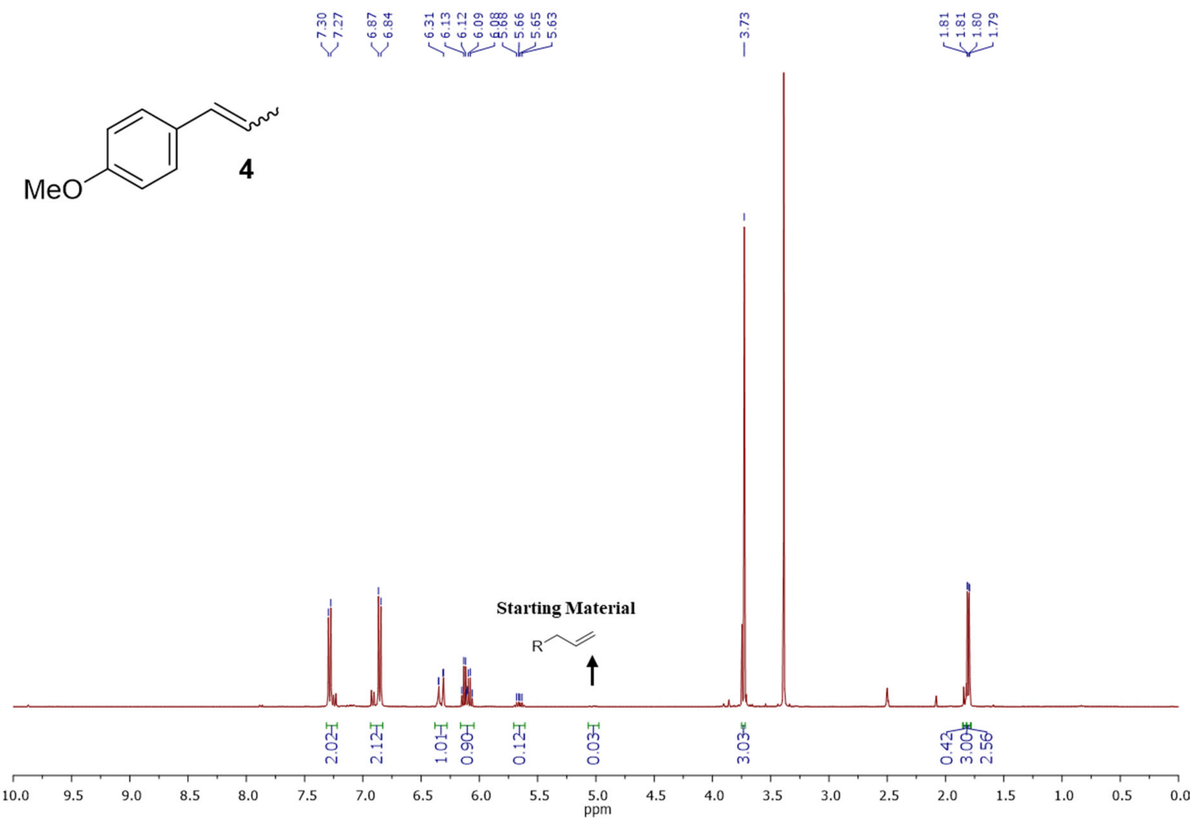

Supplementary Figure 26. ^{1}H-NMR of compound **4**, recorded at 400 MHz and 25 ºC in $CDCl_3$.

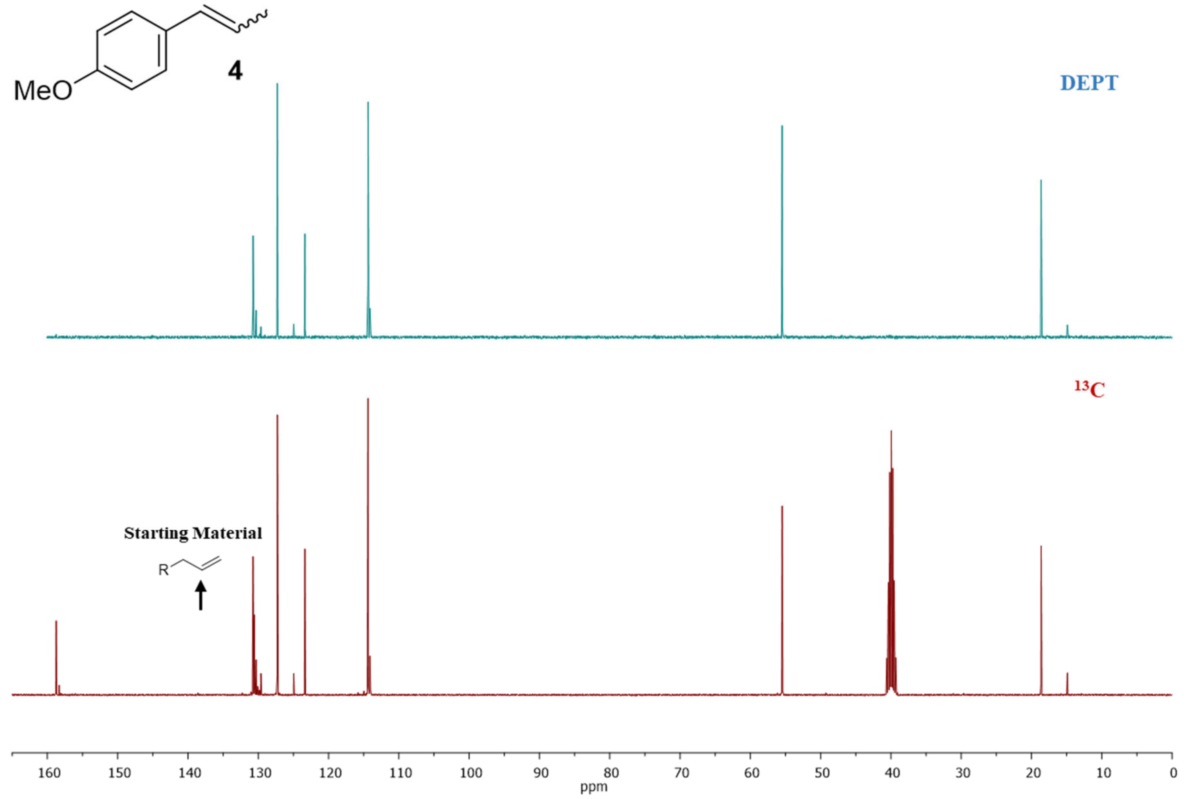

Supplementary Figure 27. ^{13}C-NMR and DEPT of compound **4**, recorded at 100 MHz and 25 ºC in $CDCl_3$.

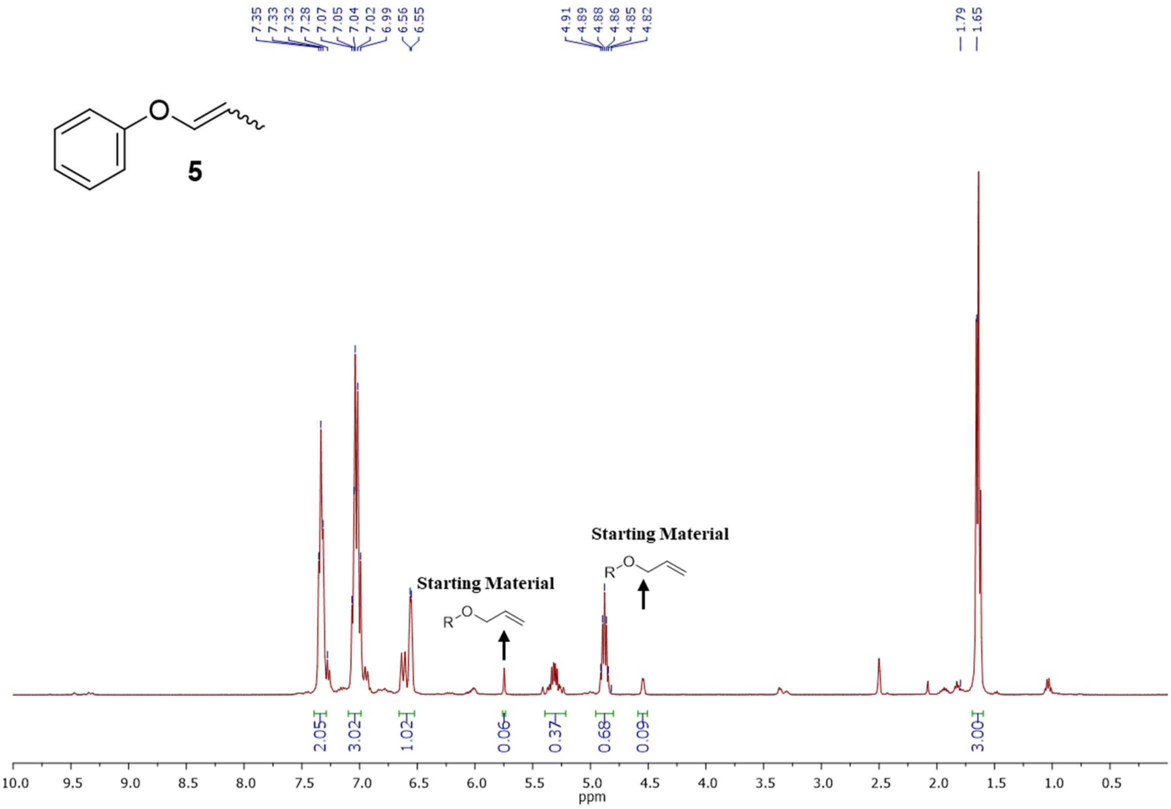

Supplementary Figure 28. ^{1}H-NMR of compound **5**, recorded at 400 MHz and 25 ºC in $CDCl_3$.

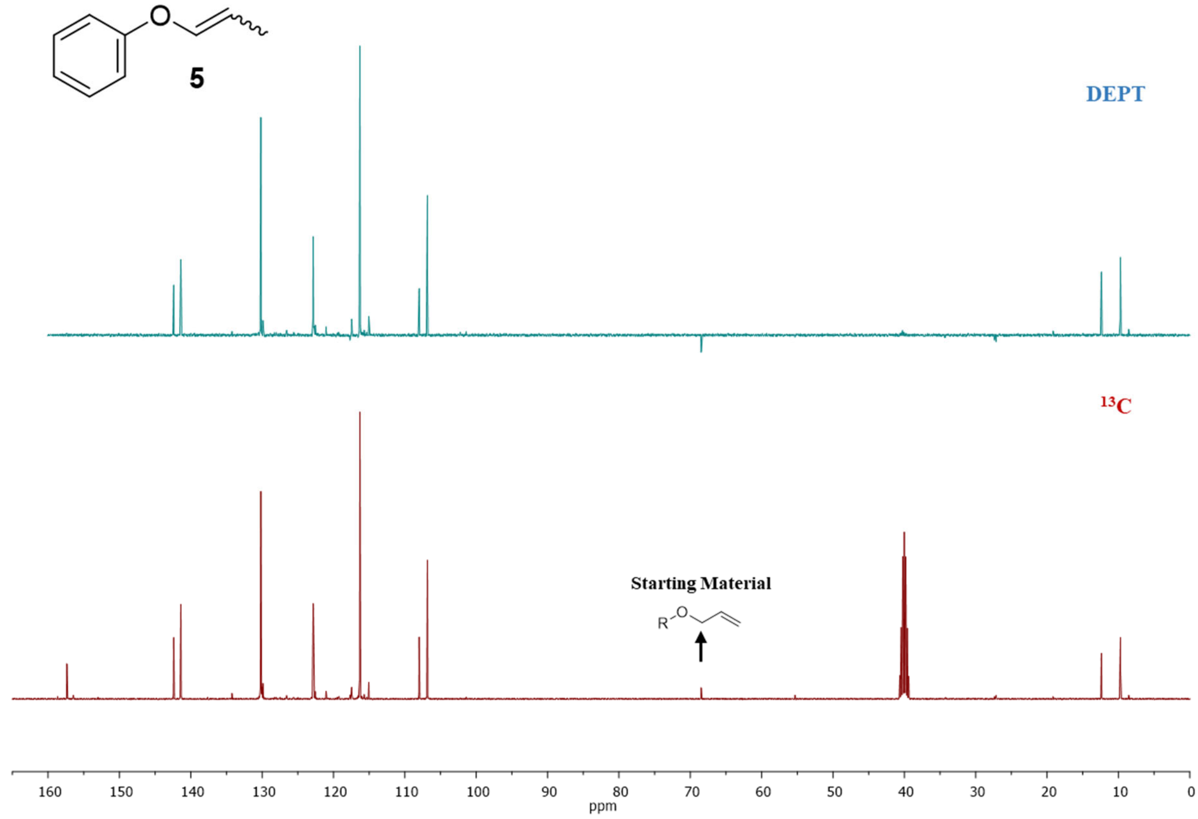

Supplementary Figure 29. ^{13}C-NMR and DEPT of compound **5**, recorded at 100 MHz and 25 ºC in $CDCl_3$.

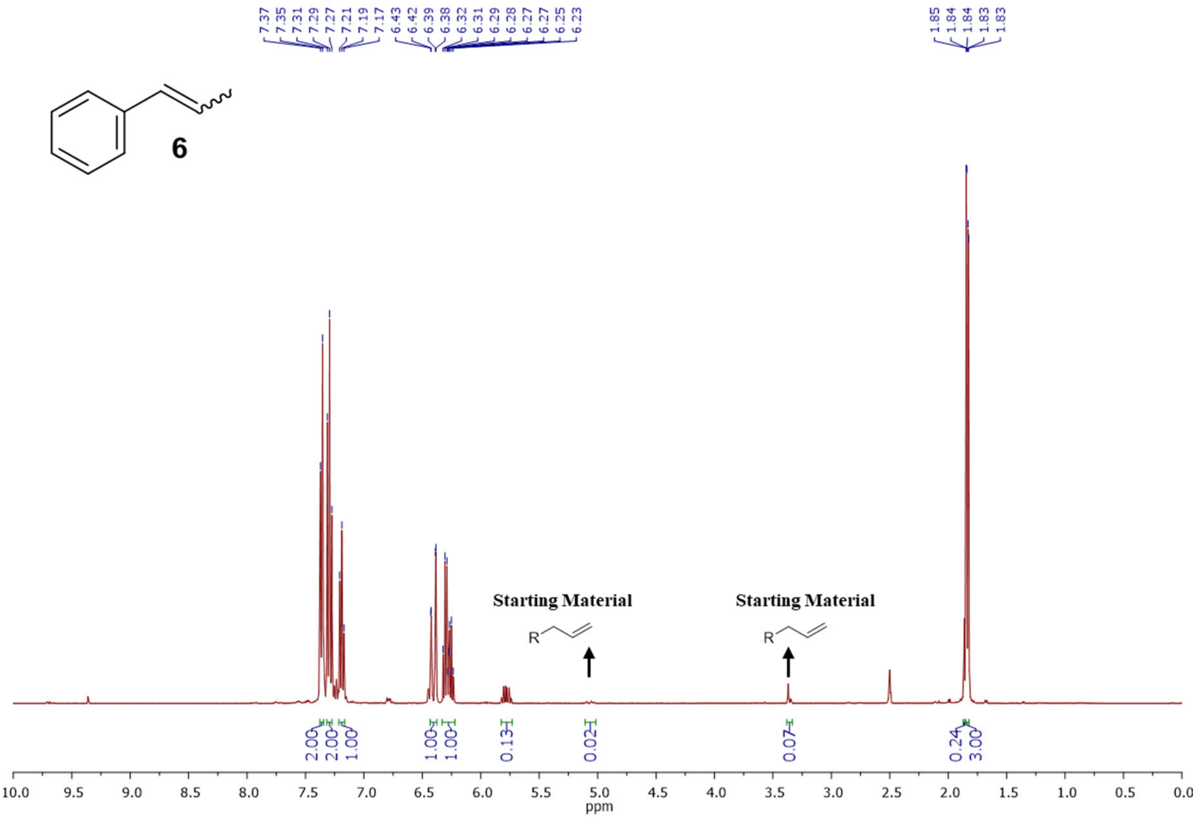

Supplementary Figure 30. ^{1}H-NMR of compound **6**, recorded at 400 MHz and 25 ºC in $CDCl_3$.

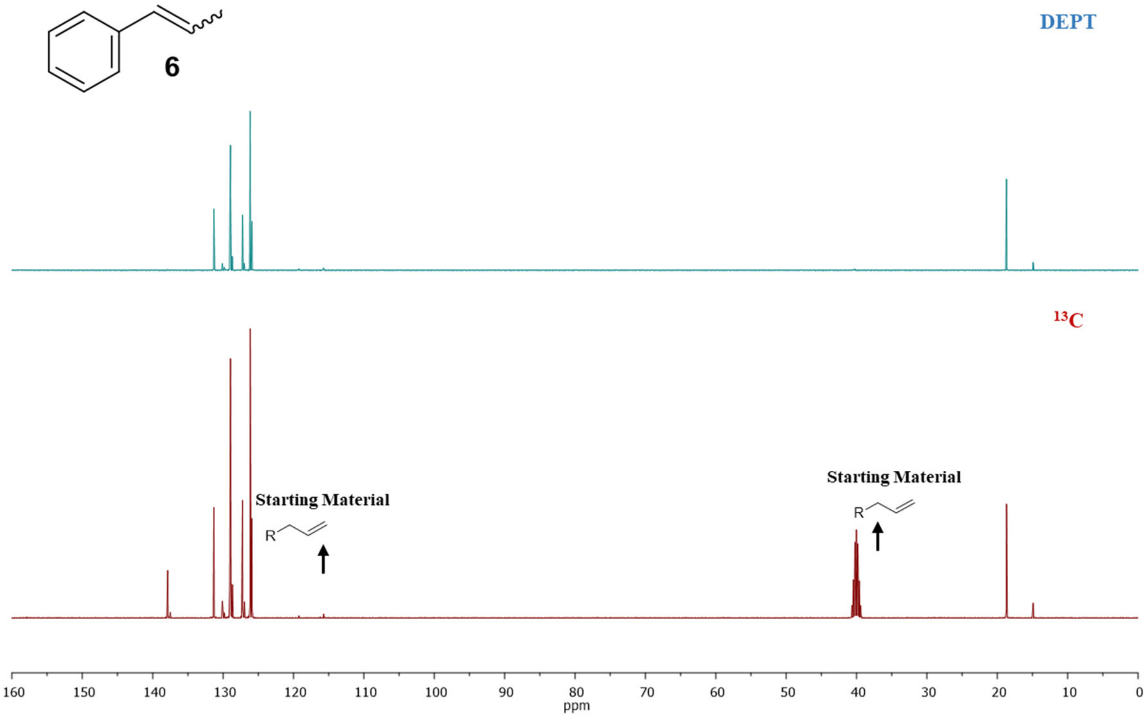

Supplementary Figure 31. ^{13}C-NMR and DEPT of compound **6**, recorded at 100 MHz and 25 ºC in $CDCl_3$.

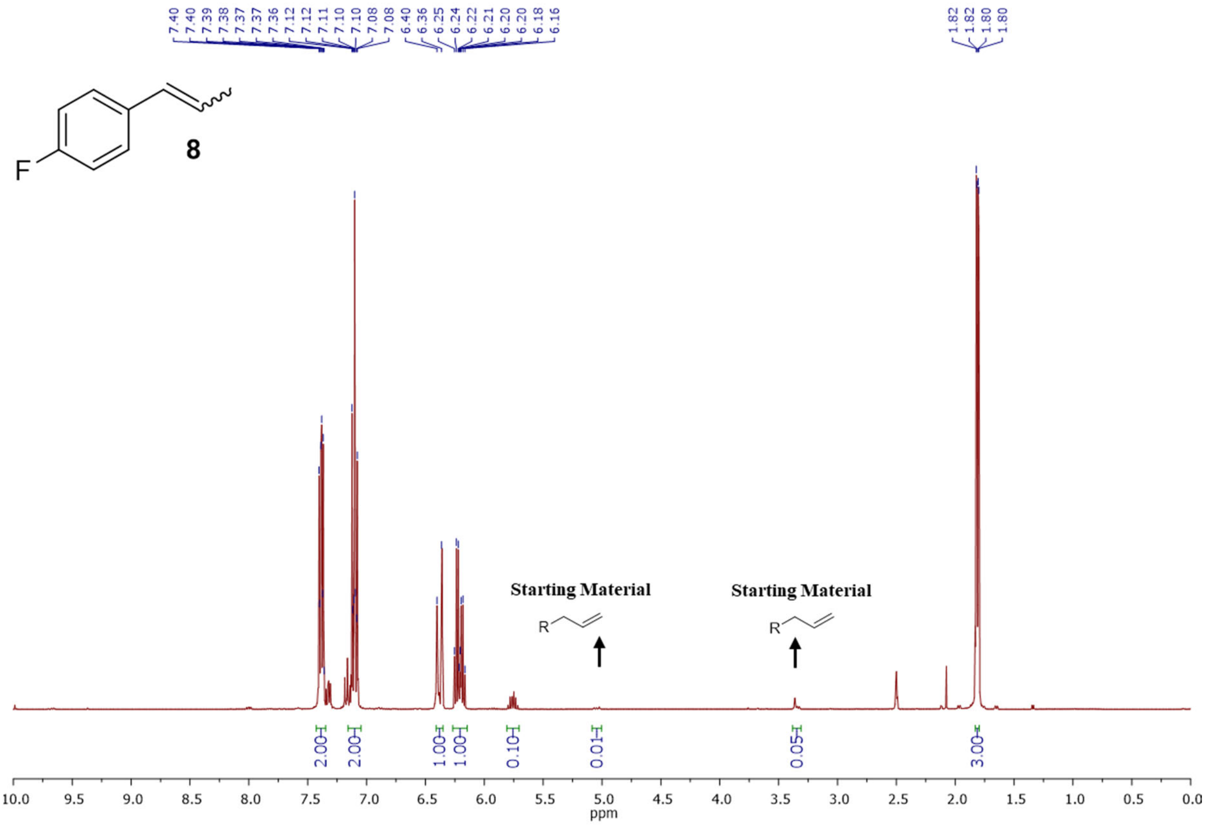

Supplementary Figure 32. ^{1}H-NMR of compound **8**, recorded at 400 MHz and 25 ºC in $CDCl_3$.

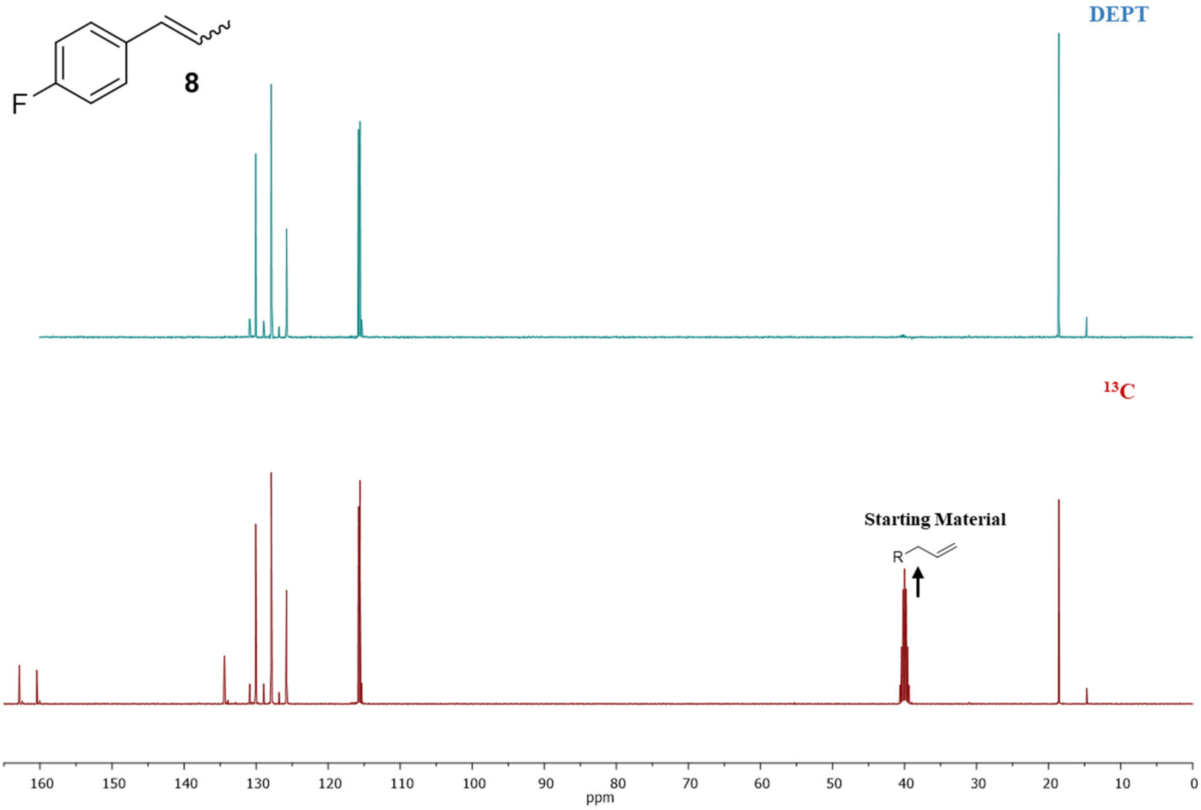

Supplementary Figure 33. ^{13}C-NMR and DEPT of compound **8**, recorded at 100 MHz and 25 ºC in $CDCl_3$.

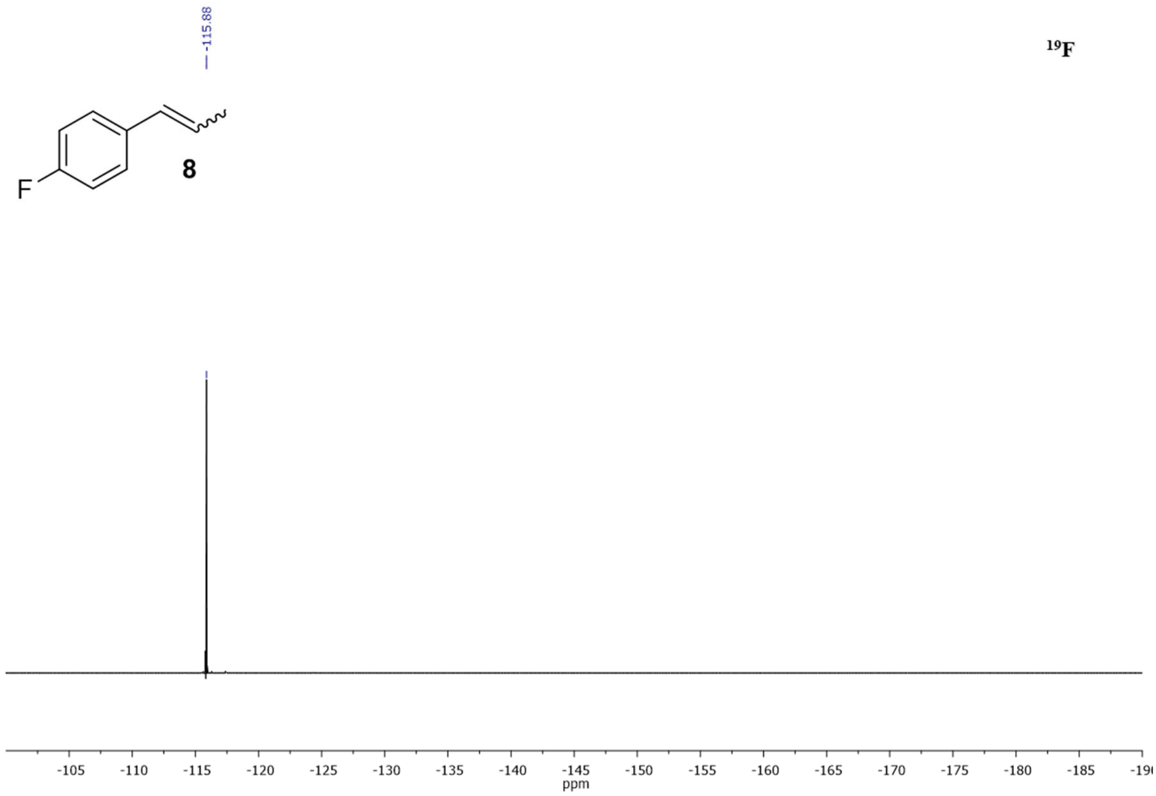

Supplementary Figure 34. ^{19}F-NMR of compound **8**, recorded at 400 MHz and 25 ºC in $CDCl_3$.

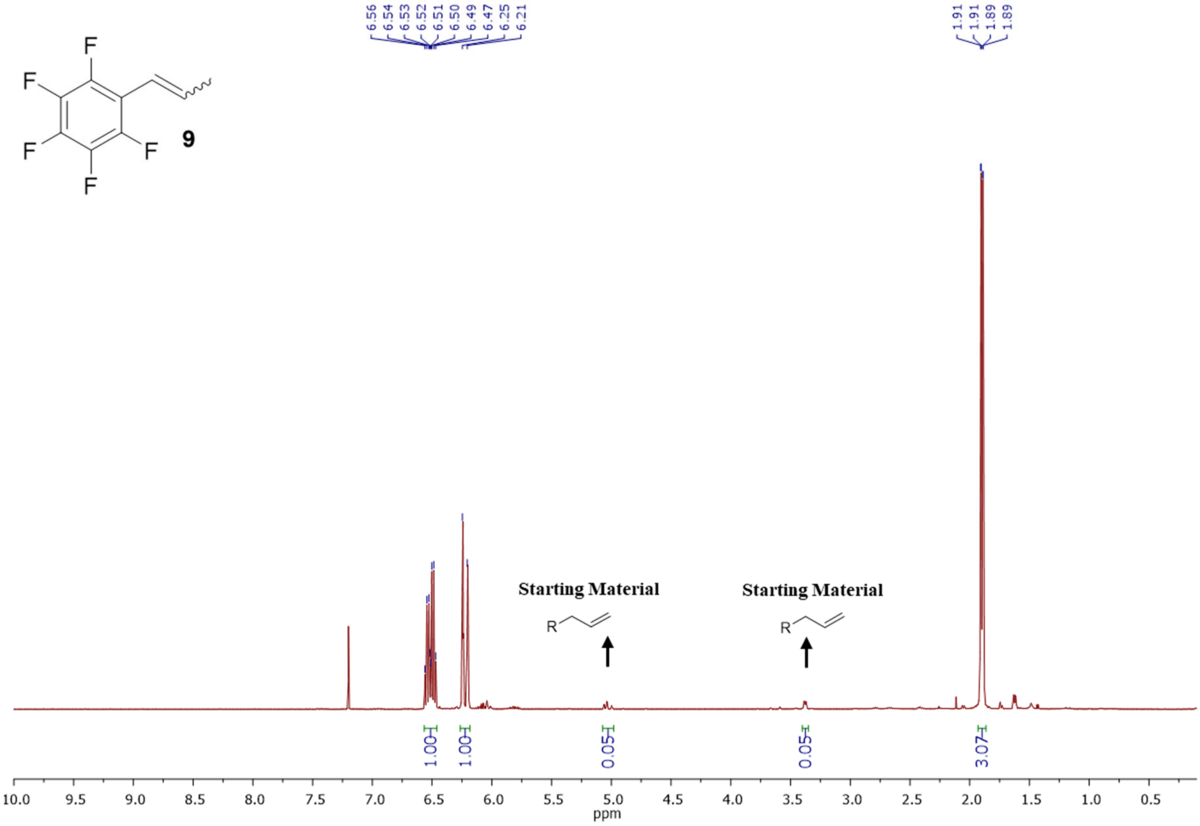

Supplementary Figure 35. ^{1}H-NMR of compound **9**, recorded at 400 MHz and 25 ºC in $CDCl_3$.

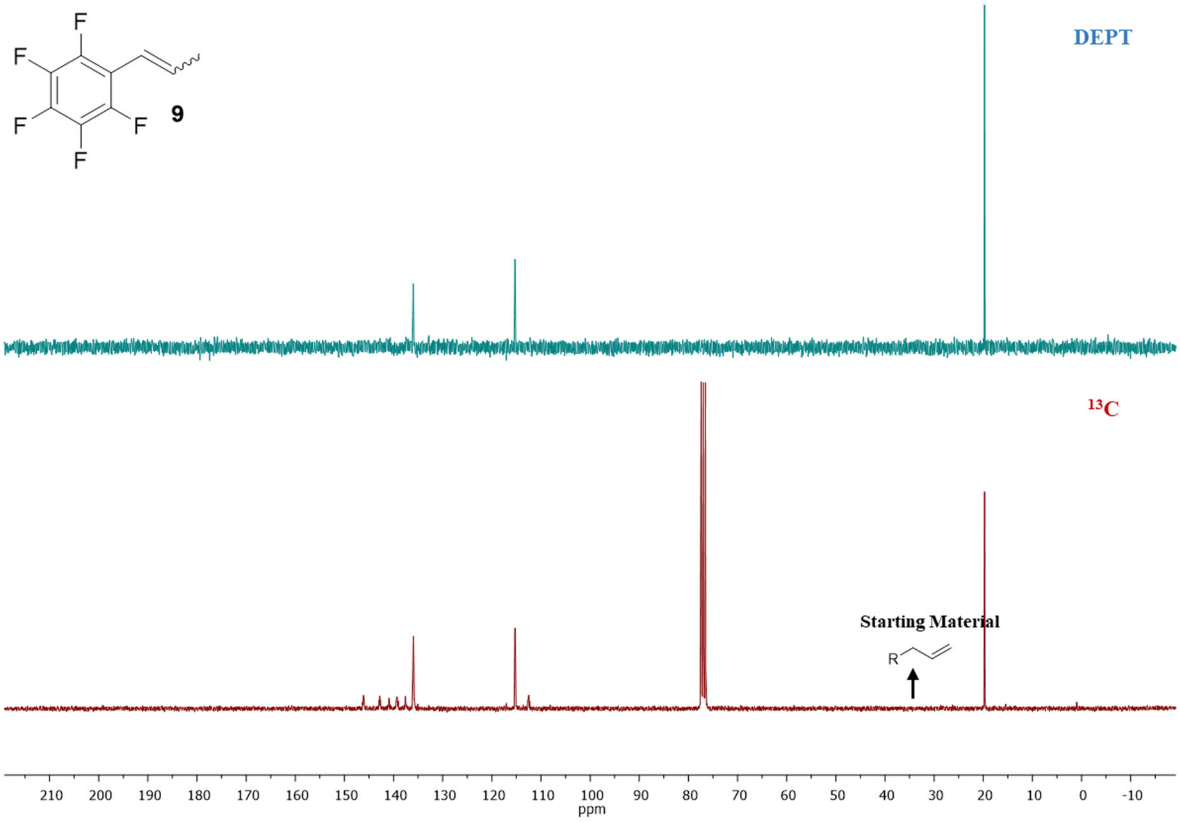

Supplementary Figure 36. ^{13}C-NMR and DEPT of compound **9**, recorded at 100 MHz and 25 ºC in $CDCl_3$.

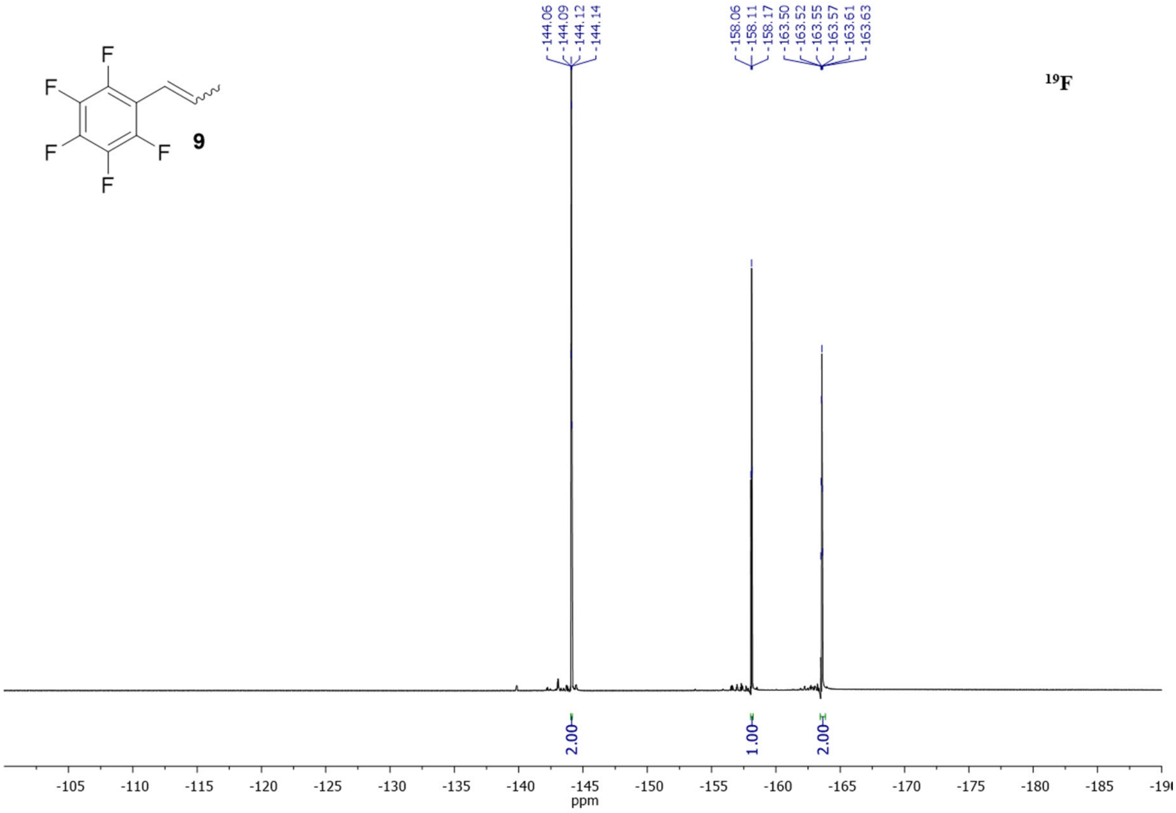

Supplementary Figure 37. ^{19}F-NMR of compound **9**, recorded at 400 MHz and 25 ºC in $CDCl_3$.

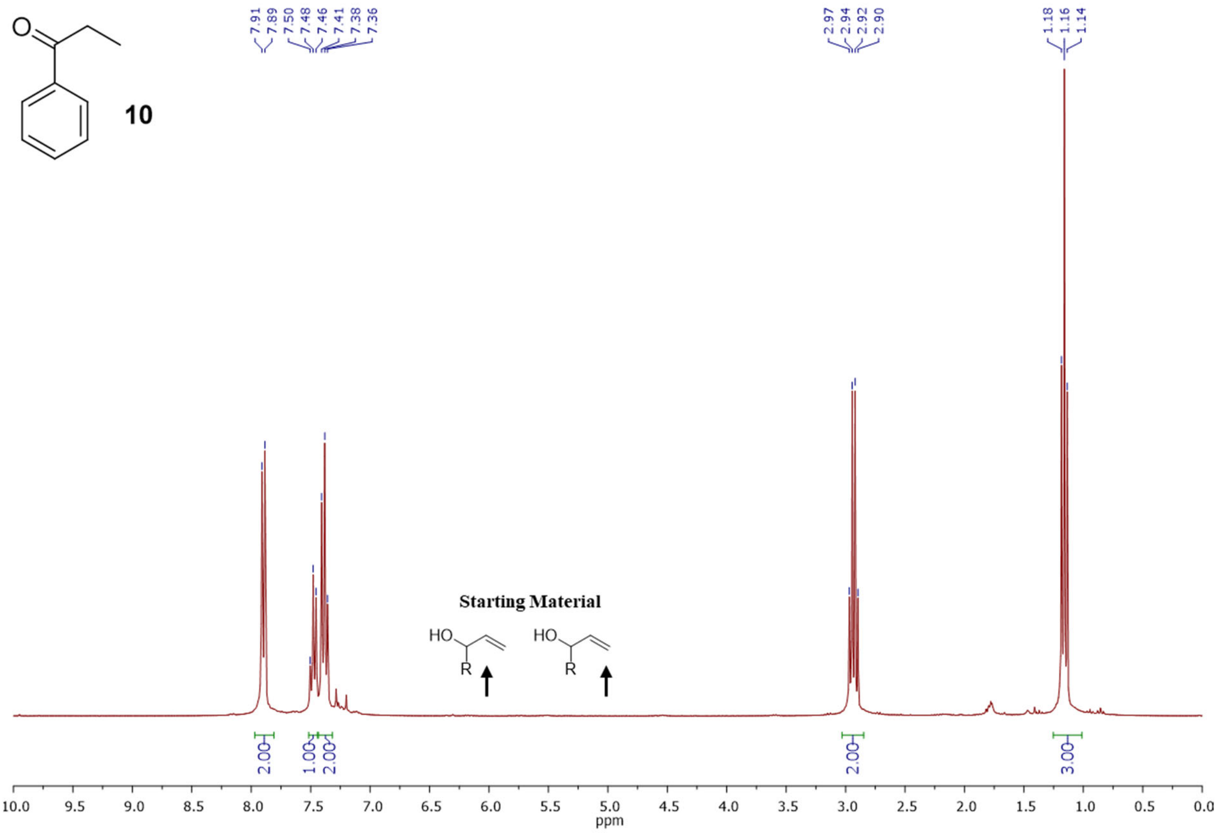

Supplementary Figure 38. ^{1}H-NMR of compound **10**, recorded at 400 MHz and 25 ºC in $CDCl_3$.

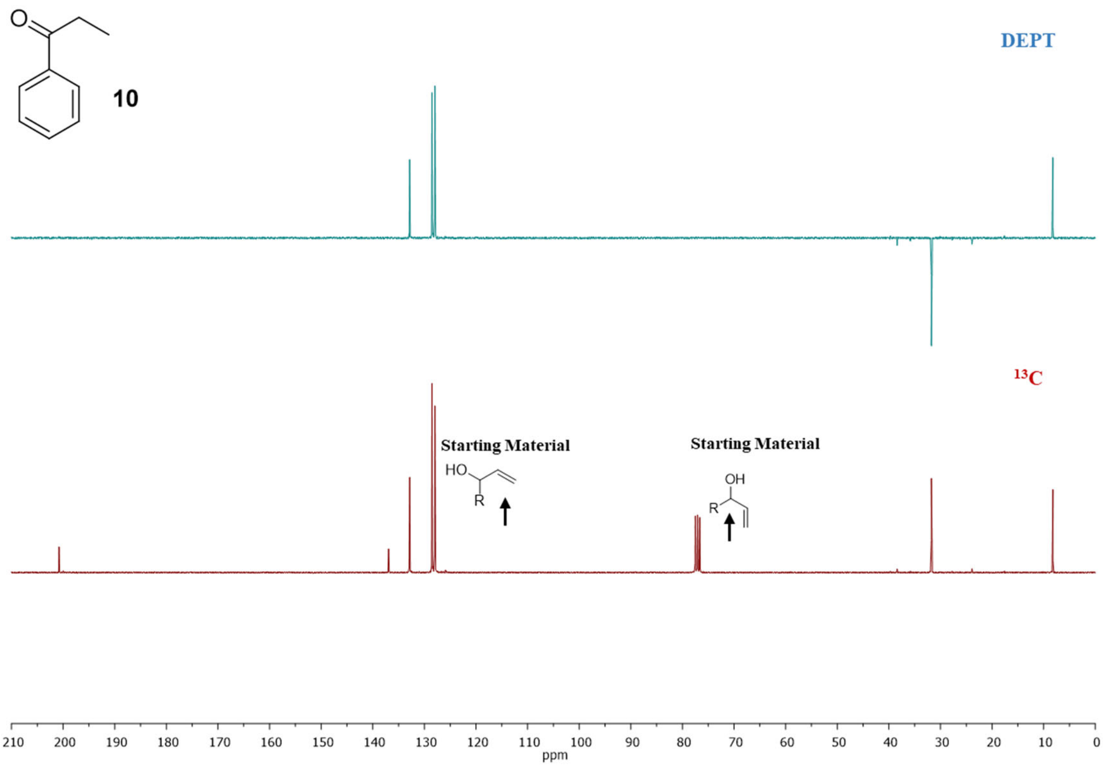

Supplementary Figure 39. ^{13}C-NMR and DEPT of compound **10**, recorded at 100 MHz and 25 ºC in $CDCl_3$.

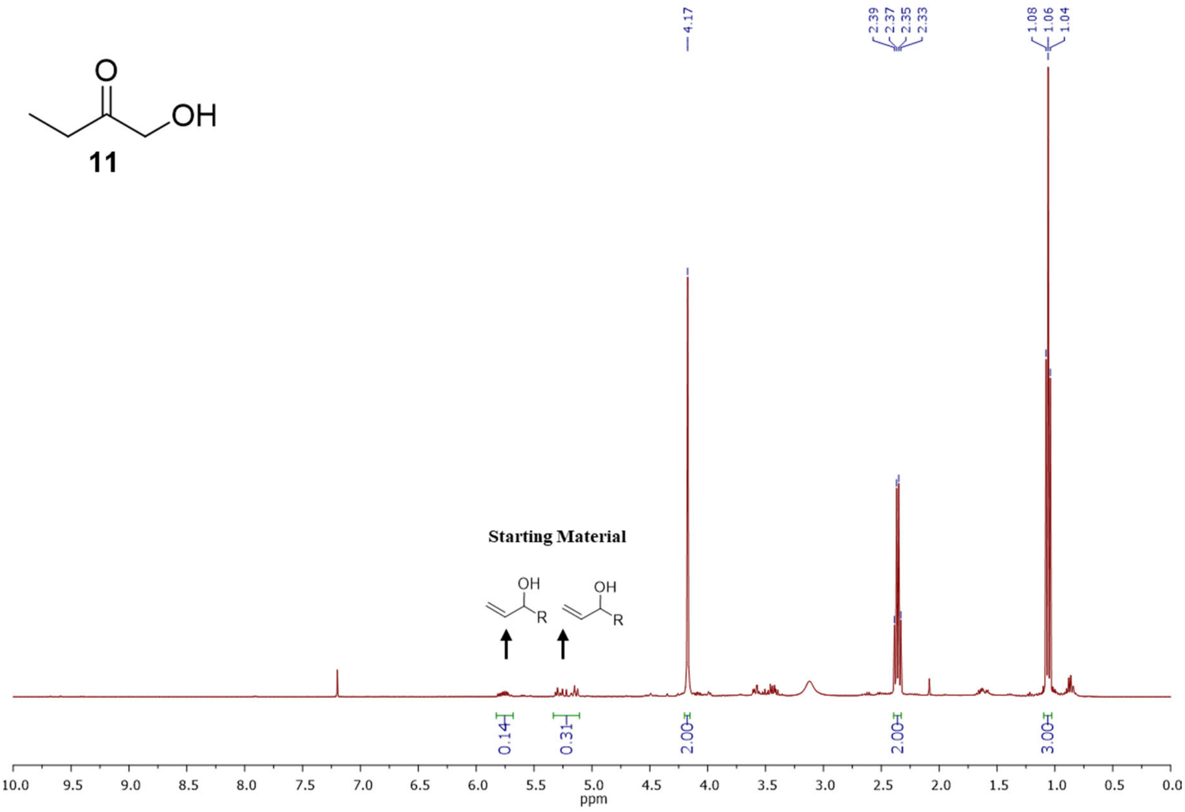

Supplementary Figure 40. ^{1}H-NMR of compound **11**, recorded at 400 MHz and 25 ºC in $CDCl_3$.

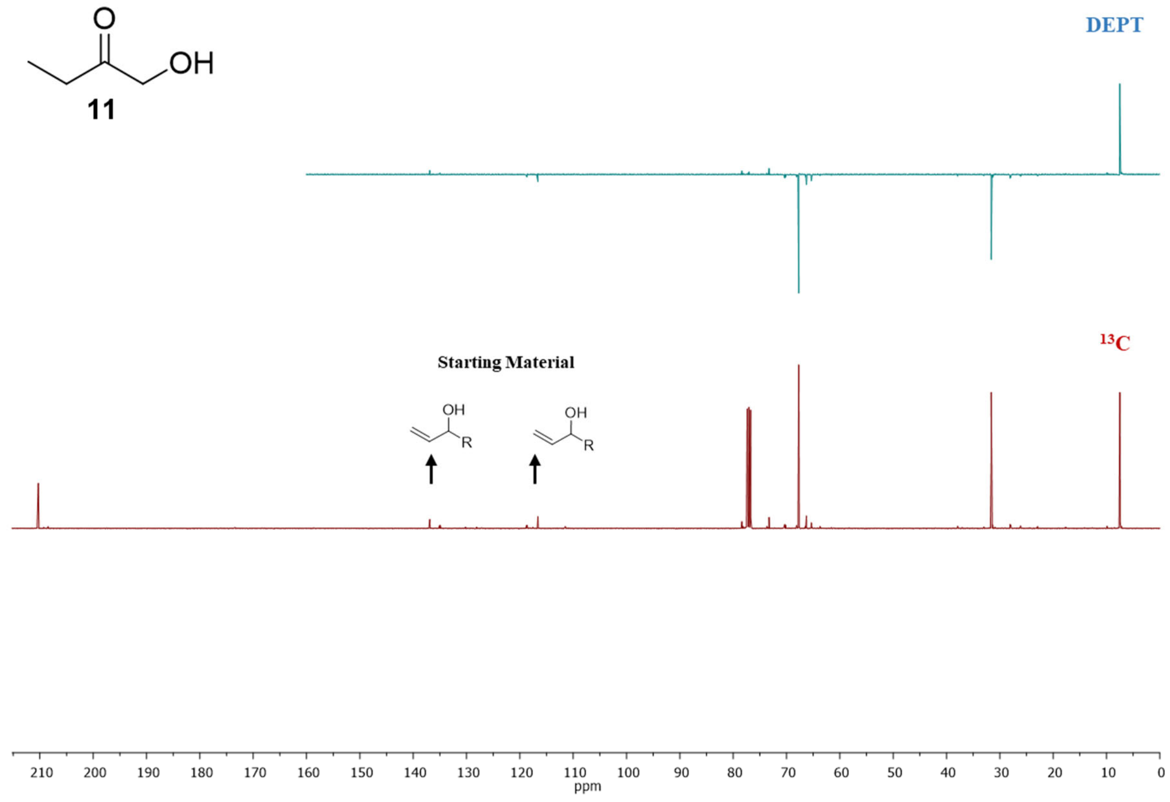

Supplementary Figure 41. ^{13}C-NMR and DEPT of compound **11**, recorded at 100 MHz and 25 ºC in $CDCl_3$.

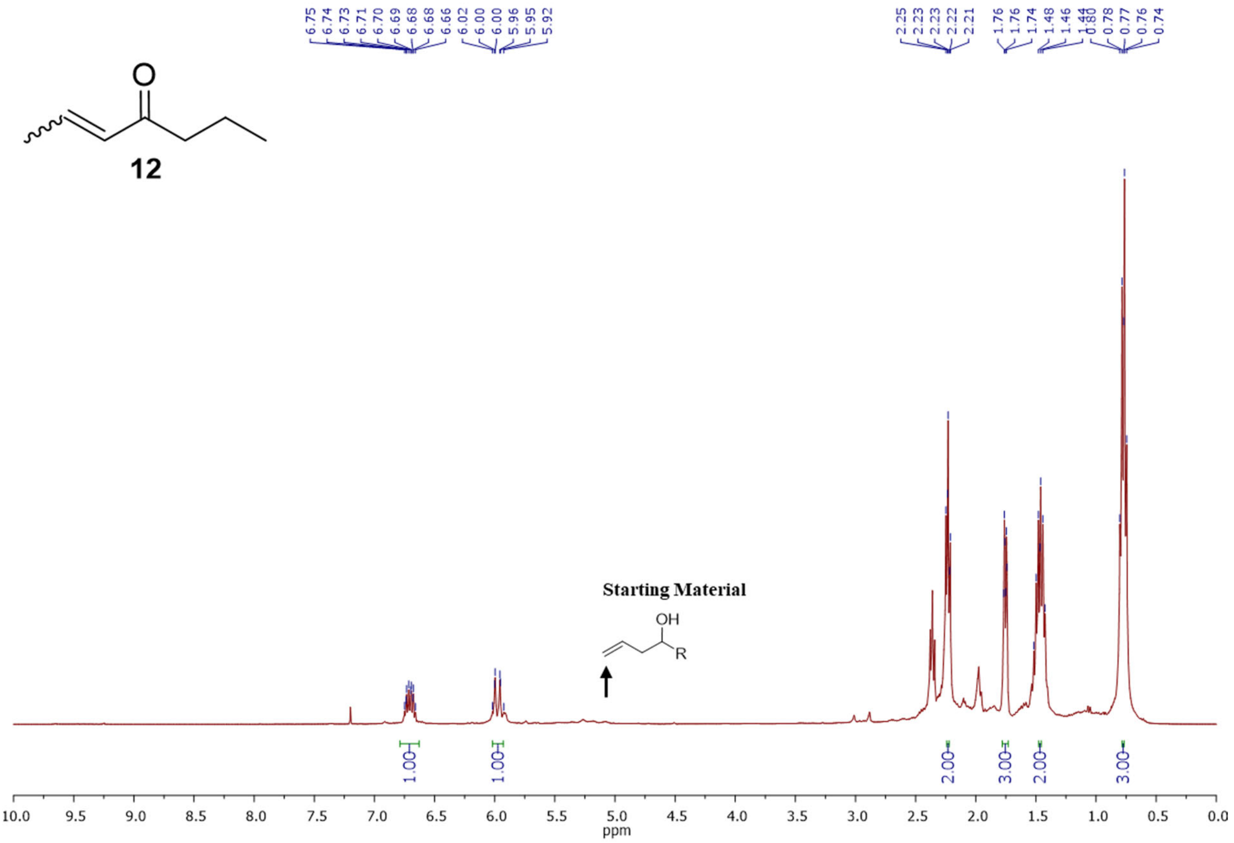

Supplementary Figure 42. ^{1}H-NMR of compound **12**, recorded at 400 MHz and 25 ºC in $CDCl_3$.

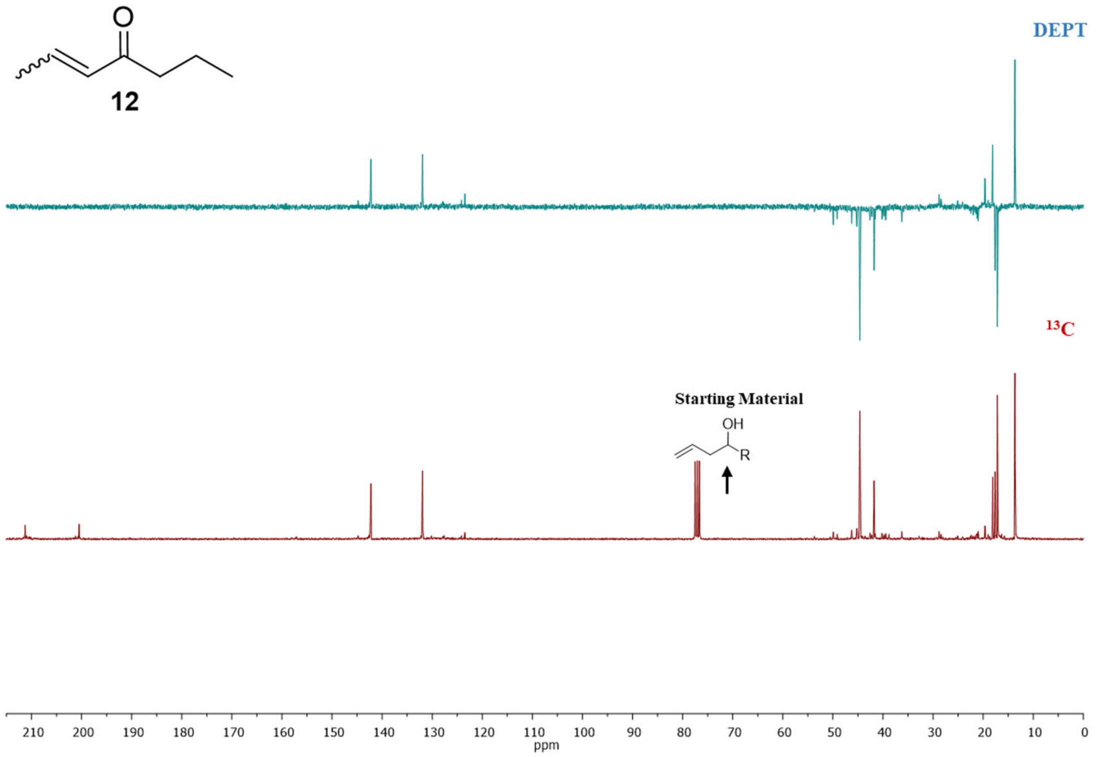

Supplementary Figure 43. ^{13}C-NMR and DEPT of compound **12**, recorded at 100 MHz and 25 ºC in $CDCl_3$.

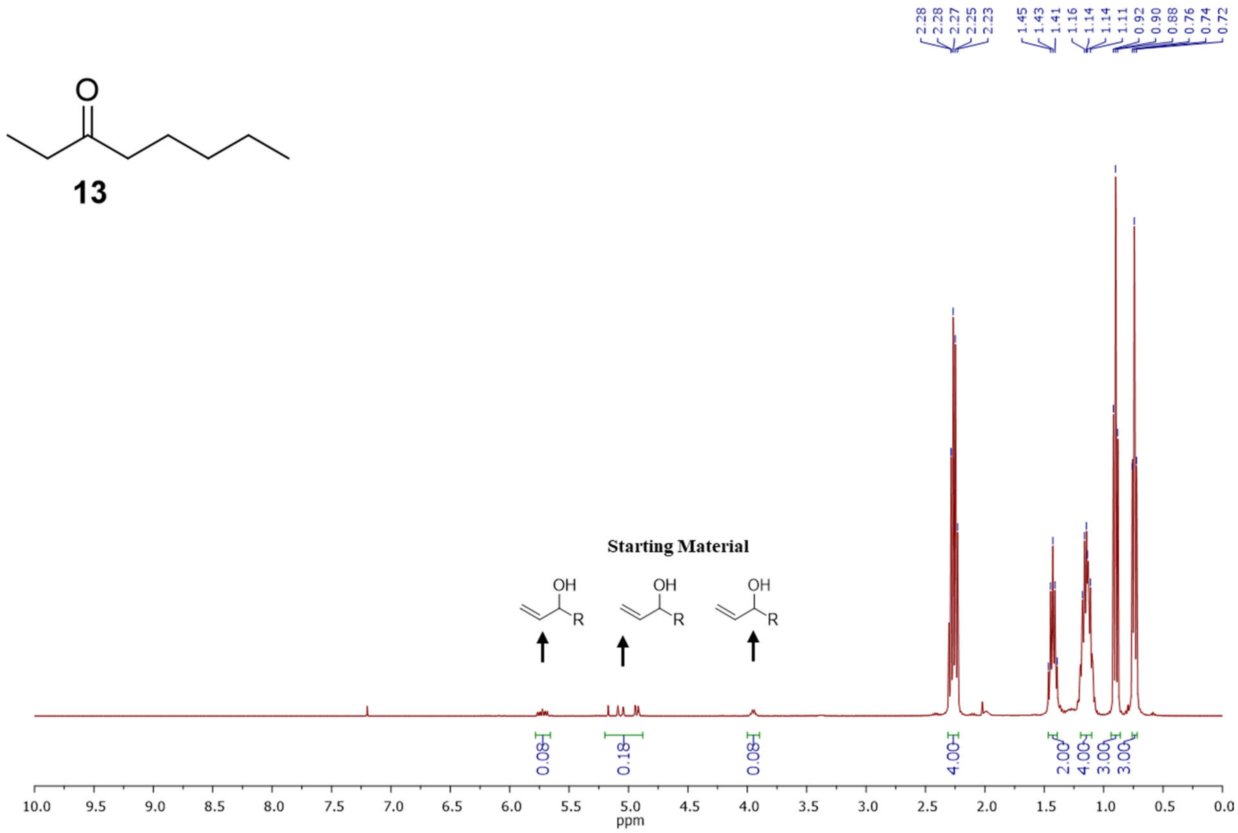

Supplementary Figure 44. ^{1}H-NMR of compound **13**, recorded at 400 MHz and 25 ºC in $CDCl_3$.

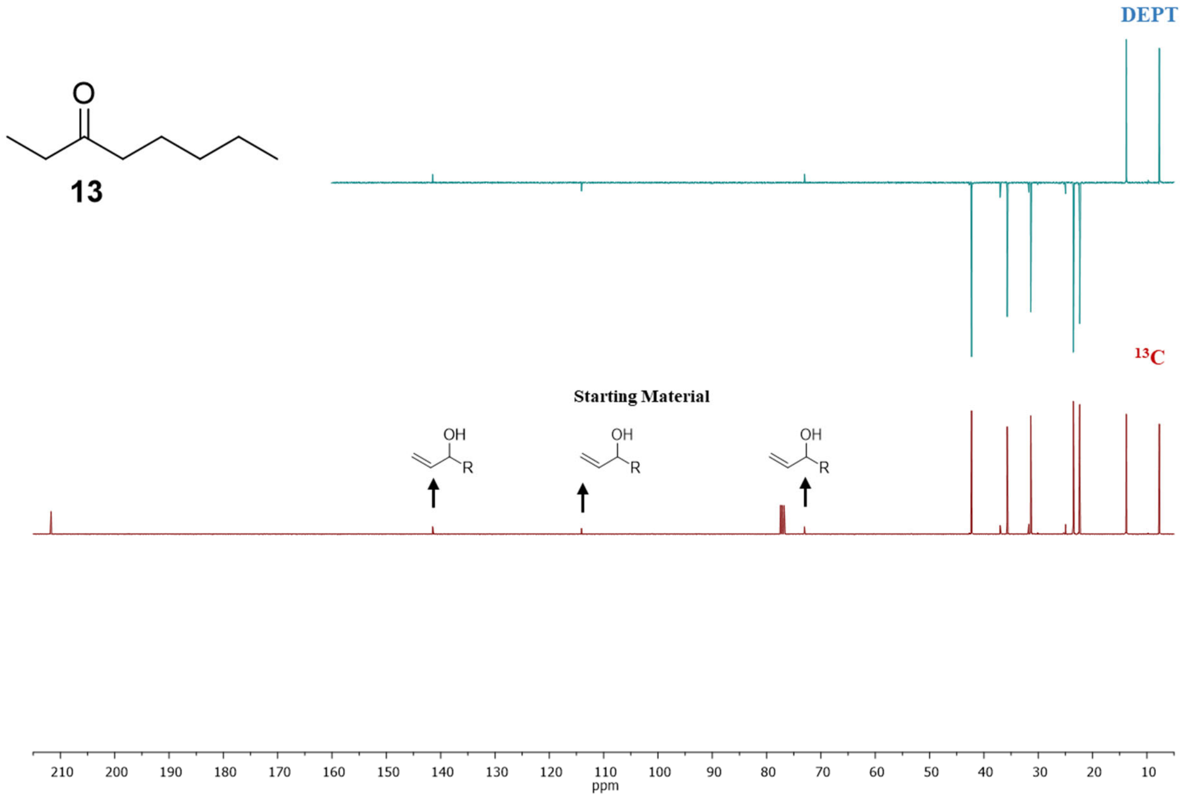

Supplementary Figure 45. ^{13}C-NMR and DEPT of compound **13**, recorded at 100 MHz and 25 ºC in $CDCl_3$.

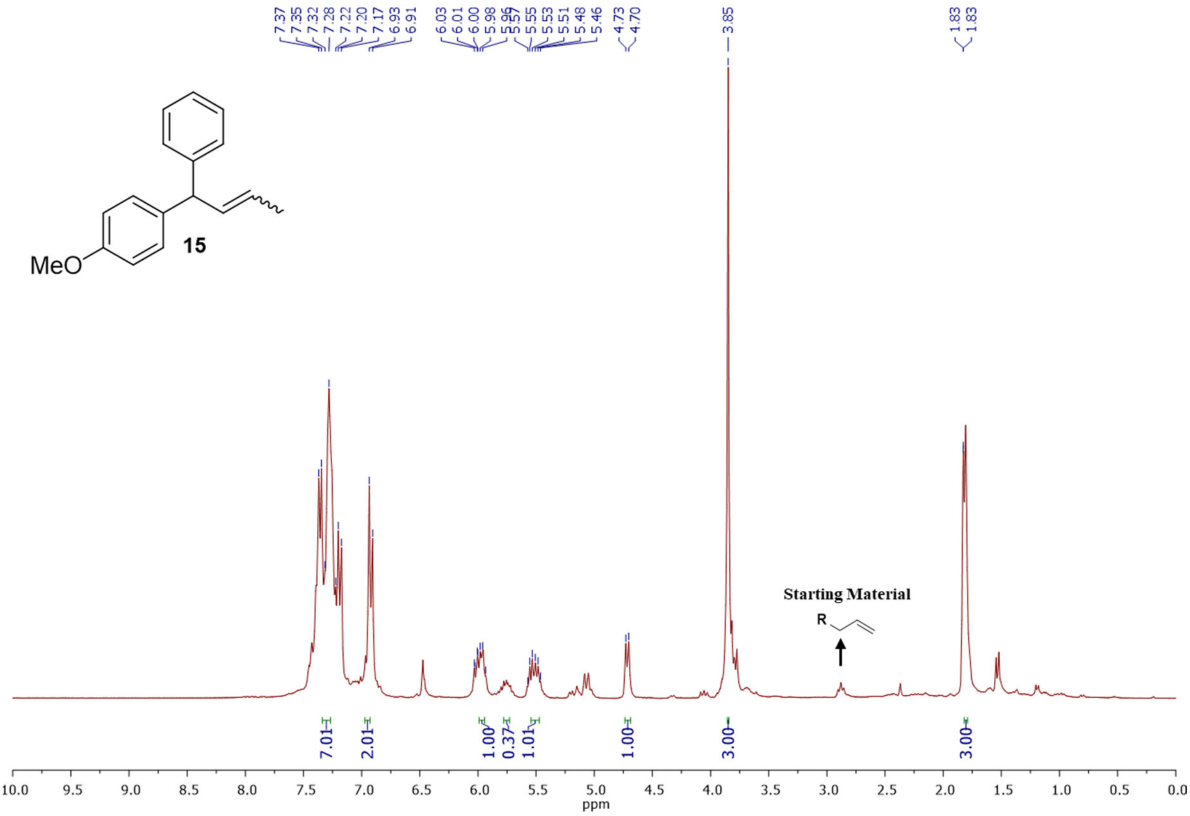

Supplementary Figure 46. ^{1}H-NMR of compound **15**, recorded at 400 MHz and 25 ºC in $CDCl_3$.

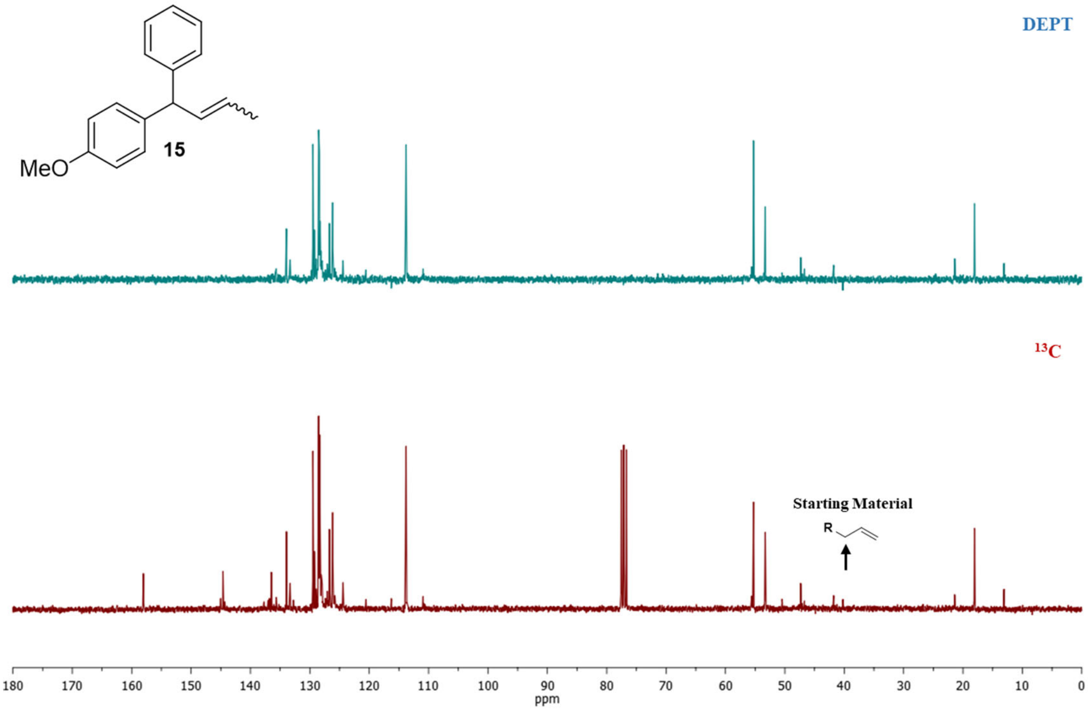

Supplementary Figure 47. ^{13}C-NMR and DEPT of compound **15**, recorded at 100 MHz and 25 ºC in $CDCl_3$.

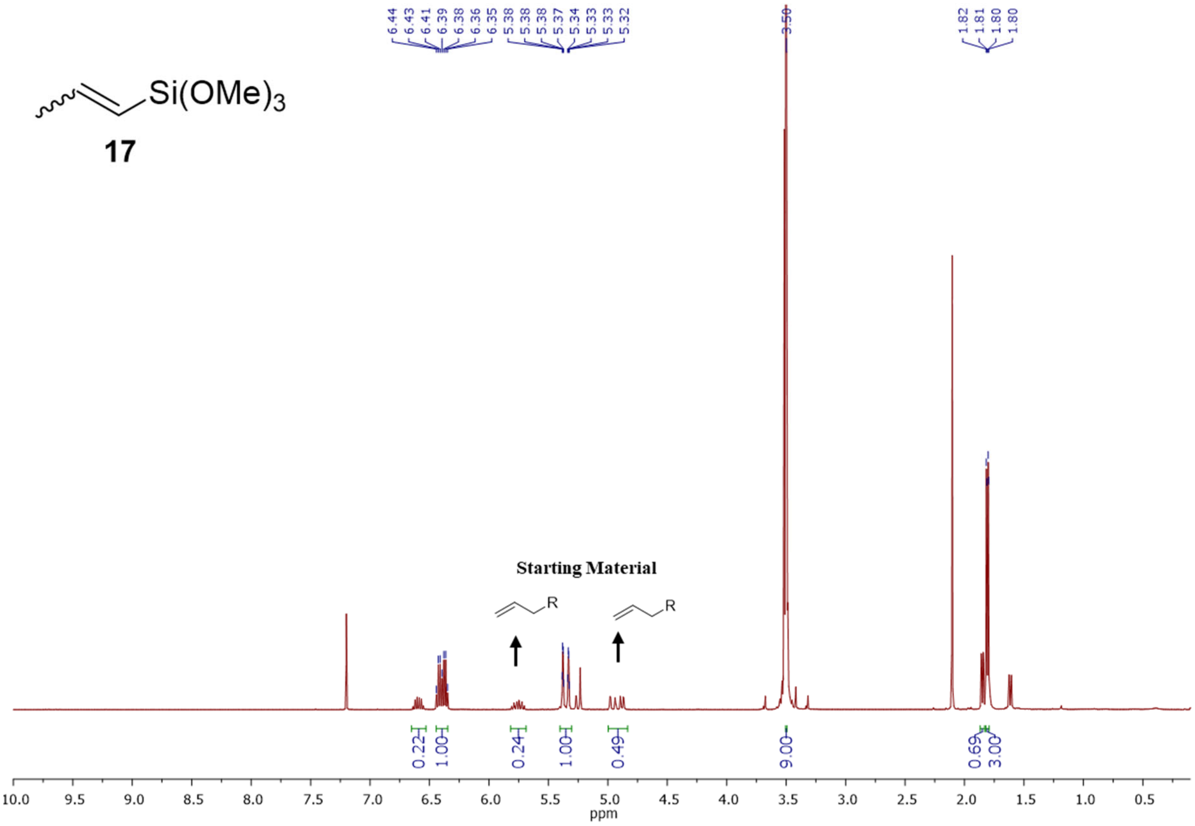

Supplementary Figure 48. ^{1}H-NMR of compound **17**, recorded at 400 MHz and 25 ºC in $CDCl_3$.

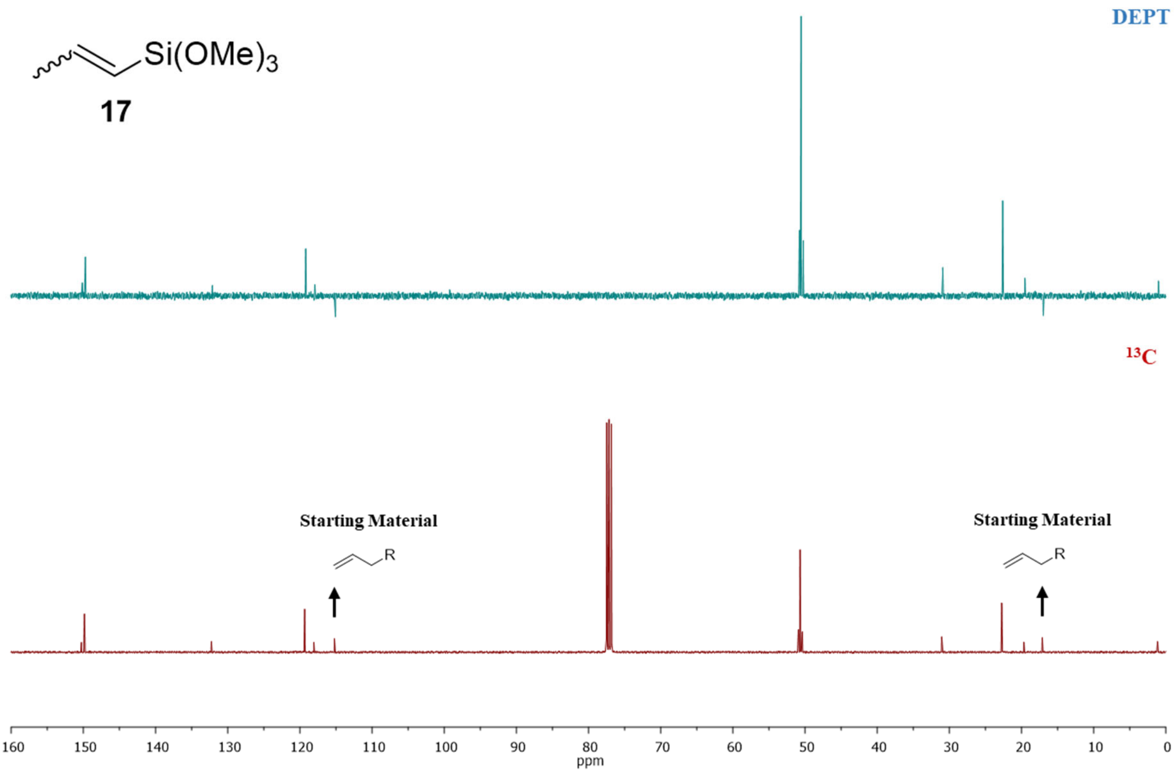

Supplementary Figure 49. ^{13}C-NMR and DEPT of compound **17**, recorded at 100 MHz and 25 ºC in $CDCl_3$.

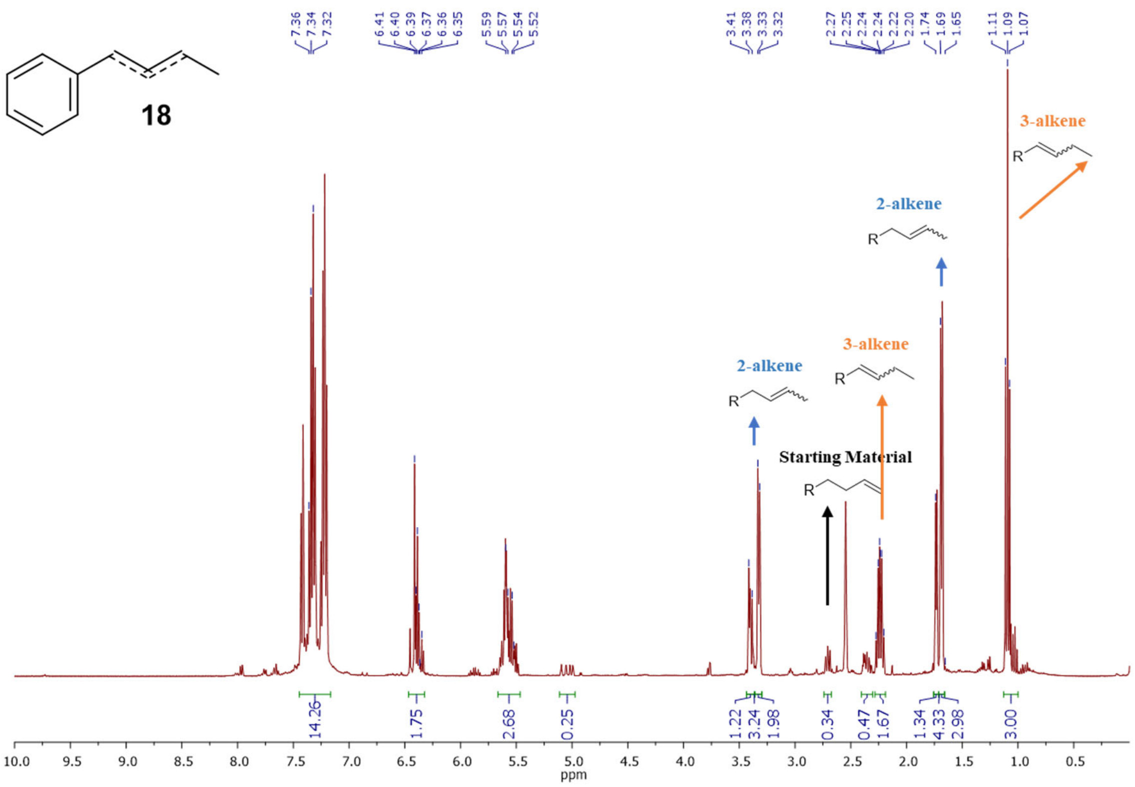

Supplementary Figure 50. ^{1}H-NMR of compound **18**, recorded at 400 MHz and 25 ºC in $CDCl_3$.

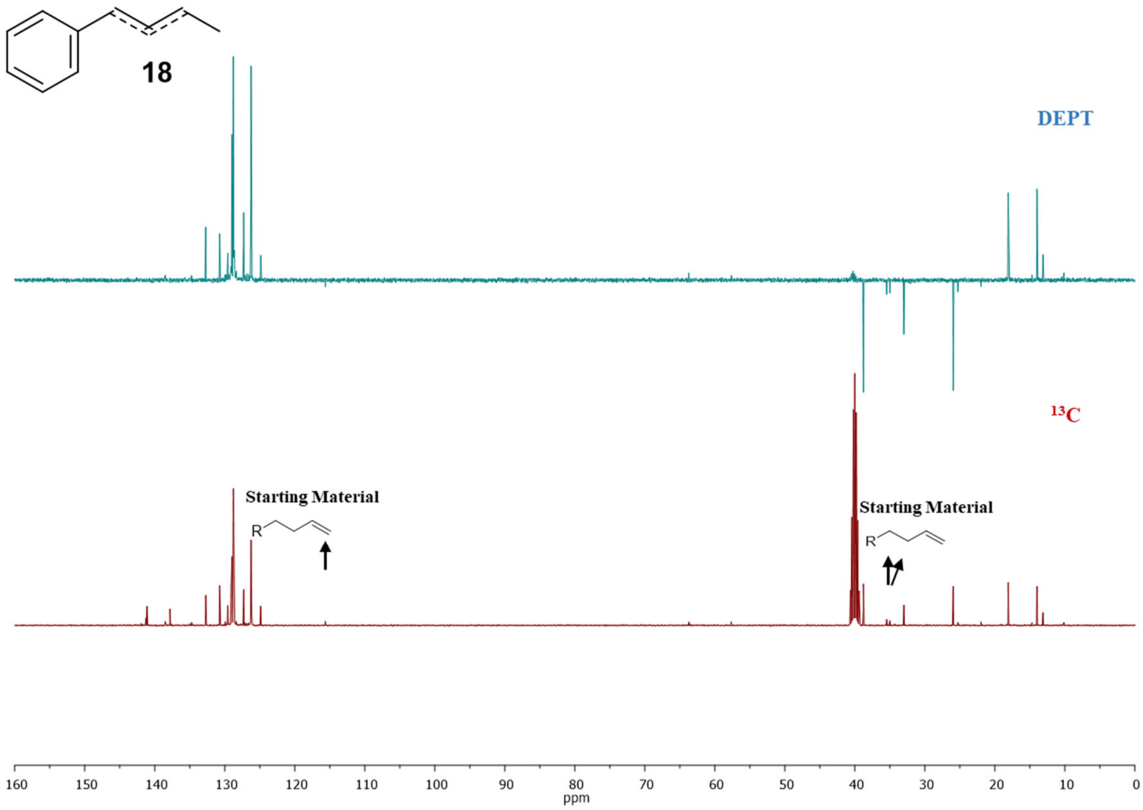

Supplementary Figure 51. ^{13}C-NMR and DEPT of compound **18**, recorded at 100 MHz and 25 ºC in $CDCl_3$.

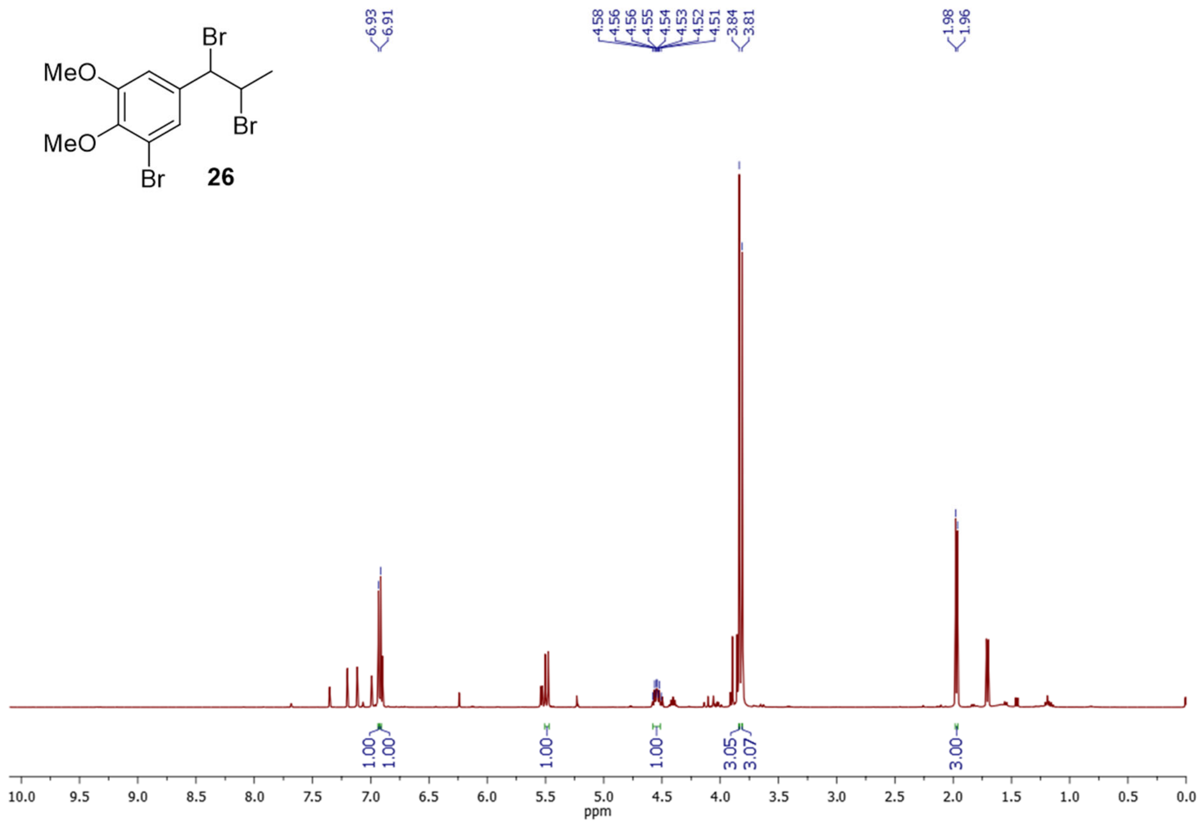

Supplementary Figure 52. ^{1}H-NMR of compound **26**, recorded at 400 MHz and 25 ºC in $CDCl_3$.

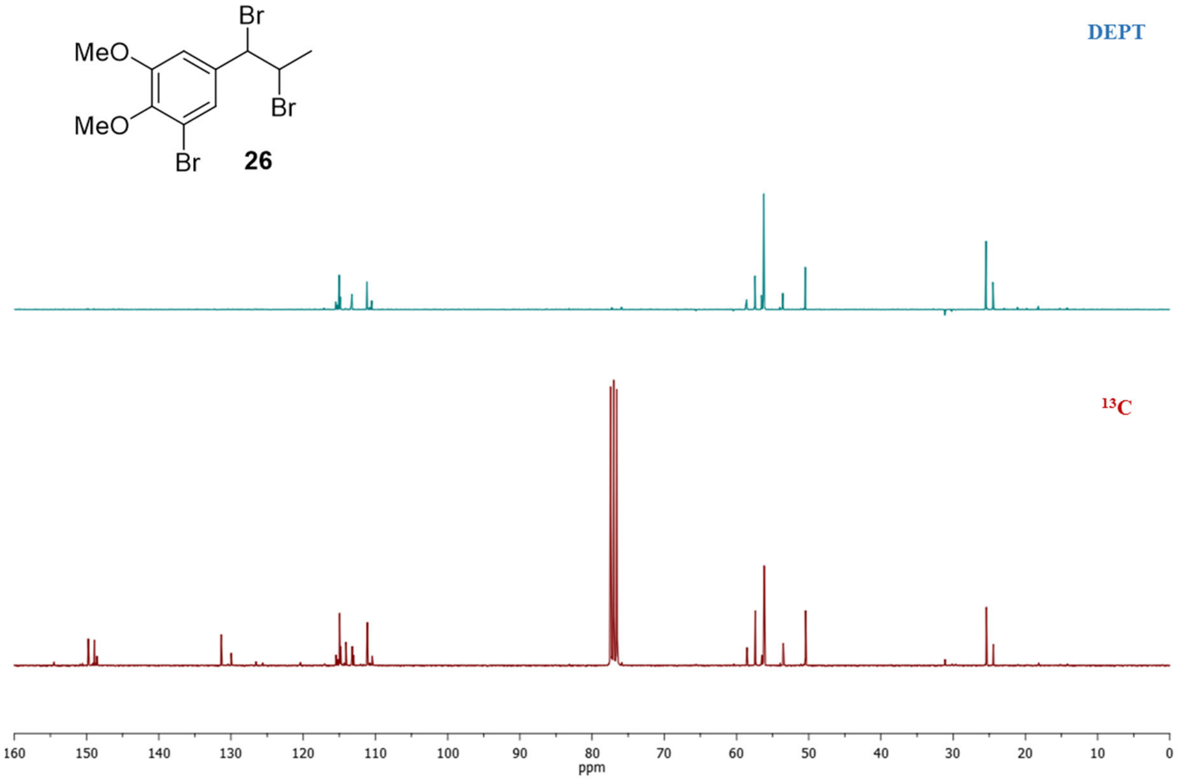

Supplementary Figure 53. ^{13}C-NMR and DEPT of compound **26**, recorded at 100 MHz and 25 ºC in $CDCl_3$.

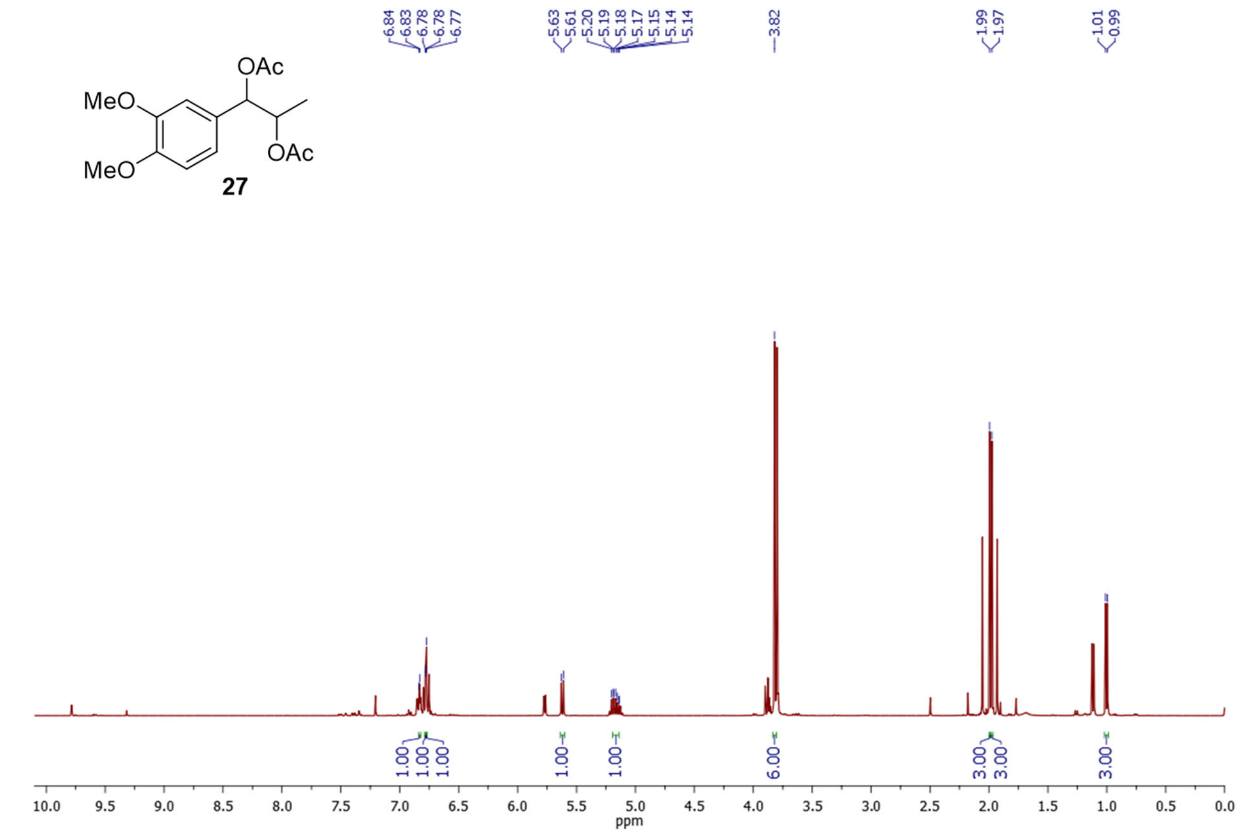

Supplementary Figure 54. ^{1}H-NMR of compound **27**, recorded at 400 MHz and 25 ºC in $CDCl_3$.

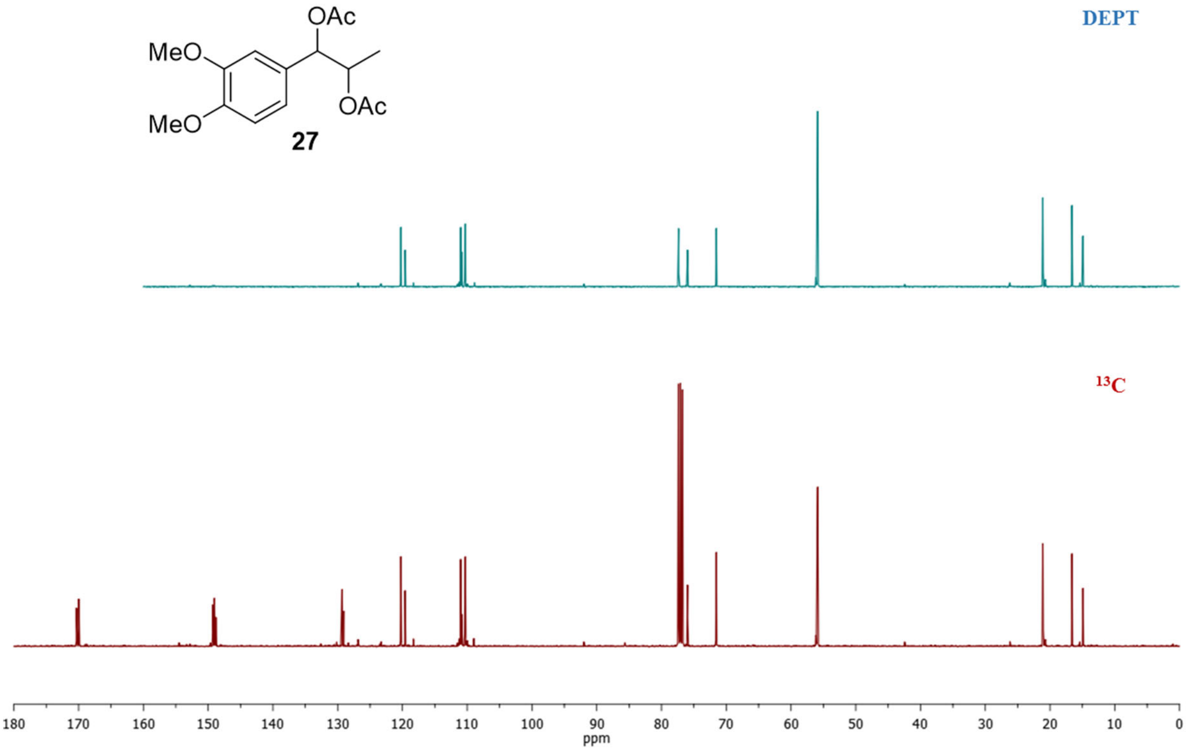

Supplementary Figure 55. ^{13}C-NMR and DEPT of compound **27**, recorded at 100 MHz and 25 ºC in $CDCl_3$.

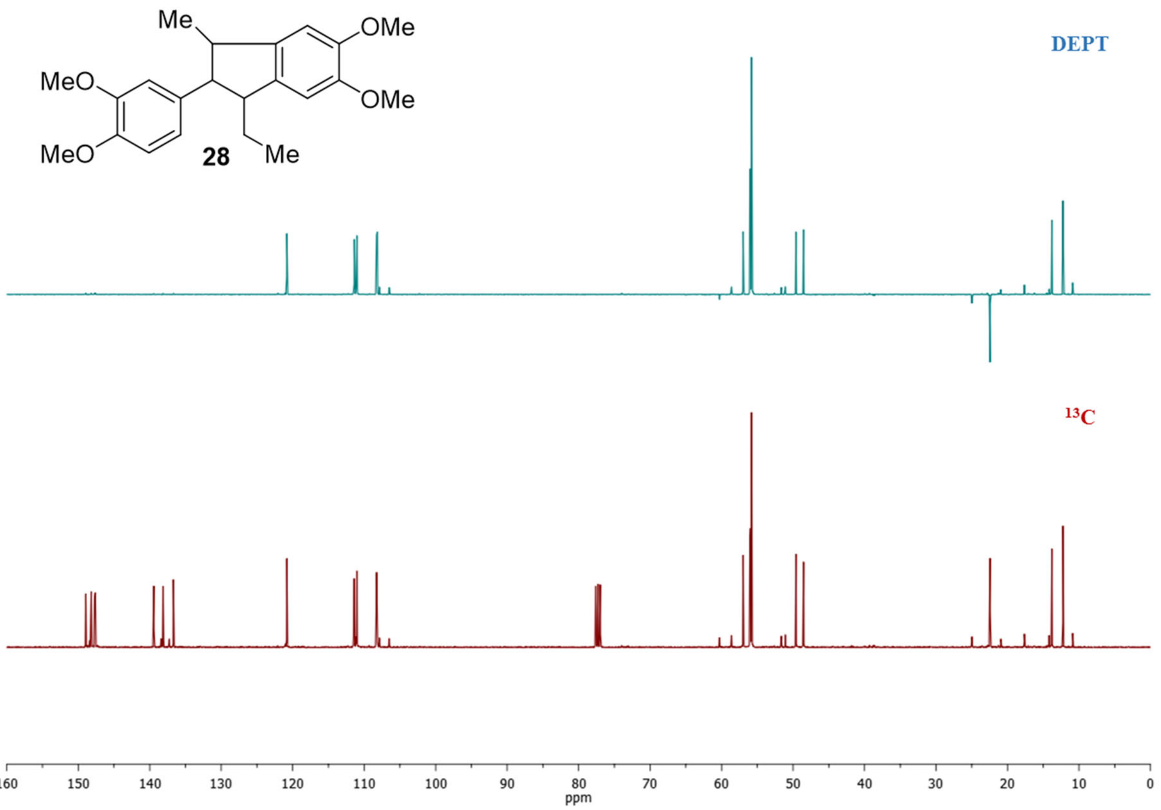

Supplementary Figure 56. ^{1}H-NMR of compound **28**, recorded at 400 MHz and 25 ºC in $CDCl_3$.

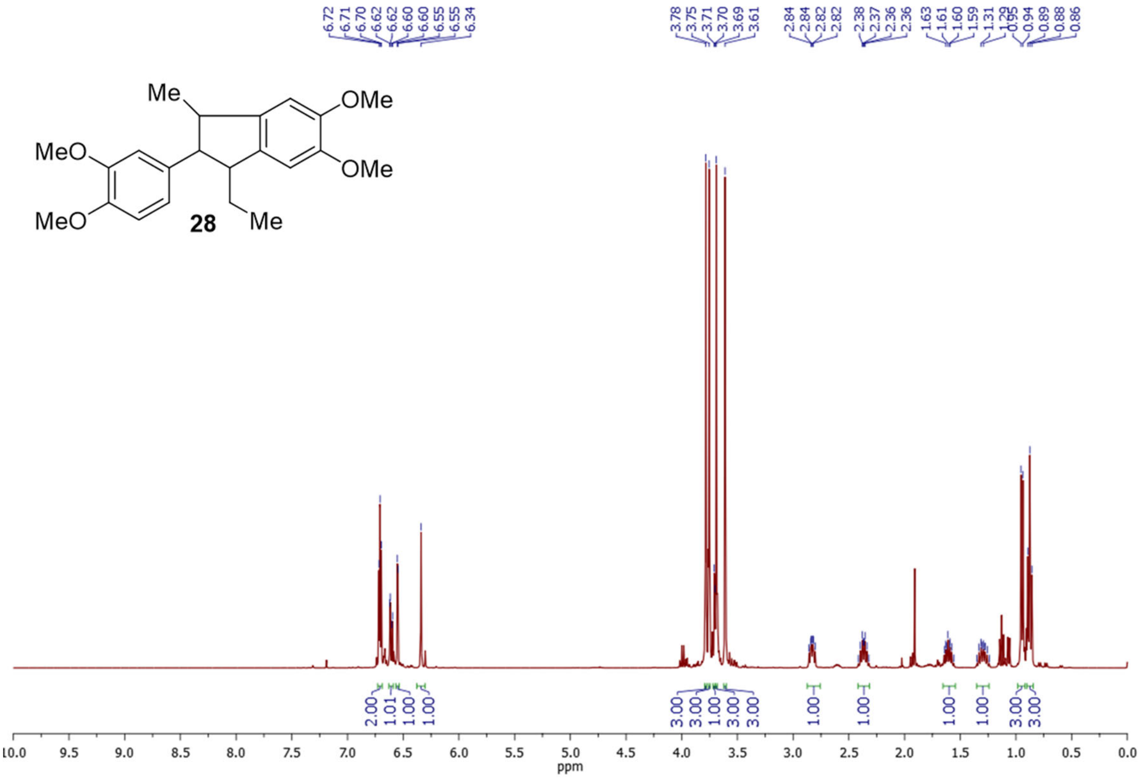

Supplementary Figure 57. ^{13}C-NMR and DEPT of compound **28**, recorded at 100 MHz and 25 ºC in $CDCl_3$.

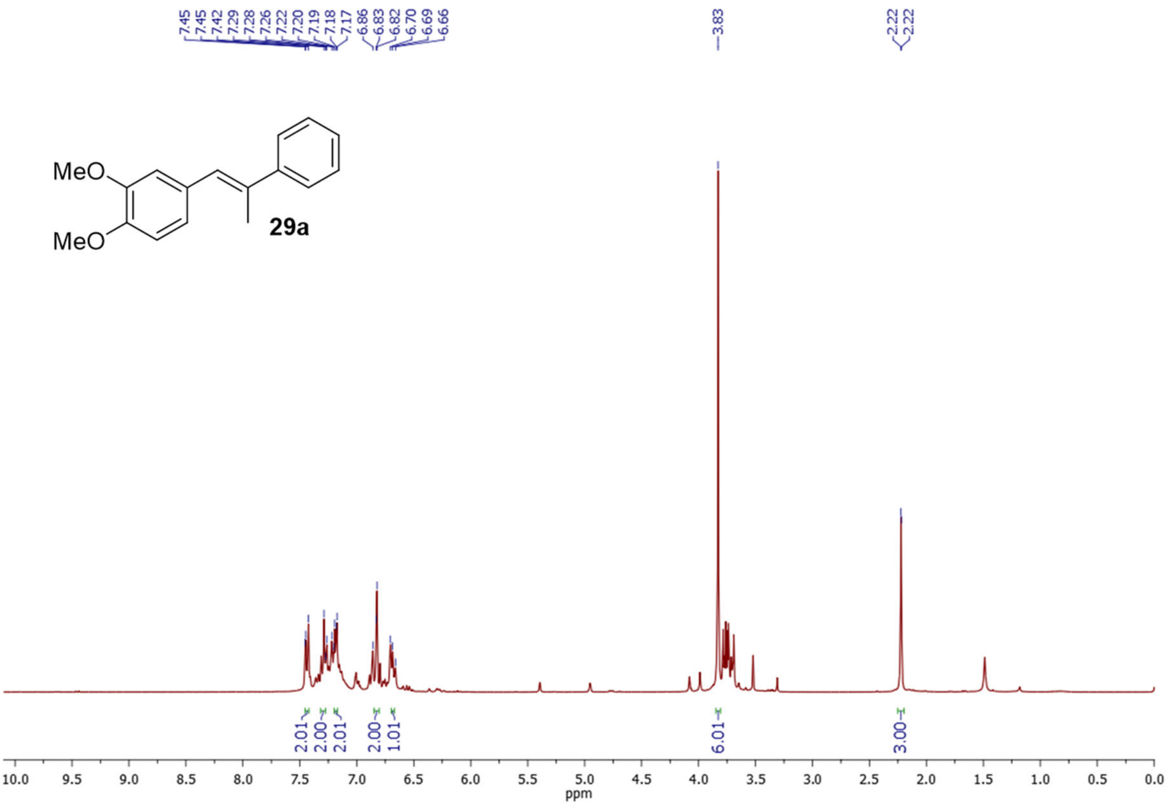

Supplementary Figure 58. ^{1}H-NMR of compound **29a**, recorded at 400 MHz and 25 ºC in $CDCl_3$.

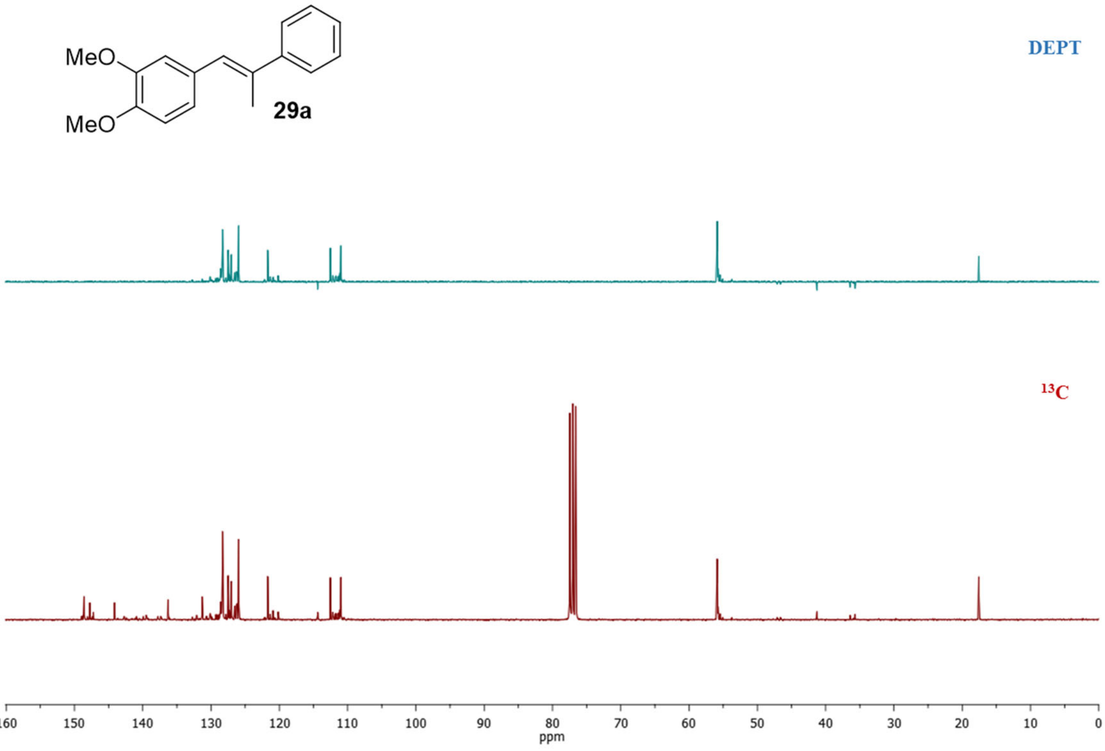

Supplementary Figure 59. ^{13}C-NMR and DEPT of compound **29a**, recorded at 100 MHz and 25 ºC in $CDCl_3$.

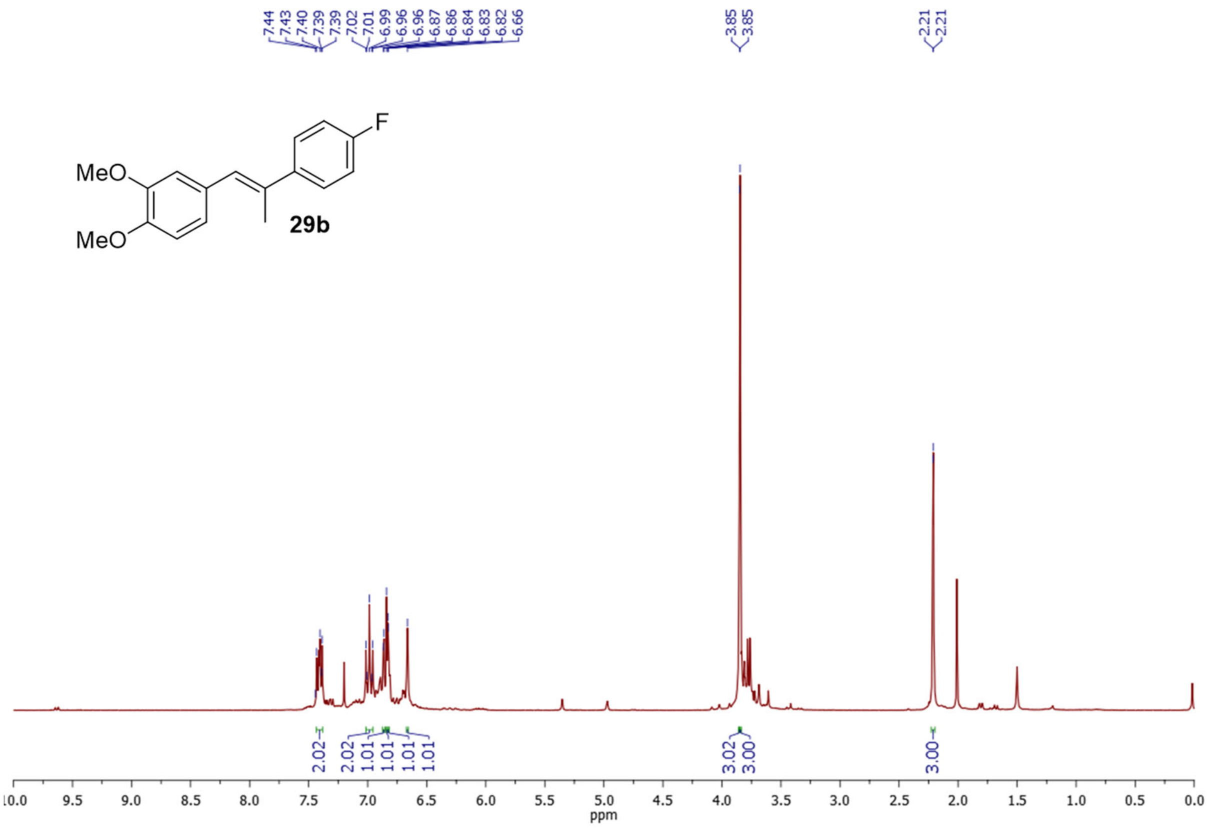

Supplementary Figure 60. ^{1}H-NMR of compound **29b**, recorded at 400 MHz and 25 ºC in $CDCl_3$.

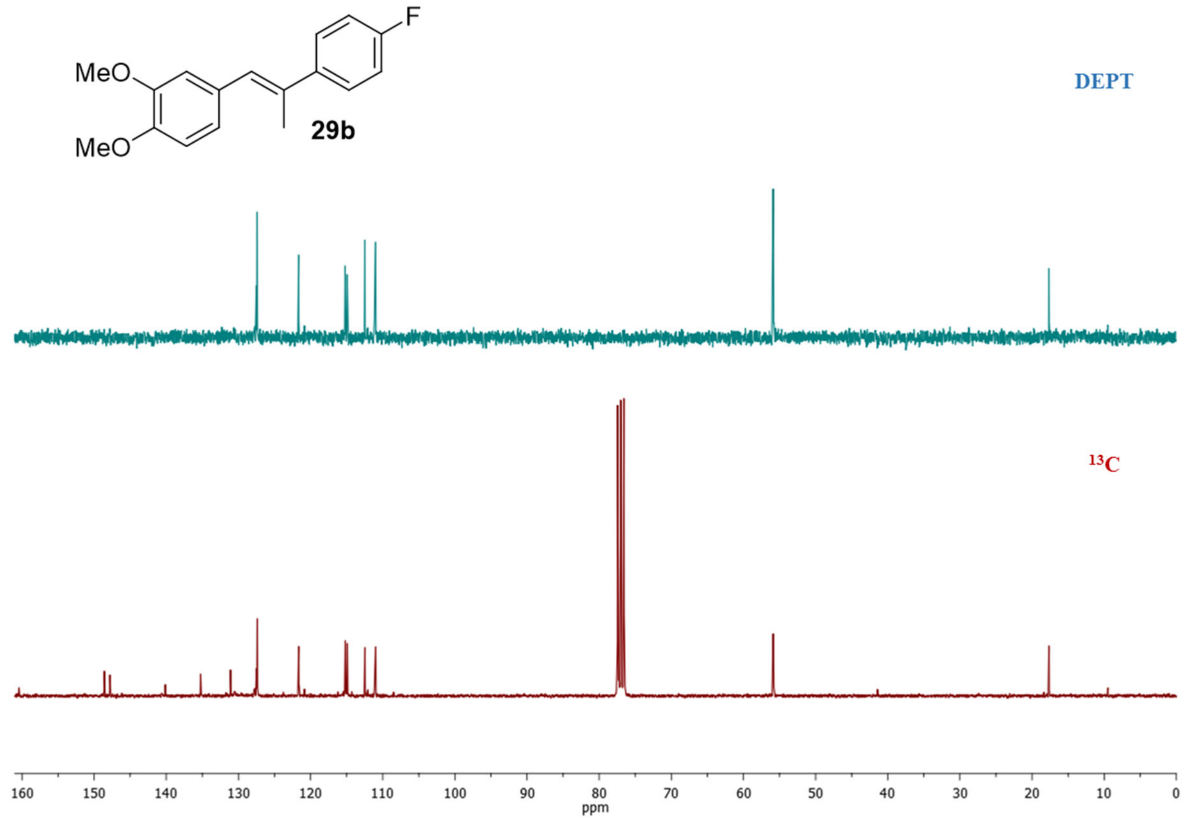

Supplementary Figure 61. ^{13}C-NMRand DEPT of compound **29b**, recorded at 100 MHz and 25 ºC in $CDCl_3$.

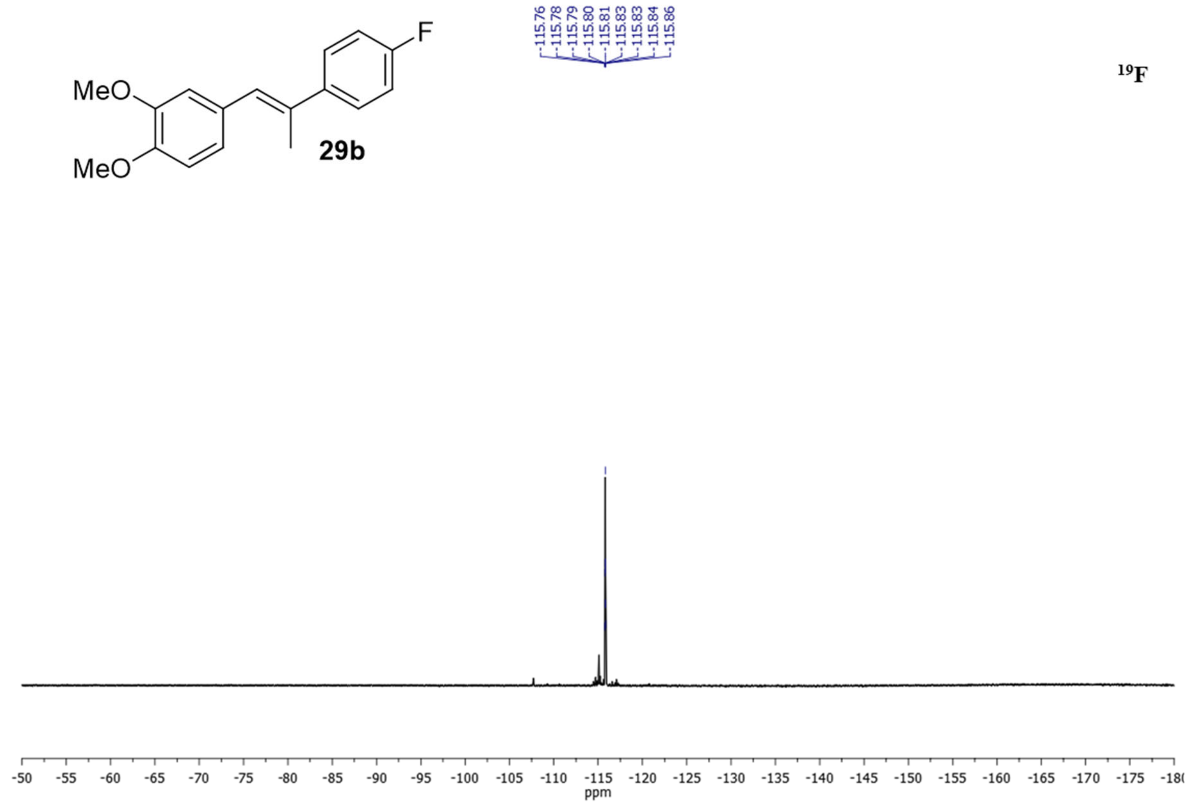

Supplementary Figure 62. ^{19}F-NMR of compound **29b**, recorded at 400 MHz and 25 ºC in $CDCl_3$.

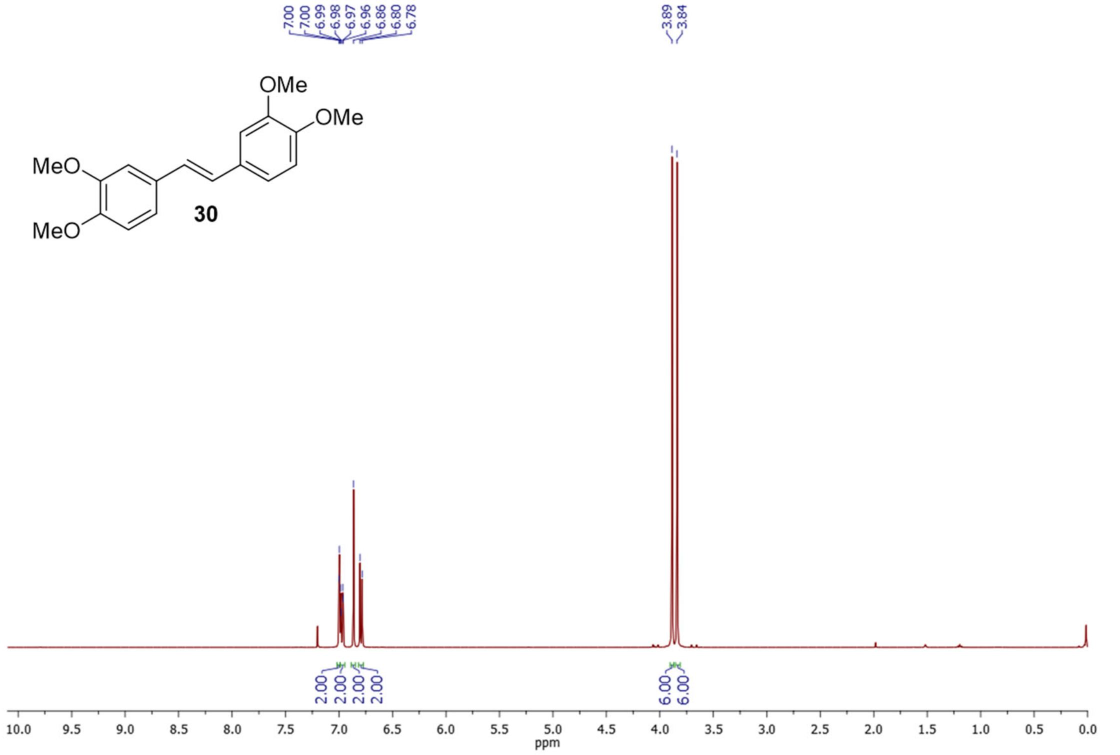

Supplementary Figure 63. ^{1}H-NMR of compound **30**, recorded at 400 MHz and 25 ºC in $CDCl_3$.

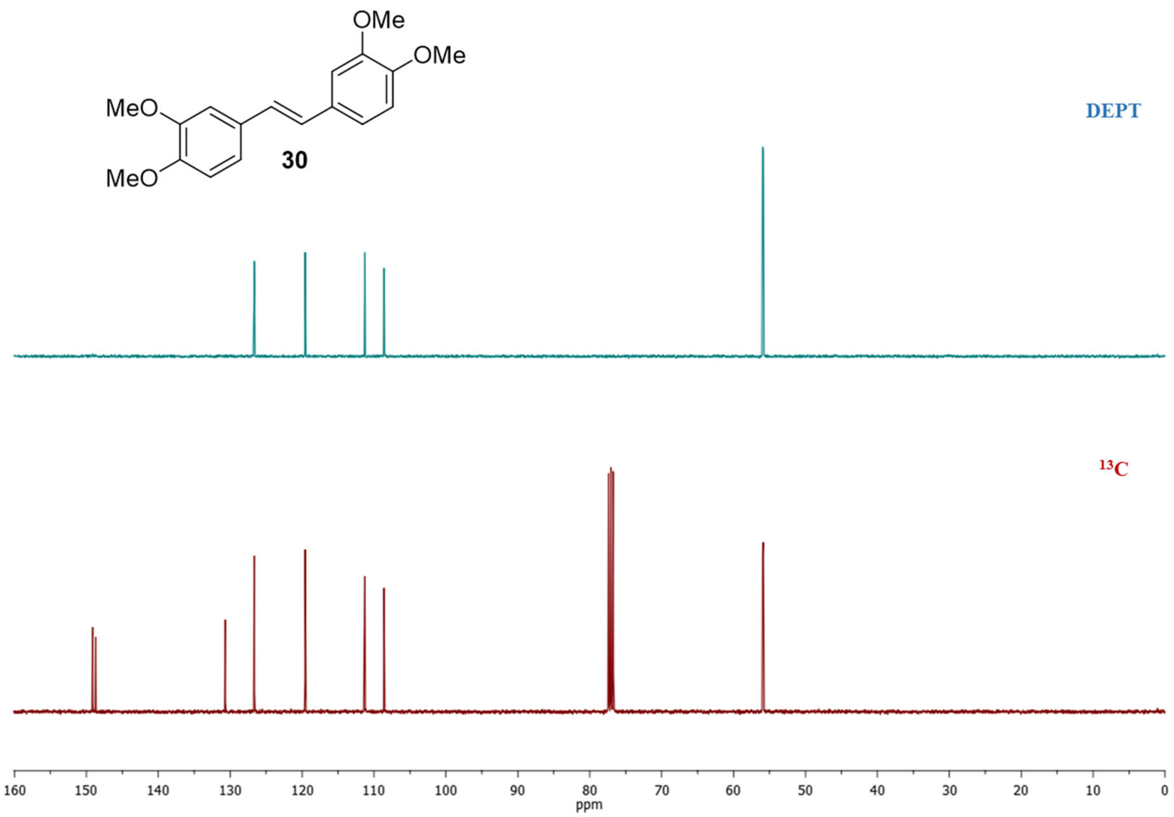

Supplementary Figure 64. ^{13}C-NMR and DEPT of compound **30**, recorded at 100 MHz and 25 ºC in $CDCl_3$.

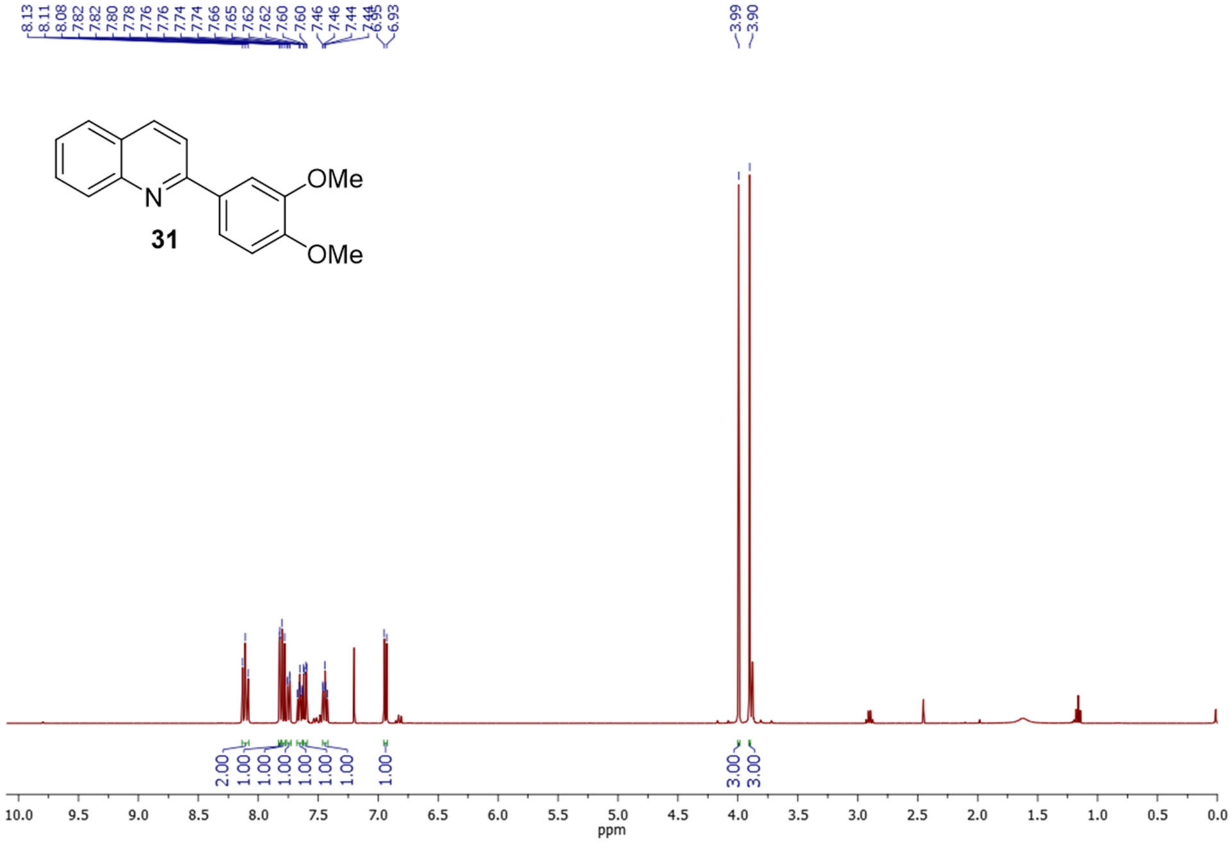

Supplementary Figure 65. ^{1}H-NMR of compound **31**, recorded at 400 MHz and 25 ºC in $CDCl_3$.

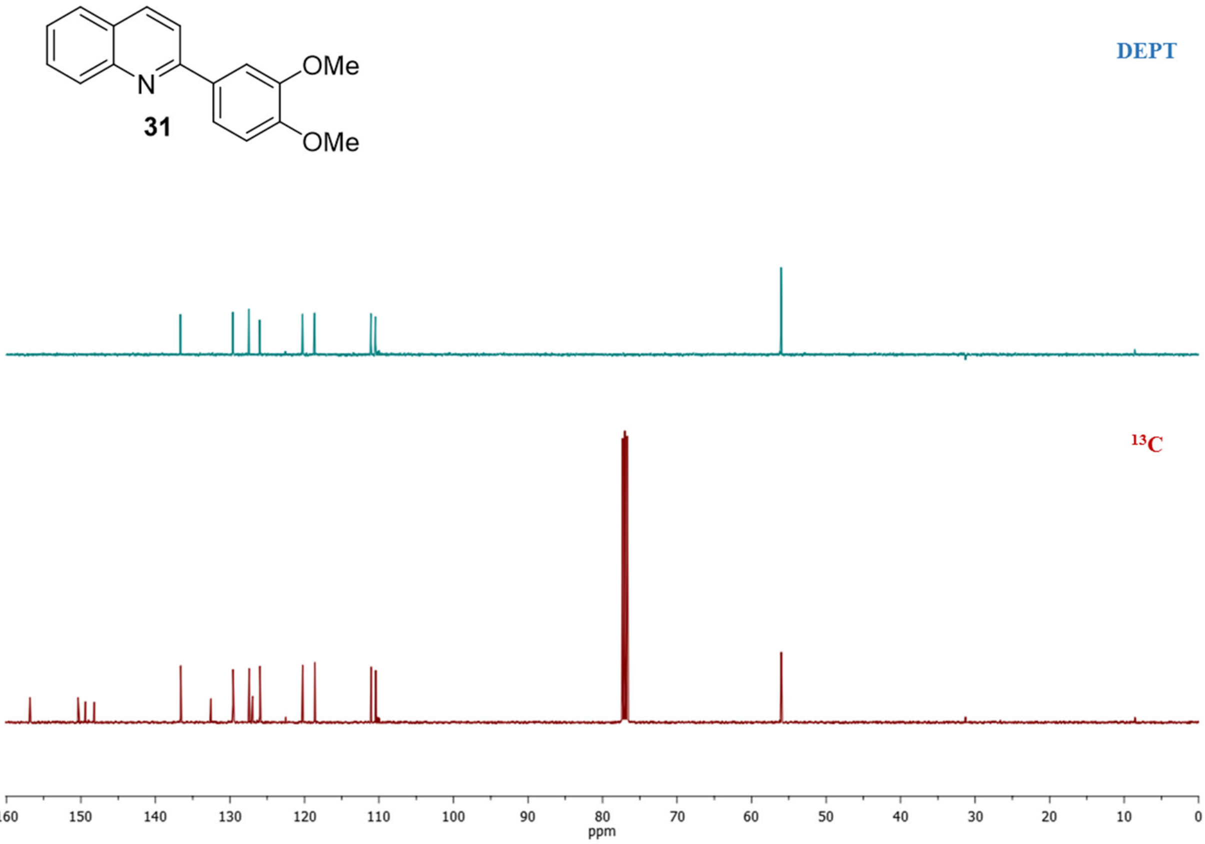

Supplementary Figure 66. ^{13}C-NMR and DEPT of compound **31**, recorded at 100 MHz and 25 ºC in $CDCl_3$.

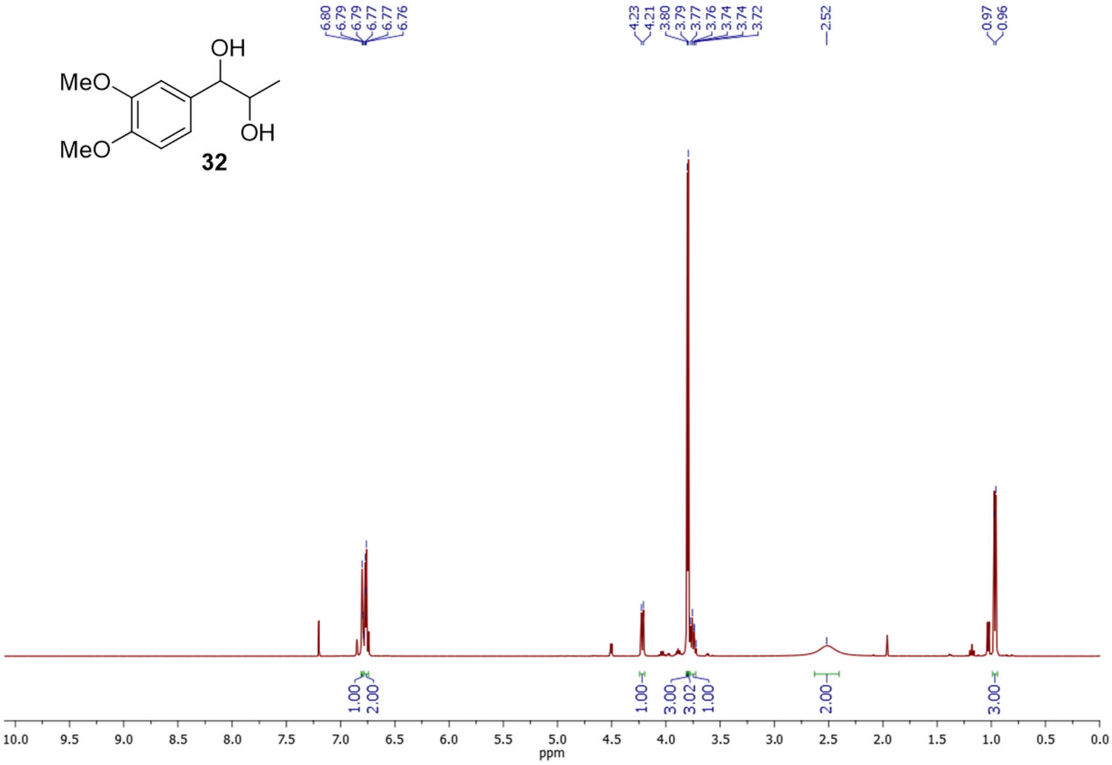

Supplementary Figure 67. ^{1}H-NMR of compound **32**, recorded at 400 MHz and 25 ºC in $CDCl_3$.

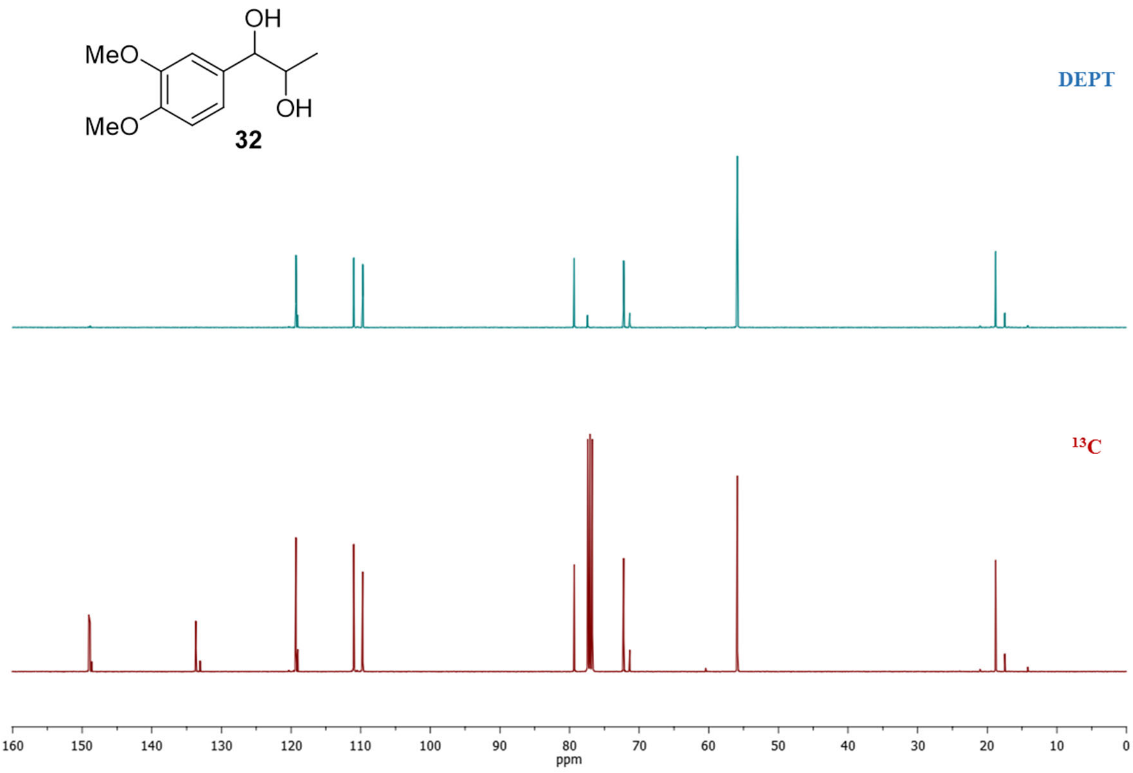

Supplementary Figure 68. ^{13}C-NMR and DEPT of compound **32**, recorded at 100 MHz and 25 ºC in $CDCl_3$.

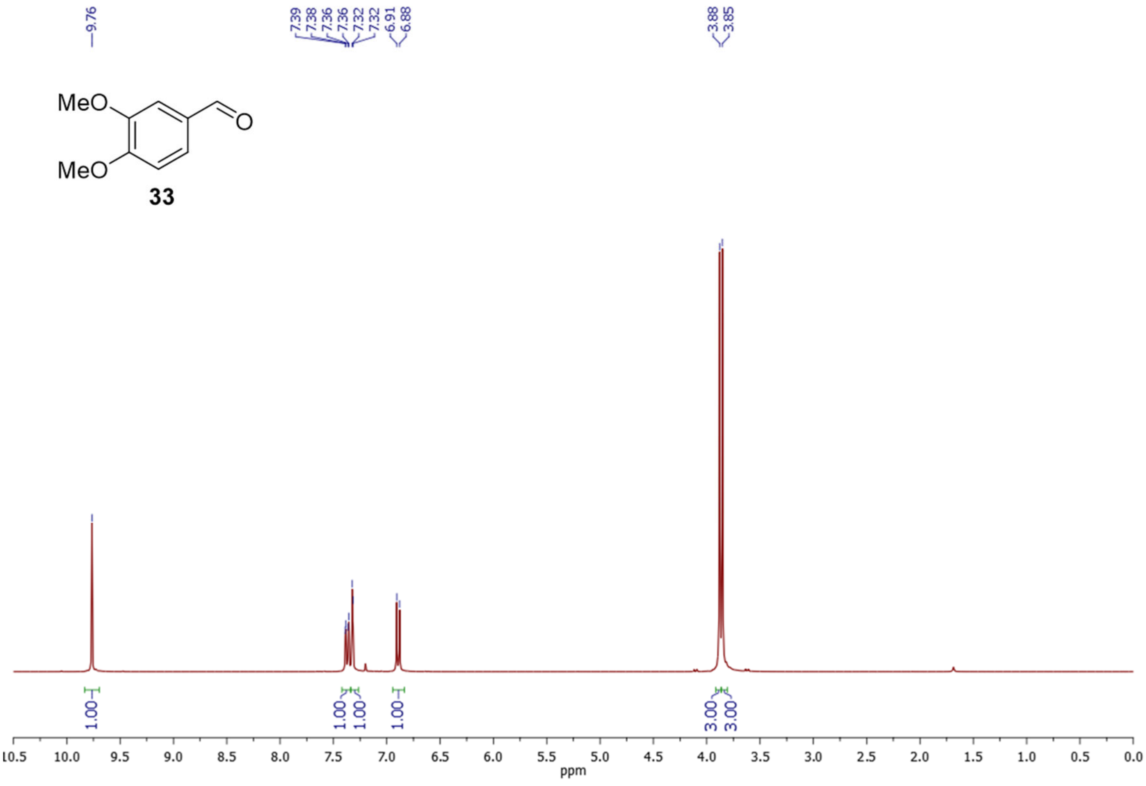

Supplementary Figure 69. ^{1}H-NMR of compound **33**, recorded at 400 MHz and 25 ºC in $CDCl_3$.

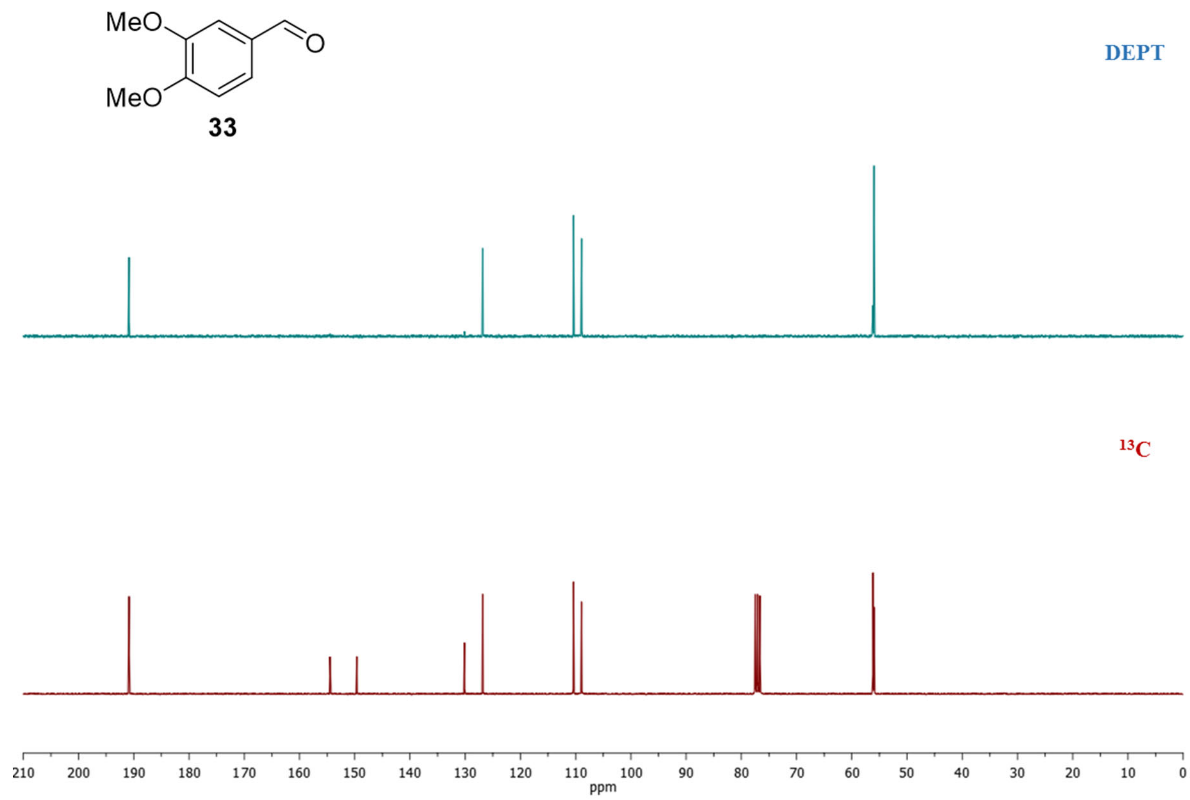

Supplementary Figure 70. ^{13}C-NMR and DEPT of compound **33**, recorded at 100 MHz and 25 ºC in $CDCl_3$.

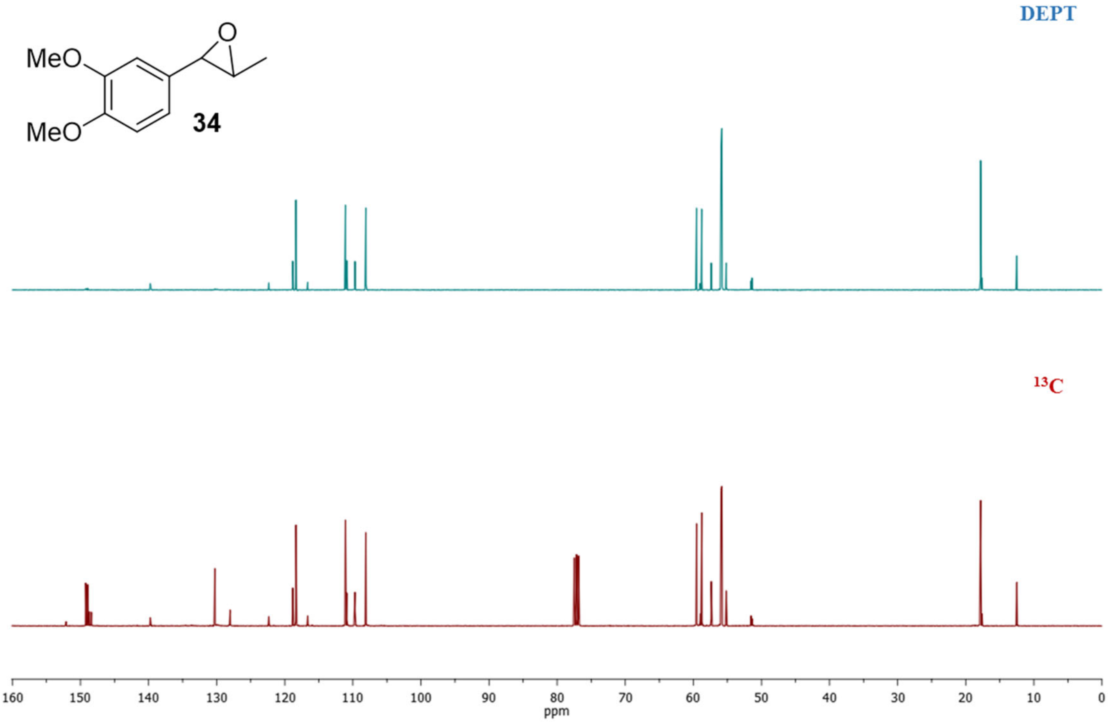

Supplementary Figure 71. ^{1}H-NMR of compound **34**, recorded at 400 MHz and 25 ºC in $CDCl_3$.

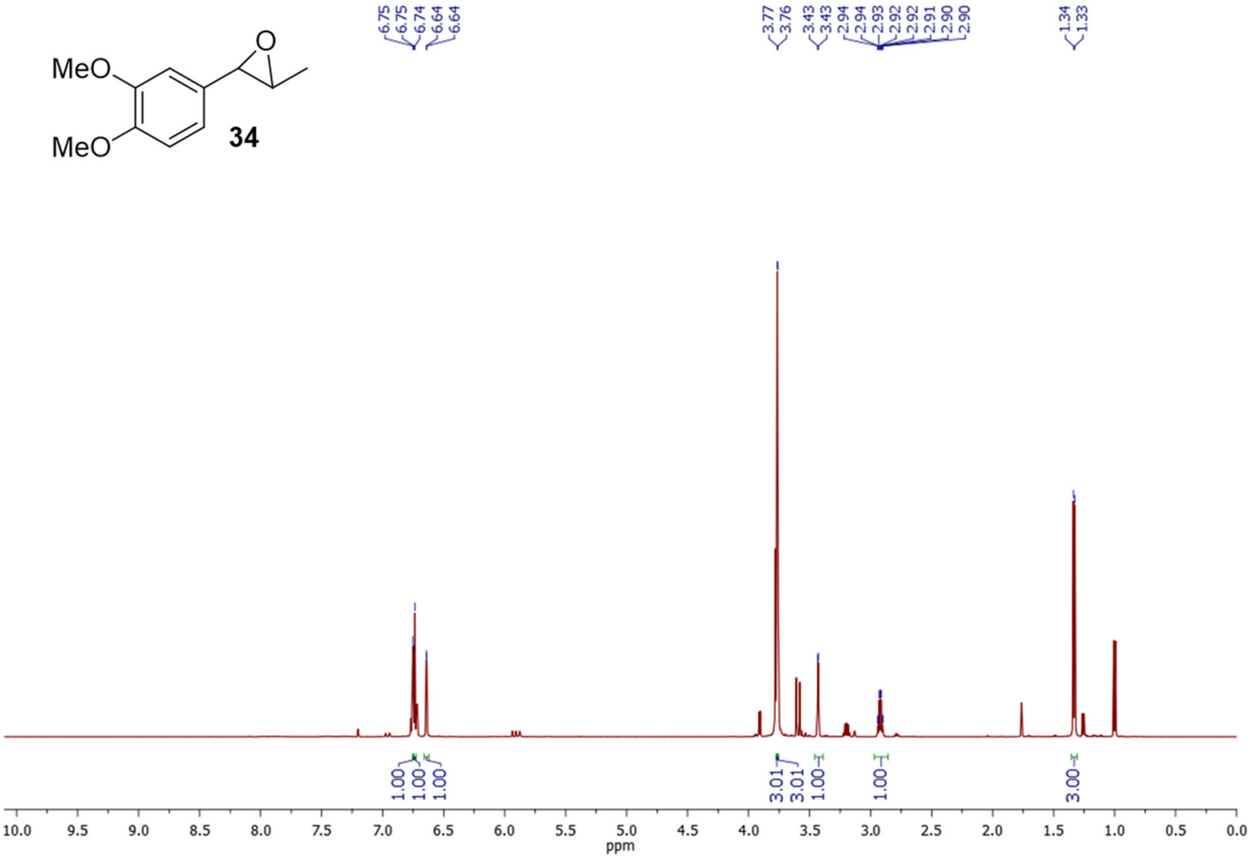

Supplementary Figure 72. ^{13}C-NMR and DEPT of compound **34**, recorded at 100 MHz and 25 ºC in $CDCl_3$.

- Supplementary Tables.

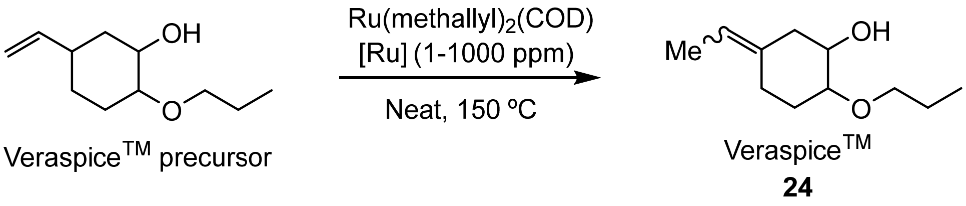

Entry	Ru (ppm)	Time (h)	Conv. (%)	Isomer 1	Isomer 2
**1**	1000	1	97.8	61.5	36.3
**2**	**500**	**3**	**98.2**	**62.1**	**36.1**
**3**	100	21	89.5	56.1	33.4
**4**	50	21	70.5	43.4	27.1
**5**	10	64	25.0	14.6	10.4
**6**	5	64	15.1	8.7	6.4
**7**	1	64	4.1	2.3	1.8
**8**	**Veraspice™ composition**			**64.7**	**34.8**

Supplementary Table S1. GC results for the synthesis of Veraspice™ catalyzed by different amounts of Ru(methylallyl)$_2$(COD) at 150 ºC and different reaction times. For the sake of comparison, the Veraspice™ composition (entry 8) is also provided.

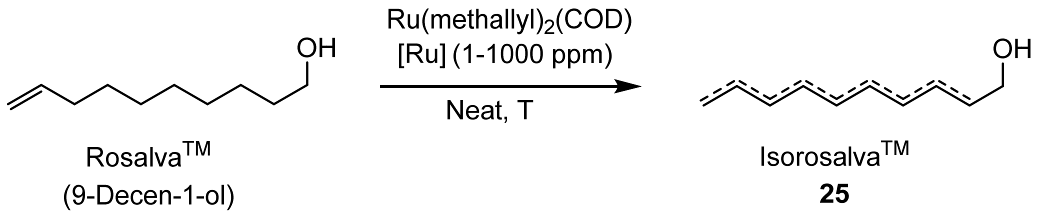

150 ˚C

Entry	Ru (ppm)	t (h)	Conv. (%)	Isomer 1	Isomer 2	Isomer 3	Isomer 4	Isomer 5	Isomer 6
**1**	1000	1	97	3.0	5.8	27.6	27.6	23.3	9.7
**2**	**500**	**2**	**97**	**2.8**	**3.8**	**21.9**	**30.4**	**27.0**	**11.1**
**3**	**100**	**3**	**96**	**0.5**	**1.5**	**21.8**	**28.5**	**29.5**	**14.2**
**4**	50	4	96	0.8	1.0	16.3	27.5	34.0	16.4
**5**	10	22	95	0.5	0.2	8.2	27.9	40.0	18.2
**6**	5	22	95	0.5	0.0	5.0	21.0	47.5	21.0
**7**	1	22	91	0.5	0.0	4.1	12.3	51.0	23.1
**8**	**Isorosalva™ composition**			**1.3**	**5.7**	**18.9**	**30.9**	**29.4**	**10.3**

175 ˚C

Entry	Ru (ppm)	t (h)	Conv. (%)	Isomer 1	Isomer 2	Isomer 3	Isomer 4	Isomer 5	Isomer 6
**1**	100	0.5	96	1.5	7.6	31.6	27.9	19.2	8.2
**2**	50	0.5	96	1.1	4.9	28.0	29.6	22.7	9.7
**3**	**10**	**1**	**96**	**0.4**	**1.7**	**19.5**	**32.4**	**30.2**	**11.8**
**4**	5	1	95	0.4	0.8	10.4	33.7	35.0	14.7
**5**	1	22	94	0.4	0.0	5.5	13.5	50.1	24.5
**6**	**Isorosalva™ composition**			**1.3**	**5.7**	**18.9**	**30.9**	**29.4**	**10.3**

200 ˚C

Entry	Ru (ppm)	t (h)	Conv. (%)	Isomer 1	Isomer 2	Isomer 3	Isomer 4	Isomer 5	Isomer 6
**1**	100	0.5	98	1.8	8.4	34.5	27.8	17.7	7.8
**2**	50	0.5	96	1.3	6.4	28.0	30.1	20.6	9.6
**3**	**10**	**0.5**	**96**	**0.8**	**3.3**	**24.2**	**30.2**	**26.0**	**11.5**
**4**	**5**	**1**	**96**	**0.7**	**2.2**	**21.6**	**30.3**	**28.7**	**12.5**
**5**	1	22	94	0.5	1.0	6.0	15.1	51.0	20.4
**6**	**Isorosalva™ composition**			**1.3**	**5.7**	**18.9**	**30.9**	**29.4**	**10.3**

Supplementary Table S2. GC results for the isomerization of Rosalva to Isorosalva **25** with ppm of Ru(methylallyl)$_2$(COD) at different reaction temperatures under solventless conditions. For the sake of comparison, the Isorosalva™ composition (entry 6) is also provided.

3alkenes-Ru(II)

C	0.000000	0.000000	0.000000
Ru	0.000000	0.000000	2.198786
C	1.777657	0.000000	3.328620
C	1.702524	1.326730	2.901048
C	0.637587	1.242814	-0.007801
C	2.032220	1.466241	-0.438920
C	1.958575	1.864268	-1.955778
C	2.230683	-1.132017	2.475703
C	2.671512	-2.404598	3.203238
C	-1.448996	1.961689	2.335180
C	-1.944196	0.868871	3.007643
C	-2.992871	-0.074699	2.478562
C	-2.418449	-1.496160	2.351632
H	-1.078323	-0.031072	-0.147336
H	0.539199	-0.869786	-0.365708
H	0.028658	2.134288	0.103273
H	2.496402	2.301437	0.090662
H	2.651101	0.572624	-0.338380
H	2.978678	2.068467	-2.285987
H	1.550008	1.055888	-2.562242
H	1.363208	2.765506	-2.107155
H	1.378223	2.085575	3.603775
H	2.278652	1.677368	2.058361
H	1.473662	-0.244225	4.350750
H	1.292844	-1.449562	1.845663
H	2.932498	-0.813502	1.702858
H	2.851921	-3.213472	2.495844
H	3.606559	-2.201900	3.731228
H	1.929764	-2.736611	3.932015
H	-0.867499	2.722570	2.839660
H	-1.825417	2.206296	1.347322
H	-1.690503	0.759465	4.068376
H	-3.843953	-0.082923	3.166501
H	-3.367719	0.277583	1.514365
H	-3.138991	-2.177218	1.892614
H	-1.539734	-1.555124	1.662993
H	-2.122212	-1.921521	3.314502

3alkenes-Ru(II) complex formation

C	-0.633033	-1.082765	-1.386109
C	-1.261366	-1.410018	-0.174638
C	-2.710311	-1.100044	0.063681
C	-3.551543	-2.221506	-0.608802
Ru	0.353629	0.097619	0.064908
C	2.250304	-0.758397	0.854293
C	3.110520	-1.128100	-0.318887
C	3.110255	-0.043793	-1.415498
C	-0.420911	1.844693	0.975038
C	-1.118662	2.252277	-0.279263
C	-0.878579	3.699163	-0.733908
C	-0.841709	0.819780	1.831679
C	1.245527	-1.537299	1.407972
H	0.222441	-1.663305	-1.741004
H	-1.143327	-0.500684	-2.148345
H	-0.911722	-2.283644	0.361726
H	-2.946858	-1.099481	1.129271
H	-3.001447	-0.137523	-0.364574
H	-4.607587	-2.019390	-0.420469
H	-3.396755	-2.251753	-1.688754
H	-3.316097	-3.202411	-0.192336
H	-0.303544	0.654014	2.758551
H	-1.848699	0.432957	1.792189
H	0.471224	2.419400	1.245118
H	-0.694419	1.585878	-1.110244
H	-2.176888	1.987193	-0.248499
H	-1.348733	3.875764	-1.700833
H	-1.326059	4.381536	-0.007799
H	0.184994	3.929787	-0.818018
H	0.878947	-1.361830	2.411509
H	1.050336	-2.521722	0.994737
H	2.567486	0.137092	1.405111
H	4.132497	-1.248792	0.063259
H	2.813706	-2.101036	-0.718891

H	3.741242	-0.351679	-2.251130
H	2.109552	0.126828	-1.852147
H	3.487101	0.913645	-1.047736

3alkenes-Ru(II) H elimination

C	0.000000	0.000000	0.000000
Ru	0.000000	0.000000	2.185830
C	2.344778	0.000000	2.230415
C	1.833776	1.046553	2.971618
C	1.699874	2.468257	2.499474
C	0.232400	2.937142	2.521177
C	0.432553	-1.318390	0.201561
C	-0.390785	-2.535434	-0.037341
C	-0.031860	-3.080366	-1.451443
C	-2.319020	-0.124092	2.751077
C	-1.477410	-1.200560	3.184234
C	-3.070842	-0.183101	1.593012
C	-4.017244	0.840688	1.136068
H	0.729986	0.770009	-0.237904
H	-0.998153	0.184654	-0.381294
H	1.500542	-1.498958	0.262141
H	-0.161740	-3.313431	0.695532
H	-1.459509	-2.316250	0.002643
H	-0.622888	-3.979926	-1.631044
H	-0.262029	-2.354765	-2.232959
H	1.023090	-3.350724	-1.519322
H	-1.161924	-1.208193	4.228425
H	-1.599304	-2.188721	2.754216
H	-2.451469	0.735516	3.404953
H	0.495215	-1.442758	2.398583
H	-3.004953	-1.091209	0.995870
H	-3.881450	1.063962	0.070260
H	-5.028557	0.401880	1.185505
H	-4.003017	1.754900	1.727741
H	2.666162	-0.916378	2.711120
H	2.722497	0.180143	1.229223
H	1.651379	0.873836	4.038466
H	2.290902	3.101765	3.170136
H	2.127992	2.580069	1.500622
H	0.153043	3.960326	2.148177
H	-0.419215	2.347983	1.843061
H	-0.195861	2.917396	3.526659

3alkenes-Ru(II) isomerization

C	0.005077	0.000189	-0.002221
C	-0.001253	0.006776	1.392696
C	1.204522	-0.000815	2.257081
C	1.498404	1.489891	2.628789
Ru	-0.750417	-2.102346	0.240536
C	0.463745	-3.273294	1.904105
C	0.843701	-3.588249	0.508948
C	1.650985	-2.777540	-0.261806
C	2.069606	-3.088004	-1.656128
C	-2.879942	-1.121184	1.017209
C	-3.084246	-1.972376	-0.028440
C	-3.134465	-1.566244	-1.477673
C	-1.832013	-2.045902	-2.124989
H	-0.813065	0.475453	-0.533455
H	0.952208	-0.011848	-0.530849
H	-0.919125	0.290077	1.896872
H	1.026467	-0.541374	3.189976
H	2.080350	-0.416572	1.755506
H	2.370783	1.502778	3.284471
H	1.720283	2.086791	1.743656
H	0.663053	1.942286	3.165059
H	0.058482	-4.120057	2.458866
H	1.200665	-2.720759	2.482833
H	0.479013	-4.524428	0.084357
H	-0.489976	-2.521301	1.974525
H	2.171780	-1.960676	0.228443
H	2.067796	-2.199870	-2.293129
H	3.114346	-3.426908	-1.624452
H	1.475481	-3.877012	-2.118432

H	-2.995242	-1.456034	2.042036
H	-2.847385	-0.049642	0.852412
H	-3.299085	-3.022239	0.191400
H	-3.991056	-2.022053	-1.981409
H	-3.240069	-0.483610	-1.573679
H	-1.674642	-1.634768	-3.126889
H	-0.891530	-1.625445	-1.621034
H	-1.745453	-3.133826	-2.188402

4alkenes-Ru(II)

C	-0.752212	0.385865	0.312113
C	-0.231417	0.220737	1.576346
C	1.190931	0.424619	1.975322
C	1.295147	1.757832	2.763595
Ru	-0.849310	-1.899486	0.151873
C	1.310907	-2.005066	-0.574726
C	0.882173	-3.226192	-0.073494
C	-0.071426	-4.096540	-0.814577
C	-0.153669	-5.559697	-0.382129
C	-0.629144	-2.560876	2.512854
C	-1.753290	-3.011388	1.853107
C	-2.957169	-2.165492	1.599419
C	-4.282032	-2.915933	1.433263
C	-2.060648	-1.361204	-1.936164
C	-0.959910	-1.033542	-2.676903
C	-0.571737	0.333971	-3.119071
C	0.932547	0.609208	-3.255129
H	-1.800626	0.641681	0.201675
H	-0.097813	0.687775	-0.495549
H	-0.944645	0.174728	2.394598
H	1.541478	-0.384428	2.622943
H	1.839255	0.470077	1.098759
H	2.335562	1.913587	3.052832
H	0.979100	2.606902	2.155714
H	0.692401	1.735746	3.673668
H	2.002988	-1.414408	0.010745
H	1.284434	-1.791561	-1.631634
H	1.283133	-3.593020	0.863573
H	-1.146643	-3.649866	-0.650583
H	0.036733	-3.988905	-1.896350
H	-0.976762	-6.073481	-0.878748
H	0.773987	-6.061887	-0.668012
H	-0.270774	-5.669530	0.697842
H	0.190671	-3.238892	2.712664
H	-0.643481	-1.662583	3.107783
H	-1.820634	-4.060542	1.578029
H	-3.032132	-1.345544	2.317255
H	-2.788176	-1.612709	0.595393
H	-5.082164	-2.235027	1.143333
H	-4.210058	-3.707521	0.684723
H	-4.558287	-3.370790	2.387171
H	-2.469943	-2.363875	-2.004376
H	-2.756286	-0.582278	-1.639382
H	-0.389380	-1.847427	-3.122686
H	-1.019456	0.402766	-4.125714
H	-1.084270	1.101598	-2.532996
H	1.091017	1.575067	-3.735177
H	1.438316	0.640976	-2.286412
H	1.423107	-0.146157	-3.874428

4alkenes-Ru(II) complex formation

C	0.011862	-0.010726	0.000504
C	-0.004499	0.001056	1.377615
C	1.189116	0.008016	2.271524
C	1.352116	1.433243	2.864660
Ru	-0.610872	-2.215165	0.102265
C	-2.307703	-2.742905	1.440192
C	-3.083873	-1.755387	0.633018
C	-4.412836	-2.254775	0.058138
C	-0.831842	-1.912732	-2.342391
C	0.503182	-1.968993	-2.629177
C	1.361486	-0.828420	-3.051937
C	2.834706	-0.883425	-2.623276

C	1.561085	-2.886772	0.296166
C	0.683770	-3.866949	0.738652
C	-0.140696	-4.675511	-0.200810
C	-0.744582	-5.968842	0.344353
C	-1.409773	-2.386659	2.424427
H	-0.827120	0.412373	-0.541622
H	0.961825	-0.019975	-0.518339
H	-0.944926	0.268960	1.851099
H	1.068619	-0.696048	3.100153
H	2.091356	-0.260340	1.719526
H	2.227908	1.444069	3.515238
H	1.500036	2.177508	2.080751
H	0.485271	1.722328	3.462185
H	2.132107	-2.329028	1.026560
H	1.962357	-2.902873	-0.704867
H	0.621869	-4.095166	1.795782
H	-1.050583	-4.005952	-0.526758
H	0.363037	-4.824142	-1.158768
H	-1.444795	-6.408754	-0.365764
H	0.060740	-6.689245	0.508499
H	-1.256335	-5.820959	1.297429
H	-0.917041	-3.151056	3.011494
H	-1.398356	-1.396435	2.849174
H	-2.543623	-3.793593	1.294186
H	-3.190178	-0.802194	1.155845
H	-2.437364	-1.473063	-0.286020
H	-4.855468	-1.510678	-0.603905
H	-4.291120	-3.186781	-0.497428
H	-5.113041	-2.433362	0.877267
H	-1.436104	-2.808869	-2.439031
H	-1.360256	-0.969426	-2.440734
H	0.962027	-2.953547	-2.711267
H	1.328325	-0.891418	-4.153498
H	0.893846	0.130663	-2.813403
H	3.395437	-0.091528	-3.120187
H	2.959492	-0.743204	-1.546248
H	3.297329	-1.833679	-2.902117

4alkenes-Ru(II) H-elimination

C	1.558484	-1.737490	0.644076
Ru	-0.326201	-0.546614	0.134001
C	-1.897294	-1.926614	0.939883
C	-3.024685	-1.412767	1.769865
C	1.884734	-1.592317	-0.693479
C	2.774203	-0.543151	-1.248576
C	4.170850	-1.183390	-1.527859
C	0.074119	0.599728	2.247396
C	0.708396	1.713362	1.771628
C	2.167098	2.004146	1.826028
C	2.691683	2.951872	0.738882
C	-0.743955	0.519822	-1.593761
C	-1.871926	1.137316	-0.845475
C	-1.901239	2.677452	-0.745079
C	-2.347077	3.300694	-2.074081
C	-0.847558	-2.457190	-1.185967
C	-1.936854	-1.961956	-0.483511
H	1.193323	-2.693532	1.008365
H	2.073293	-1.124474	1.374682
H	1.622078	-2.383318	-1.386915
H	2.384164	-0.173431	-2.201444
H	2.891994	0.294260	-0.558570
H	4.818106	-0.417393	-1.957196
H	4.628591	-1.552609	-0.609563
H	4.096263	-2.005212	-2.241598
H	-0.975495	-0.048255	-2.488462
H	0.151702	1.137542	-1.705499
H	-2.838360	0.745284	-1.167458
H	-2.588070	2.971950	0.052164
H	-0.907746	3.047149	-0.477083
H	-2.379299	4.387411	-1.977492
H	-1.660463	3.058161	-2.889260
H	-3.347973	2.966510	-2.357425
H	-0.804132	-2.369206	-2.264713
H	-0.228198	-3.224012	-0.740829
H	-2.754588	-1.477309	-1.006071

H	-1.241165	-2.642817	1.433151
H	-1.804941	0.756854	0.254610
H	-2.700148	-1.077786	2.755833
H	-3.607569	-0.630882	1.280335
H	-3.706295	-2.258506	1.948717
H	-0.986050	0.664604	2.469136
H	0.634490	-0.155215	2.790856
H	0.083881	2.533259	1.420742
H	2.297280	2.499712	2.803401
H	2.756608	1.085246	1.903744
H	3.747485	3.167327	0.905387
H	2.593074	2.528273	-0.264050
H	2.157140	3.904339	0.756638

4alkenes-Ru(II) isomerization

C	0.325728	1.941569	-1.268511
Ru	-0.329227	-0.052062	0.004929
C	-2.027772	1.809932	0.857123
C	-3.397185	2.003858	0.310507
C	1.152236	2.192659	-0.210000
C	2.642533	2.078819	-0.210817
C	3.249637	3.494057	-0.402746
C	-1.359024	-2.115707	1.024434
C	-1.424938	-2.255532	-0.326804
C	-2.541113	-1.761506	-1.200180
C	-1.961618	-0.724007	-2.170461
C	1.318282	-1.088283	-1.314663
C	1.875435	-1.249105	-0.066232
C	1.994285	-2.543272	0.678470
C	3.432619	-3.091207	0.493943
C	-0.320347	0.638473	2.290913
C	-1.668713	0.786109	1.697875
H	-0.648857	2.407148	-1.337419
H	0.736384	1.582117	-2.205215
H	0.743912	2.740195	0.637002
H	3.002330	1.684771	0.744764
H	2.993435	1.427109	-1.013713
H	4.337646	3.418722	-0.375859
H	2.958322	3.921783	-1.363100
H	2.940032	4.176062	0.391728
H	1.634703	-0.273331	-1.950396
H	0.897290	-1.940973	-1.833154
H	2.514094	-0.452894	0.304466
H	1.821451	-2.394878	1.748456
H	1.280705	-3.283868	0.311965
H	3.530969	-4.025364	1.048921
H	3.648898	-3.292676	-0.556419
H	4.180728	-2.393600	0.875943
H	-0.295445	-0.033833	3.148553
H	0.203174	1.568155	2.506227
H	-2.445738	0.112299	2.031023
H	-1.335305	2.630406	0.706298
H	0.490649	0.072661	1.593768
H	-3.382764	2.288349	-0.745151
H	-4.042492	1.136900	0.452144
H	-3.851135	2.854990	0.836697
H	-0.576059	-2.600328	1.593427
H	-2.217046	-1.768381	1.582440
H	-0.664947	-2.850370	-0.820839
H	-2.972068	-2.590946	-1.768360
H	-3.343059	-1.330050	-0.598064
H	-2.736739	-0.117321	-2.644448
H	-1.329154	0.082623	-1.677310
H	-1.328176	-1.165894	-2.938367

Supplementary Table S3. Computational data with atomic coordinates.

- Compound characterization.

Starting material: methyl eugenol[1]

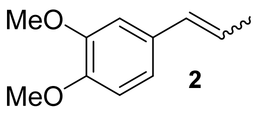

**Composition**	$C_{11}H_{14}O_2$
**Molecular weight (g/mol)**	178.23
**Concentration cat. (mol%)**	0.001
**Temperature (ºC)**	150
**Yield (%) (*cis/trans*):**	97.2 (15.5/81.7)

^{1}H-RMN (401 MHz, $CDCl_3$,) δ (ppm):

6.78 (s, 1H), 6.72 (d, J = 1.5 Hz, 1H), 6.65 (d, J = 7.9 Hz, 1H), 6.22 (d, J = 15.8 Hz, 1H), 6.03 – 5.94 (m, 1H), 3.75 (s, 3H), 3.72 (s, 3H), 1.75 (d, J = 6.4 Hz, 3H).

^{13}C-RMN (101 MHz, $CDCl_3$) δ (ppm):

148.98, 148.17, 130.69, 118.66, 111.20, 108.52, 55.78, 18.32.

IR υ (cm^{-1})

1262.18, 1225.54 (Ar-OMe), 960.12 (=C-H).

Starting material: eugenol[2]

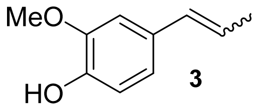

**Composition**	$C_{10}H_{12}O_2$
**Molecular weight (g/mol)**	164.20
**Concentration cat. (mol%)**	0.005
**Temperature (ºC)**	150
**Yield (%) (*cis/trans*):**	98.2 (11.3/86.9)

^{1}H-RMN (401 MHz, DMSO) δ (ppm)

8.91 (s, 1H), 6.94 (d, J = 1.8 Hz, 1H), 6.74 (dd, J = 8.1, 1.8 Hz, 1H), 6.69 (d, J = 8.1 Hz, 1H), 6.28 (dd, J = 15.8, 1.6 Hz, 1H), 6.08 (dq, J = 15.7, 6.6 Hz, 1H), 3.77 (s, 3H), 1.80 (dd, J = 6.6, 1.6 Hz, 3H).

^{13}C-RMN (101 MHz, DMSO) δ (ppm)

148.12, 146.25, 131.37, 129.56, 122.51, 119.27, 115.87, 109.82, 55.98, 18.59.

IR υ (cm^{-1})

3509.81 (Ar-OH), 1203.66 (Ar-OMe), 962.43 (=C-H)

Starting material: 4-allylanisole[1]

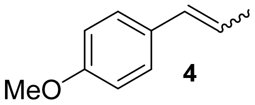 4	**Composition**	$C_{10}H_{12}O$
	**Molecular weight (g/mol)**	148.20
	**Concentration cat. (mol%)**	0.0005
	**Temperature (ºC)**	150
	**Yield (%) (*cis/trans*):**	99.7 (11.4/88.3)

**^{1}H-RMN (401 MHz, DMSO) δ (ppm)**
7.29 (d, J = 8.8 Hz, 2H), 6.86 (d, J = 8.8 Hz, 2H), 6.33 (dd, J = 15.8, 1.6 Hz, 1H), 6.11 (dq, J = 15.8, 6.6 Hz, 1H), 3.73 (s, 3H), 1.80 (dd, J = 6.6, 1.6 Hz, 3H).
**^{13}C-RMN (101 MHz, DMSO) δ (ppm)**
158.74, 130.74, 130.57, 127.28, 123.37, 114.38, 55.47, 18.64.
**IR υ (cm^{-1})**
1242.90 (Ar-OMe), 963.26 (=C-H)

Starting material: allyl phenyl ether[3]

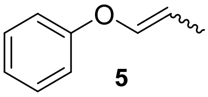 5	**Composition**	$C_9H_{10}O$
	**Molecular weight (g/mol)**	134.18
	**Concentration cat. (mol%)**	0.001
	**Temperature (ºC)**	200
	**Yield (%) (*cis/trans*):**	97.4 (65.4/32.0)

**^{1}H-RMN (401 MHz, DMSO) δ (ppm)**
7.35 – 7.28 (m, 2H), 7.07 – 6.99 (m, 3H), 6.56 (d, J = 4.4 Hz, 1H), 4.91 – 4.82 (m, 1H), 1.65 (d, J = 6.8 Hz, 3H).
**^{13}C-RMN (101 MHz, DMSO) δ (ppm)**
157.36, 141.40, 130.18, 116.30, 106.84, 9.72.
**IR υ (cm^{-1})**
1222.65 (Ar-OR), 929.52 (=C-H)

Starting material: 3-phenyl-1-propene[1]

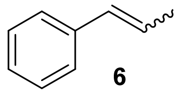 **6**	**Composition**	C_9H_{10}
	**Molecular weight (g/mol)**	118.18
	**Concentration cat. (mol%)**	0.005
	**Temperature (°C)**	150
	**Yield (%) (*cis/trans*):**	92.4 (17.7/74.7)
**^{1}H-RMN (401 MHz, DMSO) δ (ppm)**		
7.36 (d, J = 7.2 Hz, 2H), 7.29 (t, J = 7.7 Hz, 2H), 7.19 (t, J = 7.2 Hz, 1H), 6.41 (dd, J = 15.8, 1.4 Hz, 1H), 6.28 (dq, J = 15.8, 6.4 Hz, 1H), 1.84 (dd, J = 6.5, 1.5 Hz, 3H).		
**^{13}C-RMN (101 MHz, DMSO) δ (ppm)**		
137.87, 131.31, 128.95, 127.23, 126.14, 125.90, 18.69.		
**IR υ (cm^{-1})**		
961.34 (=C-H)		

Starting material: 1-allyl-4-fluorobenzene[1]

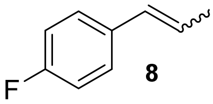 **8**	**Composition**	C_9H_9F
	**Molecular weight (g/mol)**	136.17
	**Concentration cat. (mol%)**	0.001
	**Temperature (°C)**	150
	**Yield (%) (*cis/trans*):**	98.8 (14.6/84.2)
**^{1}H-RMN (401 MHz, DMSO) δ (ppm)**		
7.40 – 7.36 (m, 2H), 7.12 – 7.08 (m, 2H), 6.38 (d, J = 15.9 Hz, 1H), 6.21 (dq, J = 15.8, 6.5 Hz, 1H), 1.81 (dd, J = 6.6, 1.6 Hz, 3H).		
**^{13}C-RMN (101 MHz, DMSO) δ (ppm)**		
161.66 (d, J = 243.4 Hz), 134.40, 130.06, 127.87 (d, J = 8.0 Hz), 125.80, 115.69 (d, J = 21.3 Hz), 18.58.		
**^{19}F-RMN (377 MHz, DMSO) δ (ppm)**		
-115.88 (s, 1F).		
**IR υ (cm^{-1})**		
958.44 (=C-H).		

Starting material: allylpentafluorobenzene[4]

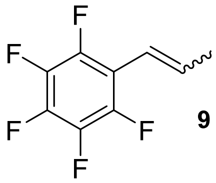

9

Composition	$C_9H_5F_5$
**Molecular weight (g/mol)**	208.13
**Concentration cat. (mol%)**	0.2
**Temperature (°C)**	150
**Yield (%) (*cis/trans*):**	97.4 (3.0/94.4)

**^{1}H-RMN (401 MHz, $CDCl_3$) δ (ppm)**
6.51 (dq, J = 16.1, 6.7 Hz, 1H), 6.23 (d, J = 16.2 Hz, 1H), 1.90 (dd, J = 6.7, 0.8 Hz, 3H).
**^{13}C-RMN (101 MHz, $CDCl_3$) δ (ppm)**
146.11 (ddd, J = 15.5, 7.9, 4.0 Hz), 142.80 (ddd, J = 11.5, 8.0, 3.9 Hz), 141.19 – 140.65 (m), 139.27 (dddd, J = 17.5, 12.8, 4.8, 2.3 Hz), 137.58 (tt, J = 13.5, 5.1 Hz), 115.29 (d, J = 2.1 Hz), 112.50 (td, J = 14.3, 4.1 Hz).
**^{19}F-RMN (377 MHz, $CDCl_3$) δ (ppm)**
-144.10 (dd, J = 21.5, 7.7 Hz, 2F), -158.11 (t, J = 20.8 Hz, 1F), -163.56 (dt, J = 21.2, 7.7 Hz, 2F).
**IR υ (cm^{-1})**
911.33 (=C-H).

Starting material: α-vinylbenzyl alcohol[5]

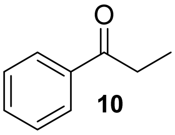

10

Composition	$C_9H_{10}O$
**Molecular weight (g/mol)**	134.18
**Concentration cat. (mol%)**	0.001
**Temperature (°C)**	150
**Yield (%) (*cis/trans*):**	98.2

**^{1}H-RMN (300 MHz, $CDCl_3$) δ (ppm)**
7.90 (d, J = 7.2 Hz, 2H), 7.48 (t, J = 7.3 Hz, 1H), 7.38 (t, J = 7.4 Hz, 2H), 2.93 (q, J = 7.2 Hz, 2H), 1.16 (t, J = 7.2 Hz, 3H).
**^{13}C-RMN (75.5 MHz, $CDCl_3$) δ (ppm)**
200.79, 136.94, 132.85, 128.54, 127.96, 31.76, 8.23.
**IR υ (cm^{-1})**
1733.69 (C=O).

Starting material: 3,4-dihydroxy-1-butene[6]

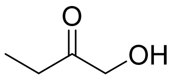 **11**	**Composition**	$C_4H_8O_2$
	**Molecular weight (g/mol)**	88.11
	**Concentration cat. (mol%)**	0.01
	**Temperature (°C)**	150
	**Yield (%)**	94.1
**^{1}H-RMN (401 MHz, $CDCl_3$) δ (ppm)**		
4.17 (s, 2H), 2.36 (q, J = 7.4 Hz, 2H), 1.06 (t, J = 7.4 Hz, 3H).		
**^{13}C-RMN (101 MHz, $CDCl_3$) δ (ppm)**		
210.29, 67.70, 31.61, 7.50.		
**IR υ (cm^{-1})**		
3423.99 (OH), 1715.32 (C=O)		

Starting material: 1,6-heptadien-4-ol[7]

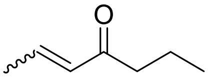 **12**	**Composition**	$C_7H_{12}O$
	**Molecular weight (g/mol)**	112.17
	**Concentration cat. (mol%)**	0.05
	**Temperature (°C)**	150
	**Yield (%) (*cis/trans*):**	99.8 (39.2/60.6)
**^{1}H-RMN (401 MHz, $CDCl_3$) δ (ppm)**		
6.75 – 6.65 (m, 1H), 6.03 – 5.90 (m, 1H), 2.26 – 2.21 (m, 2H), 1.77 – 1.74 (m, 3H), 1.52 – 1.41 (m, 2H), 0.80 – 0.74 (m, 3H).		
**^{13}C-RMN (101 MHz, $CDCl_3$) δ (ppm)**		
200.50, 142.23, 131.93, 44.61, 17.61, 17.18, 13.66.		
**IR υ (cm^{-1})**		
3436.53 (OH), 1707.65 (C=O), 971.94 (=C-H)		

Starting material: 1-octen-3-ol[8]

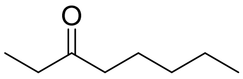 13	**Composition**	$C_8H_{16}O$
	**Molecular weight (g/mol)**	128.22
	**Concentration cat. (mol%)**	0.001
	**Temperature (°C)**	200
	**Yield (%)**	92.2
**^{1}H-RMN (401 MHz, $CDCl_3$,) δ (ppm):**		
2.28 – 2.23 (m, 4H), 1.46 – 1.39 (m, 2H), 1.18 – 1.11 (m, 4H), 0.90 (t, J = 7.3 Hz, 3H), 0.74 (t, J = 7.0 Hz, 3H).		
**^{13}C-RMN (101 MHz, $CDCl_3$) δ (ppm):**		
211.72, 42.27, 35.72, 31.37, 23.54, 22.38, 13.79, 7.70.		
**IR υ (cm^{-1})**		
1711.51 (C=O)		

Starting material: 1-methoxy-4-(1-phenylbut-3-en-1-yl)benzene[9]

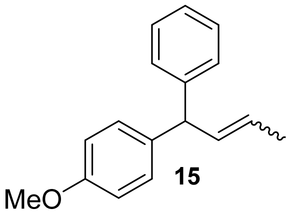 15	**Composition**	$C_{17}H_{18}O$
	**Molecular weight (g/mol)**	238.33
	**Concentration cat. (mol%)**	0.01
	**Temperature (°C)**	150
	**Yield (%) (*cis/trans*):**	95.0 (23.9/71.1)
**^{1}H-RMN (401 MHz, $CDCl_3$) δ (ppm)**		
7.40 – 7.15 (m, 7H), 6.92 (d, J = 8.5 Hz, 2H), 6.04 – 5.92 (m, 1H), 5.58 – 5.44 (m, 1H), 4.72 (d, J = 7.4 Hz, 1H), 3.85 (s, 3H), 1.83 (d, J = 0.5 Hz, 3H).		
**^{13}C-RMN (101 MHz, $CDCl_3$) δ (ppm)**		
158.04, 144.63, 136.48, 133.94, 129.49, 128.51, 128.38, 126.70, 126.16, 113.80, 55.28, 53.30, 18.06.		
**IR υ (cm^{-1})**		
1241.93 (Ar-OMe), 967.13 (=C-H)		

Starting material: allyltrimethoxysilane

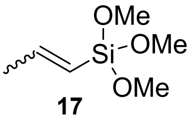 **17**	**Composition**	$C_6H_{14}O_3Si$
	**Molecular weight (g/mol)**	162.26
	**Concentration cat. (mol%)**	0.005
	**Temperature (ºC)**	150
	**Yield (%) (*cis/trans*):**	84.1 (9.6/74.5)
**^{1}H-RMN (401 MHz, $CDCl_3$) δ (ppm)**		
6.39 (dq, J = 18.8, 6.3 Hz, 1H), 5.35 (ddd, J = 18.8, 3.2, 1.6 Hz, 1H), 3.50 (s, 9H), 1.81 (dd, J = 6.3, 1.6 Hz, 3H).		
**^{13}C-RMN (101 MHz, $CDCl_3$) δ (ppm)**		
149.86, 119.33, 50.68, 22.75.		
**IR υ (cm^{-1})**		
911 (=C-H)		

Starting material: 4-phenyl-1-butene[10]

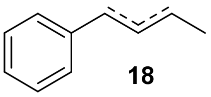 **18**	**Composition**	$C_{10}H_{12}$
	**Molecular weight (g/mol)**	132.20
	**Concentration cat. (mol%)**	0.001
	**Temperature (ºC)**	200
	**Yield (%) (2-alkene/3-alkene):**	95.3 (62.4/32.8)
**^{1}H-RMN (401 MHz, DMSO) δ (ppm)**		
3-Alkene: 7.25 – 7.20 (m, 5H), 6.41 – 6.37 (m, 2H), 2.27 – 2.20 (m, 2H), 1.09 (t, J = 7.4 Hz, 3H).<br>2-Alkene: 7.36 – 7.30 (m, 5H), 5.62 – 5.56 (m, 2H), 3.33 (d, J = 6.4 Hz, 2H), 1.69 (dd, J = 6.4, 1.2 Hz, 3H).		
**^{13}C-RMN (101 MHz, DMSO) δ (ppm)**		
3-Alkene: 137.83, 130.73, 129.08, 128.83, 128.65, 127.29, 25.94, 13.98.<br>2-Alkene: 141.10, 132.71, 128.97, 128.76, 126.24, 124.86, 38.77, 18.09.		
**IR υ (cm^{-1})**		
965.98 (=C-H)		

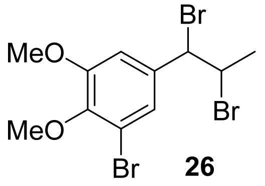 26	**Composition**	$C_{11}H_{13}Br_3O_2$
	**Molecular weight (g/mol)**	416.94
	**Yield (%)**	70
**^{1}H-RMN (401 MHz, CDCl$_3$,) δ (ppm):**		
6.93 (s, 1H), 6.91 (s, 1H), 5.49 (d, J = 10.1 Hz, 1H), 4.58 – 4.51 (m, 1H), 1.97 (d, J = 6.5 Hz, 3H).		
**^{13}C-RMN (101 MHz, CDCl$_3$) δ (ppm):**		
149.75, 148.91, 131.34, 114.96, 114.08, 111.12, 57.39, 56.17, 56.14, 50.42, 25.39.		
**IR υ (cm^{-1})**		
1260.19, 1239.31 (Ar-OMe),		

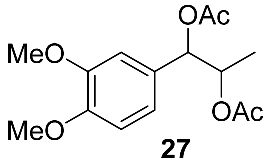 27	**Composition**	$C_{15}H_{20}O_6$
	**Molecular weight (g/mol)**	296.13
	**Yield (%)**	65
**^{1}H-RMN (401 MHz, CDCl$_3$,) δ (ppm):**		
6.83 (d, J = 2.0 Hz, 1H), 6.78 (d, J = 2.0 Hz, 1H), 6.77 (s, 1H), 5.62 (d, J = 7.8 Hz, 1H), 5.21 – 5.13 (m, 1H), 3.82 (s, 6H), 1.99 (s, 3H), 1.97 (s, 3H), 1.00 (d, J = 6.5 Hz, 3H).		
**^{13}C-RMN (101 MHz, CDCl$_3$) δ (ppm):**		
170.33, 169.97, 149.28, 149.05, 129.31, 120.24, 111.00, 110.30, 75.99, 71.53, 55.96, 55.87, 21.12, 21.06, 16.62.		
**IR υ (cm^{-1})**		
1736,58 (C=O), 1264.11, 1225.54 (Ar-OMe),		

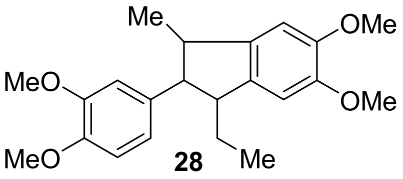 **28**		
	**Composition**	$C_{22}H_{28}O_4$
	**Molecular weight (g/mol)**	356.20
	**Yield (%)**	90

^{1}H-RMN (401 MHz, $CDCl_3$,) δ (ppm):

6.71 (t, J = 4.1 Hz, 2H), 6.61 (dd, J = 8.2, 1.9 Hz, 1H), 6.55 (d, J = 1.9 Hz, 1H), 6.34 (s, 1H), 3.78 (s, 3H), 3.75 (s, 3H), 3.71 (d, J = 2.3 Hz, 1H), 3.69 (s, 3H), 3.61 (s, 3H), 2.83 (ddd, J = 8.7, 7.4, 5.7 Hz, 1H), 2.42 – 2.31 (m, 1H), 1.65 – 1.55 (m, 1H), 1.35 – 1.23 (m, 1H), 0.95 (d, J = 7.0 Hz, 3H), 0.88 (t, J = 7.4 Hz, 3H).

^{13}C-RMN (101 MHz, $CDCl_3$) δ (ppm):

148.94, 148.17, 147.73, 147.61, 139.42, 138.13, 136.69, 120.81, 111.38, 111.01, 108.26, 108.16, 56.98, 56.03, 55.95, 55.81, 49.59, 48.54, 22.44, 13.79, 12.23.

IR υ (cm^{-1})

1262.18, 1250.61,1235.18, 1210,11 (Ar-OMe)

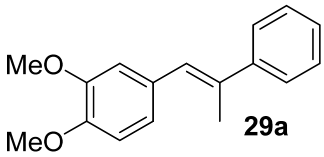 **29a**		
	**Composition**	$C_{17}H_{18}O_2$
	**Molecular weight (g/mol)**	254.13
	**Yield (%)**	65

^{1}H-RMN (300 MHz, $CDCl_3$,) δ (ppm):

7.47 – 7.41 (m, 2H), 7.31 – 7.25 (m, 2H), 7.22 – 7.16 (m, 2H), 6.86 – 6.79 (m, 2H), 6.72 – 6.64 (m, 1H), 3.83 (s, 6H), 2.22 (d, J = 1.3 Hz, 3H).

^{13}C-RMN (75 MHz, $CDCl_3$) δ (ppm):

148.58, 147.76, 144.12, 136.28, 131.31, 128.32, 127.50, 127.03, 125.95, 121.70, 112.53, 110.99, 55.93, 55.88, 17.57.

IR υ (cm^{-1})

1262.18, 1239.04 (Ar-OMe), 913.13 (=C-H)

<table>
<tr><td rowspan="3">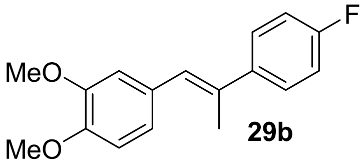
29b</td><td>Composition</td><td>$C_{17}H_{17}FO_2$</td></tr>
<tr><td>Molecular weight (g/mol)</td><td>272.12</td></tr>
<tr><td>Yield (%)</td><td>67</td></tr>
<tr><td colspan="3">^{1}H-RMN (300 MHz, $CDCl_3$,) δ (ppm):</td></tr>
<tr><td colspan="3">7.45 – 7.37 (m, 2H), 7.03 – 6.95 (m, 2H), 6.86 (d, J = 1.6 Hz, 1H), 6.84 (s, 1H), 6.83 (d, J = 1.5 Hz, 1H), 6.66 (s, 1H), 3.85 (s, 6H), 3.85 (s, 6H), 2.21 (d, J = 1.1 Hz, 3H).</td></tr>
<tr><td colspan="3">^{13}C-RMN (75 MHz, $CDCl_3$) δ (ppm):</td></tr>
<tr><td colspan="3">162.05 (d, J = 245.9 Hz), 148.58, 147.80, 140.14, 135.24, 131.09, 127.44 (d, J = 7.8 Hz), 121.64, 115.05 (d, J = 21.3 Hz), 112.47, 110.98, 55.90, 55.85, 17.65.</td></tr>
<tr><td colspan="3">^{19}F-RMN (282 MHz, DMSO) δ (ppm)</td></tr>
<tr><td colspan="3">-115.81 (tt, J = 8.6, 5.4 Hz, 1F).</td></tr>
<tr><td colspan="3">IR υ (cm^{-1})</td></tr>
<tr><td colspan="3">1264.11, 1220.72 (Ar-OMe), 921.81 (=C-H)</td></tr>
</table>

<table>
<tr><td rowspan="3">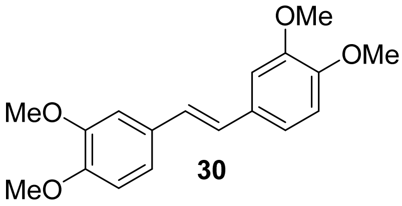
30</td><td>Composition</td><td>$C_{18}H_{20}O_4$</td></tr>
<tr><td>Molecular weight (g/mol)</td><td>300.14</td></tr>
<tr><td>Yield (%)</td><td>85</td></tr>
<tr><td colspan="3">^{1}H-RMN (401 MHz, $CDCl_3$,) δ (ppm):</td></tr>
<tr><td colspan="3">7.00 (d, J = 1.9 Hz, 2H), 6.97 (dd, J = 8.2, 1.9 Hz, 2H), 6.86 (s, 2H), 6.79 (d, J = 8.2 Hz, 2H), 3.89 (s, 6H), 3.84 (s, 6H).</td></tr>
<tr><td colspan="3">^{13}C-RMN (101 MHz, $CDCl_3$) δ (ppm):</td></tr>
<tr><td colspan="3">149.14, 148.71, 130.69, 126.65, 119.57, 111.27, 108.60, 55.96, 55.87.</td></tr>
<tr><td colspan="3">IR υ (cm^{-1})</td></tr>
<tr><td colspan="3">1262.18, 1262.17 ,1239.04, 1230,36 (Ar-OMe)</td></tr>
</table>

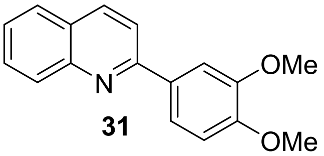 **31**	**Composition**	$C_{17}H_{15}NO_2$
	**Molecular weight (g/mol)**	265.11
	**Yield (%)**	65
**^{1}H-RMN (401 MHz, $CDCl_3$,) δ (ppm):**		
8.11 (t, J = 9.6 Hz, 2H), 7.82 (d, J = 2.1 Hz, 1H), 7.79 (d, J = 8.6 Hz, 1H), 7.75 (dd, J = 8.1, 1.1 Hz, 1H), 7.65 (ddd, J = 8.4, 6.9, 1.4 Hz, 1H), 7.61 (dd, J = 8.4, 2.1 Hz, 1H), 7.44 (ddd, J = 8.1, 6.9, 1.1 Hz, 1H), 6.94 (d, J = 8.4 Hz, 1H), 3.99 (s, 3H), 3.90 (s, 3H).		
**^{13}C-RMN (101 MHz, $CDCl_3$) δ (ppm):**		
156.83, 150.39, 149.40, 148.21, 136.61, 132.55, 129.58, 129.49, 127.42, 126.96, 125.98, 120.23, 118.60, 111.05, 110.45, 56.01, 55.99.		
**IR υ (cm^{-1})**		
1237.11, 1220.72 (Ar-OMe),		

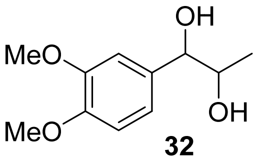 **32**	**Composition**	$C_{11}H_{16}O_4$
	**Molecular weight (g/mol)**	212.10
	**Yield (%)**	70
**^{1}H-RMN (401 MHz, $CDCl_3$,) δ (ppm):**		
6.80 (d, J = 1.7 Hz, 1H), 6.77 (d, J = 1.7 Hz, 1H), 6.76 (s, 1H), 4.22 (d, J = 7.5 Hz, 1H), 3.80 (s, 3H), 3.79 (s, 3H), 3.78 – 3.72 (m, 1H), 2.52 (s, 2H), 0.97 (d, J = 7.5 Hz, 3H).		
**^{13}C-RMN (101 MHz, $CDCl_3$) δ (ppm):**		
149.05, 148.85, 133.68, 119.30, 111.00, 109.71, 79.33, 72.23, 55.91, 55.90, 18.79.		
**IR υ (cm^{-1})**		
3394.10 (OH), 1259.29, 1230.36 (Ar-OMe),		

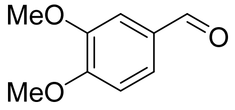 **33**	**Composition**	$C_9H_{10}O_3$
	**Molecular weight (g/mol)**	166.06
	**Yield (%)**	91

^{1}H-RMN (300 MHz, $CDCl_3$,) δ (ppm):

9.76 (s, 1H), 7.37 (dd, J = 8.2, 1.8 Hz, 1H), 7.32 (d, J = 1.8 Hz, 1H), 6.89 (d, J = 8.2 Hz, 1H), 3.88 (s, 3H), 3.85 (s, 3H).

^{13}C-RMN (75 MHz, $CDCl_3$) δ (ppm):

190.85, 154.47, 149.61, 130.13, 126.83, 110.38, 108.93, 56.15, 55.98.

IR υ (cm^{-1})

2833.88, 2759.64 (CHO), 1711.51 (C=O), 1257.36, 1230.36 (Ar-OMe),

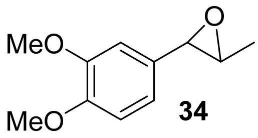 **34**	**Composition**	$C_{11}H_{14}O_3$
	**Molecular weight (g/mol)**	194.09
	**Yield (%)**	90

^{1}H-RMN (401 MHz, $CDCl_3$,) δ (ppm):

6.75 (d, J = 2.1 Hz, 1H), 6.74 (s, 1H), 6.64 (d, J = 1.7 Hz, 1H), 3.76 (d, J = 2.0 Hz, 3H), 3.43 (d, J = 2.1 Hz, 1H), 2.92 (qd, J = 5.1, 2.1 Hz, 1H), 1.33 (d, J = 5.2 Hz, 1H).

^{13}C-RMN (101 MHz, $CDCl_3$) δ (ppm):

149.22, 148.95, 130.26, 118.35, 111.09, 108.10, 59.52, 58.76, 55.93, 55.81, 17.82.

IR υ (cm^{-1})

1259.29, 1232.29 (Ar-OMe),

Supplementary References.

1. M. Mayer, W. M. Czaplik, A. J. von Wangelina, Practical Iron–Catalyzed Allylations of Aryl Grignard Reagents. *Adv. Synth. Catal.* 352, 2147−2152 (2010). doi: 10.1002/adsc.201000228

2. S. Mohottalage, R. Tabacchi, P. M. Guerin. Components from Sri Lankan Piper betle L. leaf oil and their analogues showing toxicity against the housefly, Musca domestica. *Flavour Fragr. J.* **22**, 130–138 (2007). doi: 10.1002/ffj.1770

3. R. Trivedi, J. A. Tunge, Etherification Regioselective Iron–Catalyzed Decarboxylative Allylic. *Org. Lett.* **11**, 5650−5652 (2009). doi: 10.1021/ol902291z

4. A. S. Vinogradov, V. I. Krasnov, V. E. Platonov, Organozinc Reagents from Polyfluoroarenes: Preparation and Reactions with Allyl Halides. Synthesis of Allylpolyfluoroarenes. *Russ. J. Org. Chem.* **44**, 95−102 (2008). doi: 10.1134/S1070428008010119

5. A. Bouziane, M. Hélou B. Carboni, F. Carreaux, B. Demerseman, C. Bruneau, J.–L. Renaud, Ruthenium–Catalyzed Synthesis of Allylic Alcohols: Boronic Acid as a Hydroxide Source. *Chem. Eur. J.* **14**, 5630–5637 (2008). doi: 10.1002/chem.200702030

6. D. R. Boyd, D. Clarke, M. C. Cleij, J. T. G Hamilton, G. N. Sheldrake, Bacterial Biotransformation of Isoprene and Related Dienes. *Monatshefte für Chemie* **131**, 673−685 (2000). doi: 10.1007/s007060070096

7. B. W. Parks, R. D. Gilbertson, D. W. Domaille, J. E. Hutchison, Convenient Synthesis of 6,6–Bicyclic Malonamides: A New Class of Conformationally Preorganized Ligands for f–Block Ion Binding. *J. Org. Chem.* **71**, 9622−9627 (2006). doi: 10.1021/jo0617262

8. F. Felluga, C. Forzato, F. Ghelfi, P. Nitti, G. Pitacco, U. M. Pagnoni, F. Roncaglia, Atom transfer radical cyclization (ATRC) applied to a chemoenzymatic synthesis of Quercus lactones. Tetrahedron: Asymmetry **18**, 527–536 (2007). doi: 10.1016/j.tetasy.2007.02.012

9. M. Yasuda, T. Saito, M. Ueba, A. Baba, Direct Substitution of the Hydroxy Group in Alcohols with Silyl Nucleophiles Catalyzed by Indium Trichloride. *Angew. Chem.* **116**, 1438–1440 (2004). doi: 10.1002/ange.200353121

10. H. Lebel and C. Ladjel, Iridium Complexes in Olefination Reactions. *Organometallics* **27**, 2676–2678 (2008). doi: 10.1021/om800255c
